# TRIB3 Links Endoplasmic Reticulum Stress to Impaired Efferocytosis in Atherosclerosis

**DOI:** 10.1161/CIRCRESAHA.125.326839

**Published:** 2025-11-04

**Authors:** Aarushi Singhal, Stefan Russo, Umesh Kumar Dhawan, Kunzangla Bhutia, Christopher G. Bell, Hedayatullah Hayat, Thomas D. Nightingale, Monica de Gaetano, Orina Belton, Eoin Brennan, Patricia B. Munroe, Catherine Godson, Mary Barry, Carol C. Shoulders, Heather L. Wilson, Guillermo Velasco, Endre Kiss-Toth, Manikandan Subramanian

**Affiliations:** 1 William Harvey Research Institute, Faculty of Medicine and Dentistry, Queen Mary University of London, United Kingdom (A.S., S.R., U.K.D., C.G.B., H.H., T.D.N., P.B.M., C.C.S., M.S.).; 2 Department of Biochemistry and Molecular Biology, School of Biology, Complutense University and Instituto de Investigación Sanitaria, San Carlos (IDISSC), Madrid, Spain (K.B., G.V.).; 3 School of Biomolecular and Biomedical Science (M.d.G., O.B.), University College Dublin, Ireland.; 4 School of Medicine (E.B., C.G.), University College Dublin, Ireland.; 5 Department of Vascular Surgery, St. Vincent’s University Hospital, Dublin, Ireland (M.B.).; 6 Division of Clinical Medicine, School of Medicine and Population Health, University of Sheffield, United Kingdom (H.L.W., E.K.-T.).

**Keywords:** atherosclerosis, efferocytosis, endoplasmic reticulum, macrophages

## Abstract

**BACKGROUND:**

Defective macrophage efferocytosis is a key driver of chronic nonresolving inflammation in dyslipidemia-associated diseases, such as obesity and atherosclerosis. However, the mechanism by which intracellular lipid accumulation impairs macrophage efferocytosis remains unclear. We hypothesized that lipid-induced endoplasmic reticulum (ER) stress mediates defective macrophage efferocytosis.

**METHODS:**

Bone marrow–derived macrophages were exposed to 7-ketocholesterol or palmitate to induce ER stress, and efferocytosis was quantified by measuring uptake of fluorescently labeled apoptotic cells with microscopy and flow cytometry. Key pathways were interrogated with pharmacological inhibitors, siRNA (silencing RNA), and in vivo models, including obese mice and in *Ldlr*^*−/−*^ mice with hematopoietic-specific deletion of TRIB3 (Tribbles pseudokinase-3). Human relevance was assessed by testing efferocytosis in macrophages from individuals carrying the TRIB3 Q84R coronary artery disease risk variant (rs2295490) and by examining carotid endarterectomy samples.

**RESULTS:**

Activation of the ATF4 (activating transcription factor 4) branch of the ER stress pathway in lipid-loaded foamy macrophages led to upregulation of TRIB3, which triggered the downregulation of Rab27a, resulting in impaired focal exocytosis of intracellular membrane pools towards nascent, apoptotic cell–containing phagosomes. The resultant delay in phagosome closure stalled efferocytosis. In obese mice, this impairment was reversed using an ER stress–relieving chemical chaperone and via macrophage-specific knockdown of ATF4 or TRIB3. In atherosclerotic mice, hematopoietic cell–specific deletion of TRIB3 led to increased lesional efferocytosis, decreased plaque necrosis, and increased collagen, which are characteristic of stable plaques. In humans, TRIB3 expression was higher in vulnerable regions of carotid plaques, and macrophages from individuals carrying the gain-of-function TRIB3 Q84R risk variant expressed more TRIB3 and displayed decreased efferocytosis.

**CONCLUSIONS:**

Lipid-induced ER stress impairs macrophage efferocytosis via activation of the ATF4-TRIB3-Rab27a signaling axis, leading to exacerbated plaque necrosis. Targeted disruption of TRIB3 signaling in macrophages represents a novel therapeutic approach to promote efferocytosis and stabilize atherosclerotic plaques.

**Novelty and Significance**

**What Is Known?**

- Defective macrophage efferocytosis contributes to chronic, nonresolving inflammation and plaque necrosis in atherosclerosis.
- Lipid accumulation and chronic endoplasmic reticulum stress are hallmarks of macrophage foam cell formation, but how these events impair clearance of apoptotic cells is unclear.

**What New Information Does This Article Contribute?**

- Lipid-induced endoplasmic reticulum stress in obesity and atherosclerosis activates the ATF4 (activating transcription factor 4)–TRIB3 (Tribbles pseudokinase-3) signaling axis in macrophages, which represses Rab27a-mediated focal exocytosis required for efficient efferocytosis.
- Suppression of TRIB3 signaling restores efferocytosis, reduces necrotic core formation, and stabilizes atherosclerotic plaques in mice.
- In humans, the coronary artery disease–associated TRIB3 Q84R gain-of-function variant is linked to increased TRIB3 expression and impaired macrophage efferocytosis.

This study identifies a mechanistic link between lipid-induced endoplasmic reticulum stress and defective macrophage efferocytosis, mediated through the ATF4-TRIB3-Rab27a axis. By establishing TRIB3 as a key negative regulator of efferocytosis, these findings explain how metabolic stress disrupts inflammation resolution and compromises atherosclerotic plaque stability. Targeting TRIB3 signaling may therefore represent a promising therapeutic strategy to enhance macrophage clearance of dead cells and stabilize atherosclerotic lesions.

**Meet the First Author, see p 1381**

Apoptotic cells (ACs) arising from both physiological and pathological circumstances undergo rapid clearance by macrophages through a specialized phagocytic process known as efferocytosis.^1^ This prompt removal of ACs is crucial for averting secondary necrosis of ACs and the ensuing inflammatory response.^1^ Consequently, defective macrophage efferocytosis is linked with various metabolic,^2^ autoimmune,^3^ and chronic inflammatory conditions,^4^ including obesity and atherosclerotic vascular disease, where it exacerbates plaque necrosis and leads to adverse clinical outcomes such as myocardial infarction and stroke.^5–7^ However, the precise molecular and cellular mechanisms underlying impaired efferocytosis under conditions of atherogenic dyslipidemia and metabolic perturbations remain inadequately understood. These insights will be crucial for the development of novel adjunct therapeutics to address the residual inflammatory risk driving morbidity and mortality in patients with atherosclerotic cardiovascular disease treated with cholesterol-lowering therapies such as statins.^4,8^

Dyslipidemia is associated with the accumulation of cholesterol species within vascular wall macrophages and other tissue macrophages, resulting in the formation of foamy macrophages.^9^ This buildup of lipids leads to disruption of endoplasmic reticulum (ER) membrane dynamics, perturbations in Ca^2+^ homeostasis, and generation of reactive oxygen species, which together induce ER stress^10,11^ and the accumulation of unfolded proteins within the ER. This scenario triggers the induction of an adaptive and protective signaling cascade known as the unfolded protein response (UPR) which involves the coordinated activation of IRE1α (inositol-requiring enzyme 1α), PERK (protein kinase RNA-like endoplasmic reticulum kinase or eukaryotic translation initiation factor 2 alpha kinase 3)–ATF4 (activating transcription factor 4), and ATF6 pathways which aims to restore ER and cellular homeostasis.^12^ However, when this adaptive mechanism fails, uncontrolled ER stress activates apoptotic signaling pathways, leading to cell death.^13,14^ This induction of cell death, when coupled with a diminished pool of phagocytes to mediate efferocytosis, contributes to the expansion of the necrotic core within atherosclerotic plaques.^6^ Notably, genetic deficiencies in ER stress response effectors, such as CHOP (C/EBP homologous protein),^15^ the use of chemical chaperones, such as 4-phenylbutyric acid (4-PBA), to reverse ER stress,^16^ or the use of specific UPR inhibitors targeting the IRE1 pathway^17,18^ leads to reduced lesional necrosis and stabilization of atherosclerotic plaques.

Although there is compelling evidence implicating ER stress in exacerbating plaque necrosis,^18–21^ it remains unclear whether this is primarily driven by enhanced macrophage death and subsequent loss of an efferocytosis-competent phagocyte pool or if ER stress directly impairs macrophage efferocytosis. Recent studies have suggested that macrophages experiencing acute severe ER stress exhibit defective efferocytosis,^18,22,23^ yet the pathophysiological relevance of these findings in the context of atherosclerosis in vivo is uncertain, as most plaque macrophages experience chronic adaptive ER stress,^14,24,25^ a state characterized by activation of UPR signaling without affecting cellular viability. In addition, the cellular and molecular mechanisms by which ER stress impairs efferocytosis are not well understood and, therefore, represent a critical knowledge gap that impedes our ability to develop novel therapeutic strategies to enhance efferocytosis in disorders associated with ER stress, such as atherosclerosis, obesity, chronic obstructive pulmonary disease, inflammatory bowel disease, and neurodegenerative disorders.^26^

In this study, using a combination of in vitro and in vivo models of ER stress in macrophages, we demonstrate that ER stress diminishes efferocytosis efficiency via activation of the PERK-ATF4 branch of the UPR pathway leading to upregulation of TRIB3 (Tribbles pseudokinase-3), a protein with roles in regulating cell signaling, insulin secretion, lipid metabolism, immune function, cell cycle, cell proliferation, and transcriptional repression.^27–31^ Mechanistically, our data reveal that TRIB3 decreases efferocytosis via downregulation of Rab27a, a protein involved in focal exocytosis, resulting in impaired phagosome closure.^32^ In vivo, we show that ablating macrophage-TRIB3 reverses the impairment of efferocytosis in mouse models of obesity and atherosclerosis. Furthermore, we show that humans with the coronary artery disease (CAD) risk variant of TRIB3 (rs2295490-G) demonstrate increased TRIB3 expression and decreased macrophage efferocytosis efficiency. These findings underscore the critical role of the ATF4-TRIB3 signaling axis in impairing efferocytosis in atherosclerosis and other dyslipidemic conditions, suggesting that therapeutics targeting this pathway could promote efferocytosis and accelerate the resolution of inflammation.

## Methods

### Data Availability

Data supporting the findings in this study are available from the corresponding author on reasonable request. A list of major resources used is available in the Major Resources Table in Supplemental Material.

### Animal Procurement and Maintenance

Male and female C57BL/6J and male *Ldlr*^*−*^^*/*^^*−*^ (stock no. 002207) mice were purchased from Charles River and Jackson Laboratories, respectively, and maintained at the Queen Mary University of London’s Biological Service Unit in Charterhouse Square campus. *Trib3*^*−/−*^ mice were generated as described previously.^33^ The mice were housed in individually ventilated cages with a 12-hour light-dark cycle and had access to ad libitum water and food. All mice were randomly assigned to experimental procedures. All animal procedures were conducted as per ethical guidelines after obtaining approval from the Home Office, United Kingdom.

### Bone Marrow Chimeric Mice Generation and Induction of Atherosclerosis

Eight-week-old male *Ldlr*^*−/−*^ mice were subjected to 10 Gy X-ray irradiation followed by bone marrow reconstitution via intravenous injection of 5×10^6^ bone marrow cells from either wild-type or TRIB3 knockout male mice. The mice were allowed 6 weeks for recovery and the development of bone marrow chimerism, followed by induction of atherosclerosis by feeding them a Western-type diet (SDS western diet, 829100) for 14 weeks. Only male *Ldlr*^*−/−*^ mice were used in this study to avoid rejection of sex mismatched donor bone marrow cells.

### In Vivo Efferocytosis Assay in siRNA-Transfected Lean and Obese Mice

Six-week-old male C57BL/6J mice were fed a high-fat diet (D12492; Research Diets) for 10 weeks to induce obesity, whereas control lean mice were maintained on a standard chow diet. Only male mice were used in this experiment, because female C57BL/6J mice show resistance to high-fat diet–induced obesity and associated metabolic phenotypes.^34,35^ For siRNA (silencing RNA)-mediated *Trib3* knockdown, siRNA complexes were prepared by separately mixing negative control siRNA or *Trib3* siRNA (20 μg) with jetPEI-Man (3.2 μL, N/P ratio of 8) in 5% glucose solution, followed by incubation at room temperature for 15 minutes. The complexes (400 μL) were then administered intraperitoneally. Forty-eight hours posttransfection, mice received an intraperitoneal injection of fluorescently labeled ACs. One hour later, peritoneal lavage was performed, and efferocytosis efficiency was assessed by flow cytometry on F4/80⁺ peritoneal macrophages.

### In Vitro Efferocytosis Assay

Macrophages were incubated with fluorescently labeled ACs at a ratio of 1:3 (macrophages: ACs) for 1 hour, followed by 2 washes with PBS to remove nonengulfed ACs. Fluorescence microscopy was performed using the EVOS FLoid cell imaging system. Data were analyzed from 10 fields of view per group, and the efferocytosis efficiency was quantified as the percentage of macrophages containing at least 1 fluorescently labeled AC.

### Phagosome Sealing Assay

Cells that were to be rendered apoptotic were first subjected to cell surface protein biotinylation using EZ-link sulfo-NHS-biotin (0.5 mmol/L in PBS pH 8.0, at 37 °C for 30 minutes), followed by PKH67 labeling as described above. Apoptosis was induced by exposure to ultraviolet. Dual-labeled ACs were incubated with macrophages at a ratio of 1:3 (macrophage:AC) for 1 hour, followed by 2 washes with PBS to remove nonbound ACs. The cells were fixed with 4% PFA followed by labeling with streptavidin conjugated to Alexa Fluor 594 at a dilution of 1:200 in PBS for 30 minutes in the dark at room temperature. Fluorescence microscopy was conducted, and the numbers of sealed phagosomes (defined as macrophages containing a green-only labeled AC) and unsealed phagosomes (defined as macrophages containing green+red–labeled AC) were quantified.

### Focal Exocytosis Assay

Macrophages were stained with the styryl dye FM1-43 (10 µmol/L) in serum-free media for 1 hour, followed by 2 washes with PBS, as described previously.^36^ The cells were placed in serum-containing media for 30 minutes for dye to desorb from the cell surface before addition of pHrodo deep red–labeled ACs at ratio of 2:1 (macrophage:AC). The cells were placed in an environmentally controlled chamber at 37 °C with 5% CO_2_, and live confocal microscopic imaging was conducted for 3 hours while capturing 9 fields of view per group at an interval of 5 minutes in a Nikon spinning disk confocal microscope. Analysis was done using Fiji (ImageJ). Focal exocytosis was quantified by measuring the change in fluorescence intensity of the FM1-43 signal over 20 minutes from the time of contact with an AC, which was defined by the time of appearance of the pHrodo signal. A minimum of 35 cells were used for quantification per group per replicate.

### Human Carotid Endarterectomy Plaques

Studies were approved by St. Vincent’s University Hospital, Dublin Ethics Committee, and adhered to international guidelines and the Declaration of Helsinki principles as revised in 2008. All participants provided informed written consent. Endarterectomy samples were obtained from consenting patients of mixed sexes postrevascularization surgery and dissected as described previously^37^ into relatively disease-free (stable) and diseased plaque (vulnerable) from internal carotid portions and were snap-frozen for further processing and analysis.

### Statistical Analysis

All data analysis was conducted in GraphPad Prism 10. The data are represented as mean±SEM, unless indicated otherwise. For in vivo experiments, n signifies the number of mice utilized per group for experimental purposes. For in vitro experiments, n indicates the number of independent experiments. For experiments with sample sizes >6, normality was assessed using the Shapiro-Wilk test. Comparisons between 2 normally distributed data sets were performed using an unpaired Student *t* test, whereas non-normally distributed data sets were analyzed using the Mann-Whitney *U* test. For comparisons involving >2 groups, the Kruskal-Wallis test with Dunn post hoc correction was applied. A *P*<0.05 was considered statistically significant. The statistical test used for each analysis is specified in the figure legends.

## Results

### Lipid Accumulation in Macrophages Impairs Efferocytosis via Activation of ER Stress

**Figure 1.**
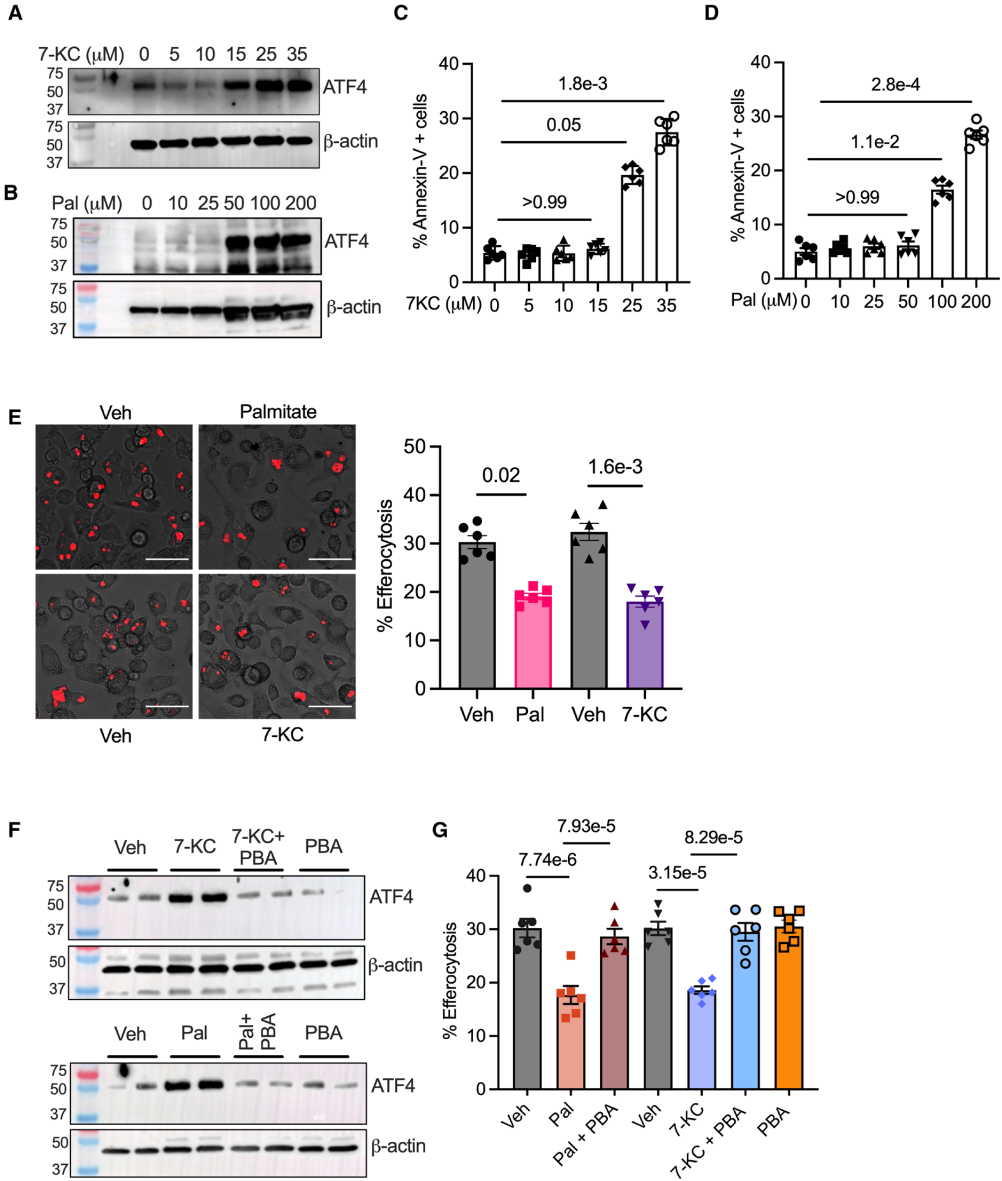
**Endoplasmic reticulum stress impairs macrophage (Mϕ) efferocytosis in vitro. A**, Murine bone marrow–derived Mϕs (BMDMs) were incubated with indicated concentrations of 7-ketocholesterol (7-KC) or (**B**) palmitate (Pal) for 18 hours, followed by immunoblotting for quantification of ATF4 (activating transcription factor 4) levels in whole cell lysates. β-actin was used as a loading control. **C** and **D**, As above, except that the 7-KC or Pal-treated cells were stained with annexin-V fluorescein isothiocyanate for analysis of viability by fluorescence microscopy. **E**, BMDMs were exposed to either vehicle (Veh), Pal (50 µmol/L), or 7-KC (15 µmol/L) for 18 hours, followed by incubation with pHrodo red–labeled apoptotic cells (ACs; red) at a Mϕ:AC ratio of 1:3 for 1 hour. Efferocytosis efficiency was calculated as percent Mϕs that contained at least 1 pHrodo red–labeled AC. n=6 biological replicates from 2 independent experiments. Scale bar, 20 µm. **F**, Immunoblotting for ATF4 in whole cell lysates of Mϕs incubated with either 7-KC or Pal in the absence or presence of 4-phenylbutyric acid (4-PBA, 1 mmol/L) for 18 hours. **G**, Similar to (**E**), except that 7-KC–treated and palmitate-treated Mϕs were coincubated with 1 mmol/L 4-PBA before conducting the efferocytosis assay. n=6 biological replicates from 2 independent experiments. Statistical analysis was performed using Kruskal-Wallis and Dunn multiple comparisons test (**C**, **D**, **E**, and **G**).

Because previous studies primarily focused on acute severe ER stress,^18,22,23^ we first set up an in vitro model to mimic chronic adaptive ER stress in macrophages, such as that found in dyslipidemic environments associated with atherosclerotic plaques and tissues in obesity. Murine bone marrow–derived macrophages were incubated for 18 hours with a range of concentrations of 7-ketocholesterol (7-KC, 5–35 µmol/L), an oxysterol which is enriched in atherosclerotic plaques,^38,39^ or the saturated fatty acid palmitate (10–200 µmol/L). Oil red O staining confirmed an increase in the lipid droplet content in macrophages exposed to 7-KC or palmitate, consistent with an accumulation of intracellular esterified lipids and the generation of foamy macrophages (Figure S1A and S1B). Next, to check for activation of ER stress, we conducted Western blotting to quantify protein levels of the ER stress markers ATF4 and XBP-1s (spliced X-box–binding protein 1). As shown (Figure 1A and 1B; Figure S1C), 15 µmol/L 7-KC and 50 µmol/L palmitate were the lowest concentrations that increased ATF4 and XBP-1s protein levels. In addition, both 7-KC and palmitate elevated PERK levels (Figure S1D), the upstream activator of ATF4, and enhanced IRE1 endoribonuclease activity mediated splicing of XBP1 (Figure S1E). Importantly, despite the induction of ER stress at these concentrations, there was no associated increase in cell death as measured by Annexin-V staining (Figure 1C and 1D). Taken together, these data demonstrate that exposure of murine bone marrow–derived macrophages to either 15 µmol/L 7-KC or 50 µmol/L palmitate for 18 hours produced an in vitro model to study the effect of chronic adaptive ER stress on macrophage efferocytosis efficiency.

To address the question of whether adaptive ER stress in macrophages affects efferocytosis, we incubated 7-KC, or palmitate-exposed macrophages, with AC that had been previously labeled with pHrodo, a nonfluorescent dye that dramatically increases its fluorescence at the low pH of phagolysosomes. Thus, efferocytosis efficiency can be calculated by quantifying the percentage of macrophages that engulf at least 1 AC. Relative to control macrophages, both 7-KC and palmitate-exposed macrophages showed a decrease in efferocytosis (Figure 1E) suggesting that chronic adaptive ER stress decreases efferocytosis efficiency in vitro. Because 7-KC and palmitate produce pleiotropic cellular effects, we specifically tested whether the decrease in efferocytosis after the exposure of macrophages to 7-KC and palmitate is dependent on the induction of ER stress. To this end, macrophages were coincubated with 4-PBA, a chemical chaperone that is known to prevent ER stress (Figure 1F).^16^ Critically, 4-PBA was able to rescue the 7-KC and palmitate-induced defect in macrophage efferocytosis (Figure 1G). A similar decrease in efferocytosis in macrophages exposed to 7-KC and palmitate was observed by flow cytometric analysis, which was rescued on coincubation with 4-PBA (Figure S1F). This prevention is unlikely to be an off-target effect because similar findings were obtained (Figure S1G) when macrophages were coincubated with a second chemical chaperone, tauroursodeoxycholate.^40^ In addition, efferocytosis was similarly impaired in macrophages subjected to chemical-induced ER stressors, such as thapsigargin, tunicamycin, and dithiothreitol (Figure S1H). Taken together, these data demonstrate that chronic adaptive ER stress impairs efferocytosis efficiency of macrophages in vitro.

### Alleviation of ER Stress Improves Macrophage Efferocytosis Efficiency and Accelerates Inflammation Resolution In Vivo in Obese Mice

**Figure 2.**
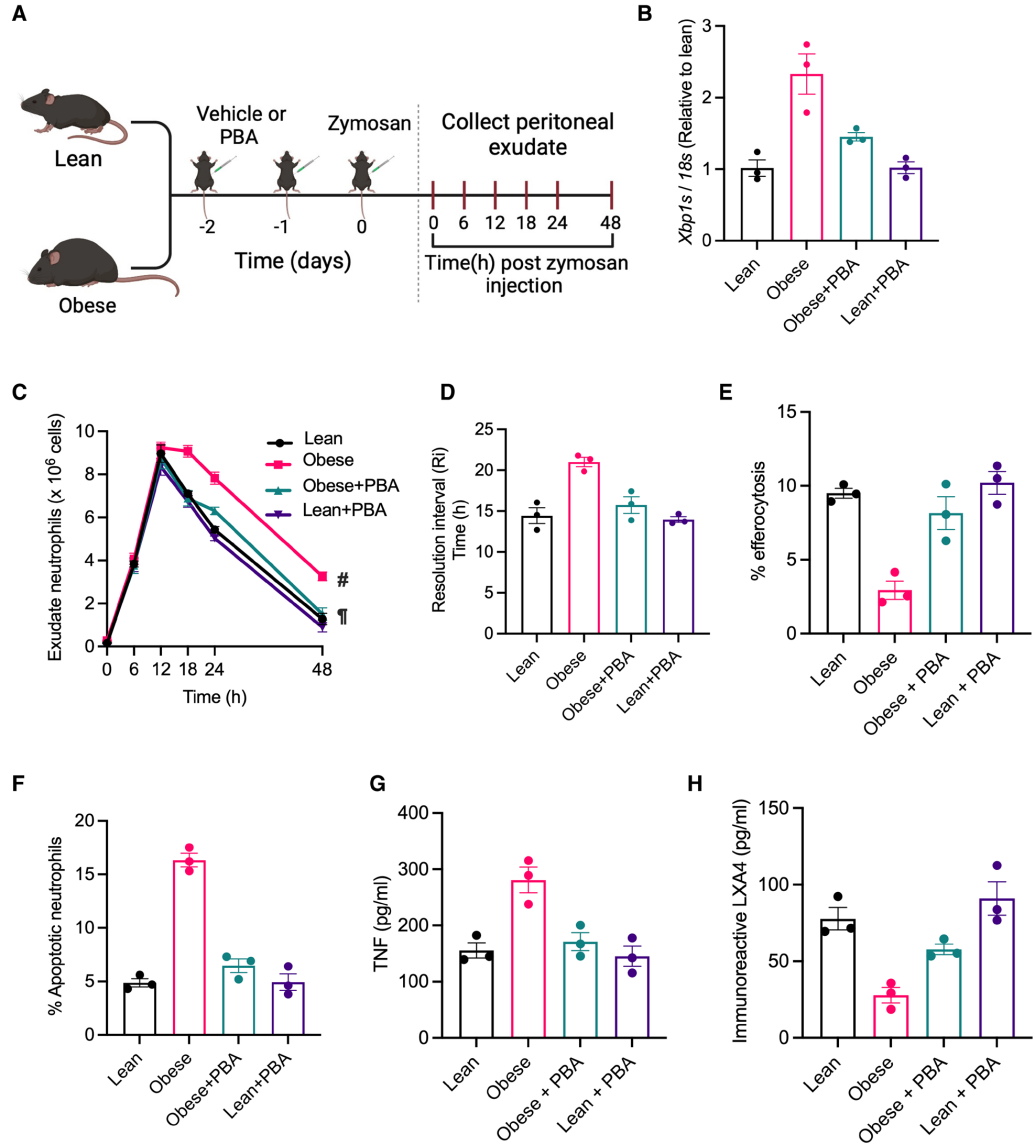
**Obesity-induced endoplasmic reticulum stress impairs macrophage (Mϕ) efferocytosis and inflammation resolution. A**, Schematic representation of the experimental strategy. Lean and obese (10 weeks high-fat diet–fed) male C57BL/6J mice received a daily intraperitoneal injection (IP) of either 4-phenylbutyric acid (4-PBA, 40 mg/kg) or PBS (vehicle) for 2 days before receiving 1 mg zymosan (IP). Exudates were collected by peritoneal lavage from 3 mice per group euthanized at 0, 6, 12, 18, 24, and 48 hours postzymosan injection. **B**, RT-qPCR (real-time quantitative polymerase chain reaction) for analysis of *Xbp1s* levels in Mϕs isolated by plate adhesion from peritoneal lavage obtained at 2 days post–4-PBA administration (0 hour) in the indicated groups of mice. 18s was used as a housekeeping gene, and the data are plotted as relative expression compared with the lean group. **C** through **F**, Peritoneal exudate cells were subjected to flow cytometric analysis of neutrophil numbers (**C**), resolution interval (Ri; **D**), Mϕ efferocytosis efficiency (**E**), and the proportion of Annexin-V+ neutrophils (**F**). **G** and **H**, ELISA was conducted for measurement of TNF (tumor necrosis factor) and immunoreactive LXA4 (lipoxin A4) levels in peritoneal exudate fluid collected at 24 hours (**G** and **H**, respectively). All data are represented as mean±SEM.

To examine whether lipid accumulation-induced ER stress impairs macrophage efferocytosis in vivo, we used a mouse model of high-fat diet–induced obesity (Figure 2A, schematic) characterized by accumulation of neutral lipids in peritoneal cavity macrophages (Figure S2A). In this model, *Xbp1s* RNA levels were higher in the peritoneal cavity macrophages of the obese than in the lean mice (Figure 2B), consistent with induction of a lipid-induced ER stress response. This increase in *Xbp1s* was suppressed by the intraperitoneal administration of the chemical chaperone 4-PBA (Figure 2A and 2B), demonstrating its ability to alleviate ER stress in vivo in this model. To examine the efferocytosis efficiency of ER-stressed versus non-ER–stressed obese peritoneal cavity macrophages in vivo, we turned to a zymosan-induced acute peritonitis model.^41^ Here, the injection of zymosan intraperitoneally elicits rapid infiltration of neutrophils which then undergo cell death locally followed by their clearance by macrophage-mediated efferocytosis, leading to spontaneous resolution of inflammation within 48 hours.^41^ In this model, peritoneal exudate cells and soluble mediators were collected at various time points postinduction of inflammation for the quantification of macrophage efferocytosis efficiency and the kinetics of inflammation resolution (Figure 2A, schematic). First, we observed that the neutrophil numbers were similar between all groups up to 12 hours postzymosan injection, suggesting similar induction of inflammatory response between the groups (Figure 2C). However, the rate of decrease in neutrophil numbers over time was slower in the obese mice than the lean mice, which was not the case in the obese mice that had received 4-PBA (Figure 2C; Figure S2B). The time taken for the neutrophil numbers to reach half-maximal peak values (resolution interval) in the obese mice (21 hours) was longer than in the lean (14.4 hours) mice (Figure 2D), indicating that the obese mice had an impaired inflammation resolution response to zymosan. Notably, 4-PBA reduced the resolution interval in the obese, but not in the lean mice (Figure 2D). Because previous studies have indicated that a decline in neutrophil numbers is primarily determined by macrophage-mediated clearance of dying neutrophils, we quantified efferocytosis efficiency by analyzing the percentage of peritoneal macrophages (F4/80+) containing a Ly6G+ neutrophil by flow cytometry (Figure S2C). Fluorescence microscopy of fluorescence-activated cell sorter sorted F4/80+Ly6G+ cells showed the presence of Ly6G signal within the macrophage, demonstrating that these are bona fide efferocytosis events (Figure S2D). Notably, obese mice macrophages demonstrated a decrease in efferocytosis efficiency as compared with lean macrophages (Figure 2E; Figure S2C and S2E). Most importantly, the administration of 4-PBA rescued this defect in efferocytosis efficiency (Figure 2E; Figure S2C and S2E). Consistent with a defect in clearance of dying cells, obese mice demonstrated an accumulation of apoptotic neutrophils in the peritoneal exudate, which was again prevented by the administration of ER stress reliever 4-PBA (Figure 2F; Figure S2F). Finally, corroborating the findings that the defect in inflammation resolution response in obese mice is mediated by macrophage ER stress, we observed that obese mice had elevated levels of the proinflammatory cytokine TNF (tumor necrosis factor) and a decrease in levels of the proresolving lipid mediator lipoxin A4 (immunoreactive LXA4 [lipoxin A4] detected by ELISA) as compared with the lean mice which again could be reversed by treatment with 4-PBA (Figure 2G and 2H). Because inflammation resolution kinetics and macrophage efferocytosis were not altered by administering 4-PBA to lean mice (Figure 2C), these data suggest that 4-PBA improves macrophage efferocytosis and inflammation resolution in vivo in obese mice primarily via alleviation of ER stress.

### Defective Focal Exocytosis in ER-Stressed Macrophages Impairs Efferocytosis

**Figure 3.**
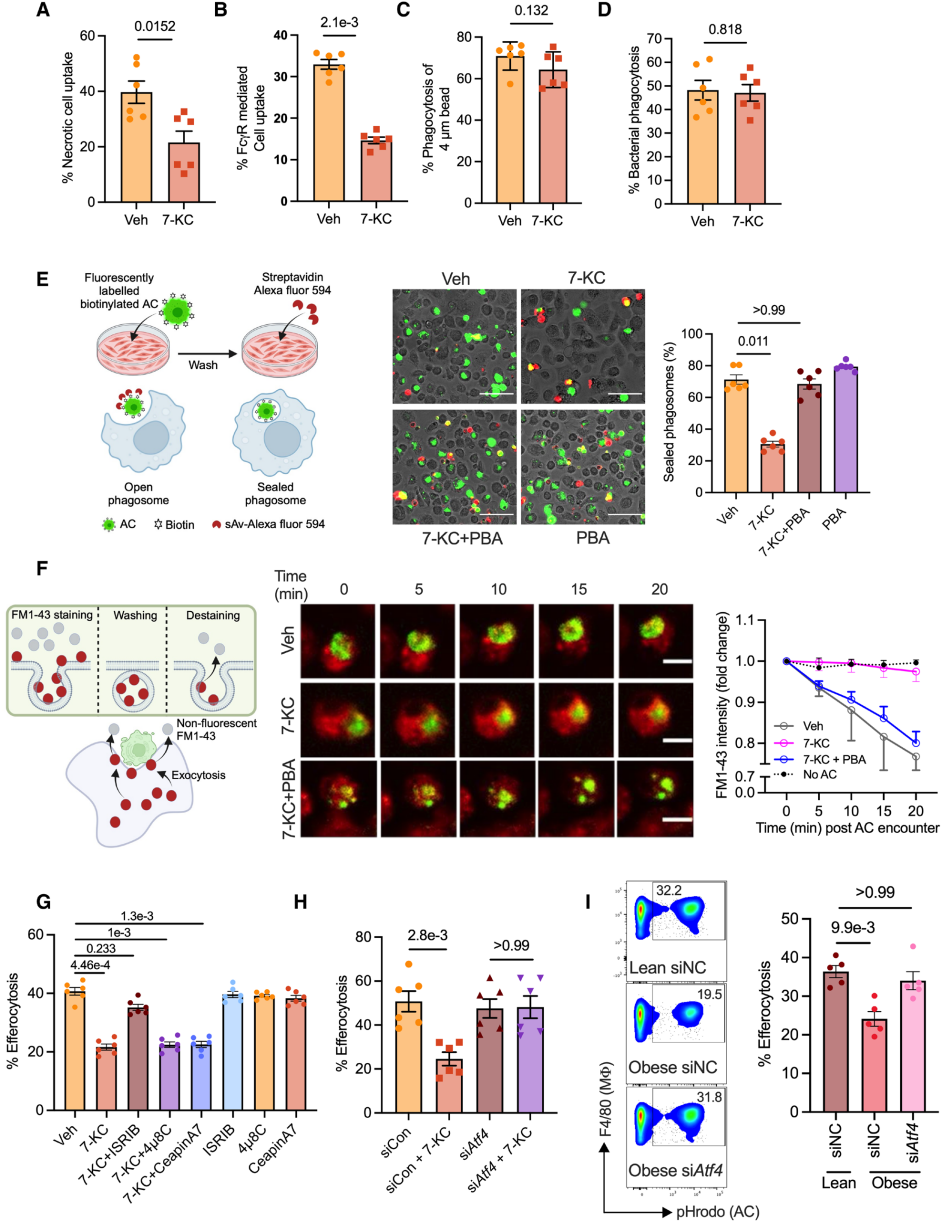
**Endoplasmic reticulum–stressed macrophages (Mϕs) display impaired focal exocytosis and delayed phagosome closure.** Fluorescence microscopic analysis was conducted to measure phagocytosis efficiency in bone marrow–derived macrophages (BMDMs) treated with either vehicle (Veh) or 15 µmol/L 7-ketocholesterol (7-KC) for 18 hours followed by incubation for 1 hour with fluorescently labeled necrotic cells (**A**), IgG-coated live cells (**B**), 4 µm latex beads (**C**), or *Escherichia coli* bioparticles (**D**). n=6 biological replicates from 2 independent experiments. **E**, Mϕs exposed to 15 µmol/L 7-KC for 18 hours in the absence or presence of 4-phenylbutyric acid (4-PBA) were incubated with biotinylated and PKH67-labeled apoptotic cells (ACs) at a ratio of 1:3 for 1 hour. Unengulfed ACs were removed by washing. The cells were fixed with 4% PFA, incubated with streptavidin Alexa Fluor 594 for 30 minutes, and imaged by fluorescence microscopy. Representative merged images from PKH67 (green) and streptavidin Alexa Fluor 594 (red) channels are shown. Scale bar, 20 µm. The percent of AC-containing phagosomes that are sealed (green only) was quantified. n=6 biological replicates with averages obtained from technical duplicates from 5 fields of view per replicate. **F**, FM1-43–labeled Mϕs (red) were incubated with pHrodo-labeled ACs (green, when engulfed by Mϕ) at a Mϕ:AC ratio of 2:1 followed by live imaging in a spinning disk confocal microscope. Measurement of Mϕ FM1-43 signal intensity was initiated from the first appearance of pHrodo signal (*t*=0 minute) and followed up every 5 minutes for 20 minutes. The data were plotted as fold change in FM1-43 intensity compared with *t*=0 minutes. n=3 biological replicates, with averages obtained from quantification from at least 35 cells. Scale bar, 10 µm. **G**, BMDMs were exposed to Veh or 7-KC for 18 hours in the presence or absence of ISRIB (integrated stress response inhibitor; 1 µmol/L), 4µ8C (1 µmol/L), or CeapinA7 (5 µmol/L) as indicated, followed by incubation with pHrodo-labeled ACs for 1 hour. Efferocytosis efficiency was quantified by fluorescence microscopy. n=6 biological replicates. **H**, BMDMs were transfected with either activating transcription factor 4 siRNA (si*Atf4*) or negative control siRNA (siCon). Forty-eight hours posttransfection, Mϕs were exposed to Veh or 7-KC for 18 hours and then incubated with pHrodo-labeled ACs for 1 hour. Efferocytosis efficiency was quantified by fluorescence microscopy. n=6 biological replicates from 2 independent experiments. **I**, Lean or obese mice peritoneal Mϕs were transfected with siNC (negative control siRNA) or *Atf4* siRNA (si*Atf4*), followed by injection of fluorescently labeled ACs. The peritoneal lavage was collected 1 hour later, and Mϕ efferocytosis efficiency was analyzed by flow cytometry. n=5 mice per group. All data are represented as mean±SEM. The data were analyzed for statistical significance using the Mann-Whitney *U* test (**A** through **D**) or Kruskal-Wallis with Dunn multiple comparisons test (**E**, **G** through **I**). FCɣR indicates Fc gamma receptor; sAv-Alexa fluor 594, streptavidin conjugated to alexa fluor 594; and siRNA, silencing RNA.

Having established that lipid-induced ER stress impairs macrophage efferocytosis efficiency in vitro and in vivo, we next explored the cellular mechanisms underpinning this process. Firstly, we confirmed that the control and ER-stressed macrophages were equally efficient at binding ACs (Figure S3A) and had comparable levels of key efferocytosis receptors, including Mertk (Mer tyrosine kinase), and LRP-1 (low-density lipoprotein receptor–related protein 1; Figure S3B). Next, we tested whether ER-stressed macrophages have a specific defect in efferocytosis or a general defect in phagocytosis. In this context, vehicle and 7-KC–exposed macrophages were incubated with either necrotic cells, IgG-conjugated ACs, bacteria (*Escherichia* *coli*), or latex beads (4 µm), representing diverse phagocytic targets that are engulfed via distinct receptor-mediated mechanisms. Similar to the impairment in clearance of ACs, ER-stressed macrophages were also defective in the clearance of necrotic cells and IgG-coated ACs (Figure 3A and 3B). In contrast, the clearance of bacteria and 4 µm latex beads was unaffected in 7-KC–exposed macrophages (Figure 3C and 3D). These findings suggested that ER-stressed macrophages display a selective defect in the engulfment of large-sized particles.

Although the engulfment of small particles by macrophages can be handled by plasma membrane remodeling, intriguingly, the uptake of large particles requires extensive mobilization of endomembranes, which get trafficked to the developing phagosome via focal exocytosis, wherein they fuse and promote phagosome expansion and phagosome closure.^32,42–44^ Because 7-KC–treated macrophages demonstrated defective large particle uptake, we hypothesized that ER-stressed macrophages could have impaired focal exocytosis, resulting in delayed phagosome closure which could manifest as defective efferocytosis. To test this hypothesis, vehicle and 7-KC exposed macrophages were incubated with ACs that were dual-labeled with a fluorophore (green) and coupled with cell surface protein biotinylation. After washing away the unengulfed cells and fixation, macrophages were incubated with streptavidin-fluorophore conjugate (red) with the prediction that streptavidin could access biotinylated AC if they were in a partially sealed phagosome and therefore would fluoresce both green and red (Figure 3E, schematic). In contrast, streptavidin will be unable to bind AC-biotin if they are contained within a sealed phagosome, and therefore, these cells will only fluoresce green.^36^ Consistent with our hypothesis, compared with control macrophages, 7-KC–exposed macrophages had a higher proportion of green+red dual-labeled ACs (Figure 3E), suggesting that these ACs were contained within unsealed/partially sealed phagosomes. Importantly, this defect could be reversed by relieving ER stress via coadministration of 4-PBA (Figure 3E).

To directly test whether the impaired phagosome closure in 7-KC–exposed macrophages stems from defective trafficking of endomembranes, we conducted an assay that quantifies focal exocytosis as demonstrated by us previously.^36^ FM1-43, an amphiphilic styryl dye which fluoresces on binding to membranes, was used to label macrophage endomembranes, followed by incubation with pHrodo-labeled ACs. Because FM1-43 rapidly departs from membranes in an aqueous environment,^45^ the trafficking of endomembranes and its subsequent fusion to AC-phagosome was quantified as a loss of fluorescence intensity by conducting live cell confocal microscopy imaging (Figure 3F, schematic). As expected for a large particle uptake such as ACs, control macrophages demonstrated efficient focal exocytosis of endomembranes as seen by the decrease in FM1-43 signal over time after initiating contact with an AC (Figure 3F). It is important to note that macrophages that were not engulfing an AC did not show a decrease in FM1-43 signal, suggesting that the decrease in fluorescence intensity in efferocytosing macrophages is not due to photobleaching (Figure 3F). In contrast to control macrophages, 7-KC–treated macrophages engulfing ACs showed minimal loss of FM1-43 signal, demonstrating severe defects in focal exocytosis (Figure 3F). Interestingly, this defect can be reversed by treatment with 4-PBA (Figure 3F). Notably, macrophages exposed to palmitate or oxLDL (oxidized low-density lipoprotein) showed similar impairments in phagosome sealing and focal exocytosis (Figure S3C and S3D). These data taken together suggest that lipid-induced ER stress impairs exocytosis of endomembranes toward developing phagosomes, potentially leading to impairment in macrophage efferocytosis.

### ER Stress Impairs Focal Exocytosis via Activation of ATF4-TRIB3-Rab27a Pathway

Based on the data presented above, we next examined the potential molecular mechanism by which ER stress impairs focal exocytosis and promotes defective efferocytosis. In this context, we first tested whether ER stress affects the levels of known exocytosis genes such as *Rab27a*, *Vamp2*, *Snap25*, and *Neurod1*. We observed that 7-KC-exposed macrophages demonstrated a decrease in levels of *Rab27a*, which encodes a GTPase that localizes to vesicles and is critical for their transport to the plasma membrane (Figure S3E).^46^ In contrast, the RNA levels of other key regulators of exocytosis, such as *Vamp2*, *Snap25*, and *Neurod1*, were unaffected (Figure S3E). Interestingly, *Atf4* and *Trib3* (regulated by ATF4) are among the genes that are highly upregulated on cholesterol loading of macrophages^47,48^ and are reported to negatively regulate Rab27a expression and exocytosis in secretory cells.^49^ In this context, we tested whether the ATF4-TRIB3-Rab27a pathway is operative in ER-stressed macrophages and is causally involved in mediating the impairment in focal exocytosis and efferocytosis.

As shown in Figure 1A, ATF4 abundance in macrophages is increased by 7-KC and palmitate in vitro, as well as in atherosclerotic plaque macrophages in vivo.^15^ We, therefore, examined whether ATF4 activation is causally involved in ER stress–induced defective efferocytosis. To address this question, 7-KC macrophages were pretreated with ISRIB (integrated stress response inhibitor), which inhibits PERK-mediated activation of ATF4,^50^ and then incubated with ACs for the efferocytosis assay. ISRIB reversed the 7-KC–induced defect in efferocytosis (Figure 3G). Importantly, this effect was specific to inhibition of the ATF4 branch of the ER stress pathway, as neither blocking IRE1 RNase activity with 4µ8C nor blocking ATF6 with CeapinA7 restored efferocytosis (Figure 3G). Furthermore, the effect of ISRIB was not due to off-target activity, because siRNA-mediated knockdown of *Atf4* similarly reversed the 7-KC-induced efferocytosis impairment (Figure 3H).

To confirm the causal role of the ATF4 branch of the ER stress pathway in impairing macrophage efferocytosis in vivo, we turned to the mouse model of obesity, which displays lipid accumulation, activation of ER stress, and defective efferocytosis in peritoneal macrophages as compared with lean mice. Consistent with the in vitro data, macrophage-specific knockdown of *Atf4* using in vivo jetPEI-Man ^51,52^ in obese mice (Figure S3F) led to an improvement in efferocytosis as quantified by the uptake of fluorescently labeled ACs (Figure 3I).

**Figure 4.**
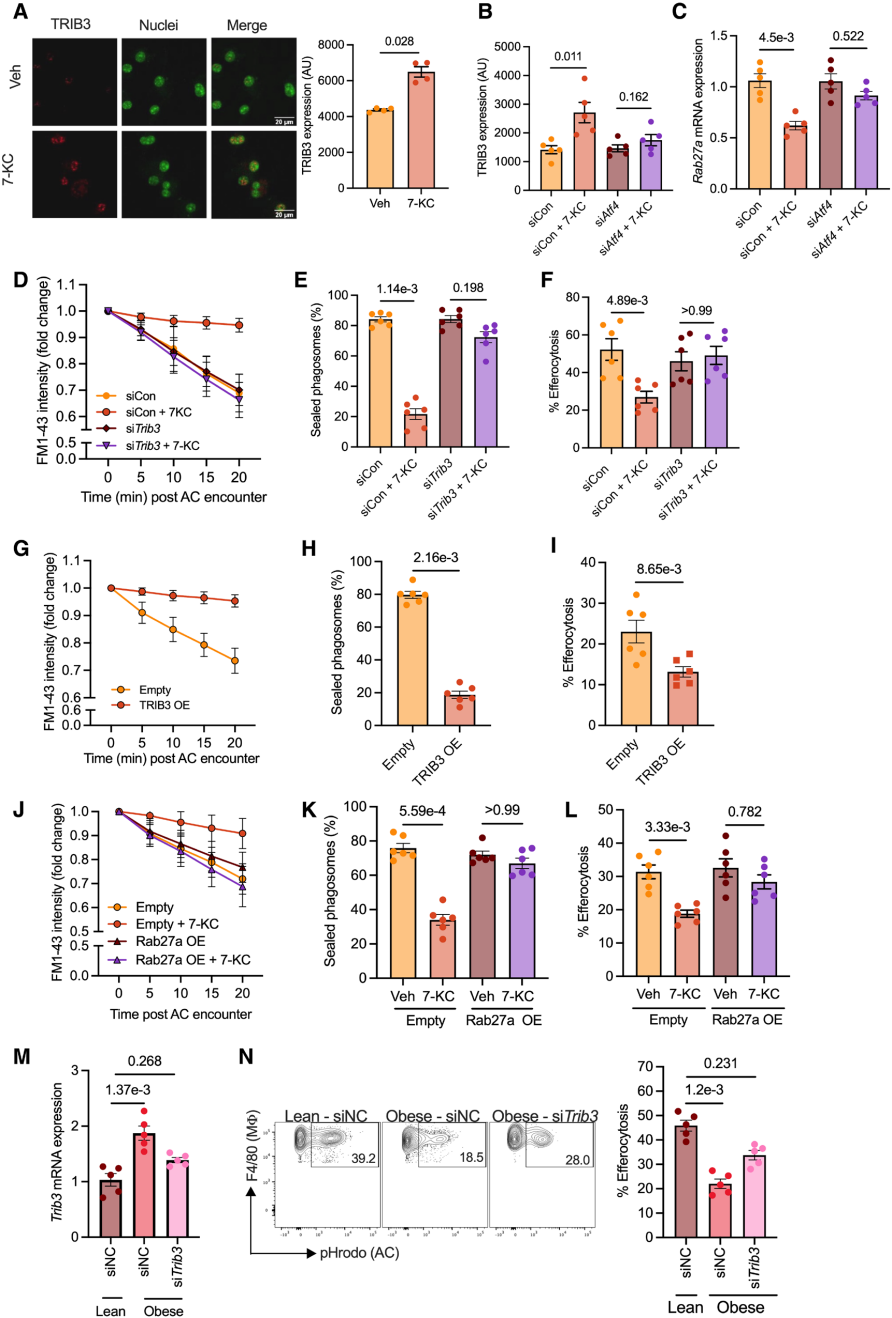
**TRIB3 (Tribbles pseudokinase-3)–Rab27a signaling mediates endoplasmic reticulum stress–induced impairment in macrophage (Mϕ) efferocytosis. A**, Bone marrow–derived macrophages (BMDMs) were exposed to vehicle (Veh) or 7-ketocholesterol (7-KC) for 18 hours, fixed and permeabilized, and immunostained with anti-TRIB3 antibody followed by confocal microscopy. **Left**, Representative images from a single identical z-plane are shown with TRIB3 pseudocolored red and DAPI (4′,6-diamidino-2-phenylindole)–stained nuclei pseudocolored green. Scale bar, 20 µm. **Right**, Quantification of TRIB3 signal intensity within the nuclear plane of Mϕs treated with either Veh or 7-KC. **B**, Mϕs were transfected with either negative control siRNA (siCon) or *Atf4* siRNA, and 48 hours posttransfection, were exposed to Veh or 7-KC for 18 hours. After fixation and permeabilization, TRIB3 levels were quantified by immunostaining and confocal microscopic analysis. **C**, RT-qPCR (real-time quantitative polymerase chain reaction) for quantification of *Rab27a* in Mϕs transfected with negative control siRNA or *Atf4* siRNA and treated with Veh or 7-KC for 18 hours. 18s was used as a housekeeping gene. **D** through **F**, Mϕs transfected with negative control siRNA or *Trib3* siRNA were exposed to either Veh or 7-KC, followed by analysis of (**D**) the kinetics of focal exocytosis of endomembranes towards apoptotic cells (ACs), (**E**) efficiency of phagosome sealing, and (**F**) efferocytosis efficiency. **G** through **I**, Mϕs were nucleofected with either TRIB3 encoding plasmid or empty vector, followed by incubation with ACs for analysis of (**G**) efficiency of focal exocytosis, (**H**) phagosome sealing, and (**I**) efferocytosis. **I** and **J**, Mϕs were nucleofected with Rab27a encoding plasmid or empty vector and exposed to either Veh or 7-KC followed by incubation with ACs for analysis of efficiency of (**J**) focal exocytosis, (**K**) phagosome sealing, and (**L**) efferocytosis. n=4 to 6 biological replicates as indicated. **M** and **N**, Lean and obese mice peritoneal Mϕs were transfected with either negative control siRNA or Trib3 siRNA as indicated, followed by injection of fluorescently labeled ACs. One hour later, the lavage fluid was collected for (**M**) qPCR-based measurement of *Trib3* gene expression in plate-adherent Mϕ, and (**N**) quantification of Mϕ efferocytosis efficiency. n=5 mice per group. The data were analyzed for statistical significance using the Mann-Whitney *U* test (**A**, **H**, and **I**) or Kruskal-Wallis with Dunn multiple comparisons test (**B**, **C**, **E**, **F**, **K** through **N**). AU indicates arbitrary units; OE, overexpression; SiNC, negative control siRNA; SiRNA, silencing RNA; and siTrib3, TRIB3 siRNA.

Next, we tested whether the ATF4-mediated blockade of efferocytosis relied on the activity of the TRIB3-Rab27a pathway. In support of this hypothesis, the decrease in *Rab27a* mRNA levels in 7-KC–treated macrophages was associated with an increase in expression of *Trib3* mRNA and protein (Figure S4A and Figure 4A, respectively). Importantly, knockdown of ATF4 in 7-KC macrophages abrogated both the increase in expression of *Trib3* (Figure 4B; Figure S4B) and the decrease in expression of *Rab27a* (Figure 4C), suggesting that ATF4 activation is upstream of TRIB3 and *Rab27a*. To directly test the role of increased expression of TRIB3 on macrophage efferocytosis, we conducted siRNA-mediated knockdown of *Trib3* in 7-KC macrophages (Figure S4C). Indeed, *Trib3* knockdown in 7-KC macrophages reversed the defect in focal exocytosis of endomembranes toward ACs (Figure 4D; Figure S4D), normalized phagosome sealing (Figure 4E), and reversed the defect in efferocytosis (Figure 4F). It is important to note that *Trib3* knockdown macrophages exposed to 7-KC expressed ATF4 at a level similar to 7-KC macrophages transfected with negative control siRNA (Figure S4E), whereas the expression of Rab27a was maintained at a level similar to control macrophages (Figure S4F), suggesting that TRIB3 is downstream of ATF4 but upstream of Rab27a. Notably, both palmitate and oxLDL induced a similar upregulation of TRIB3 while simultaneously downregulating Rab27a (Figure S4G). This effect was associated with impaired efferocytosis, which could be restored through siRNA-mediated TRIB3 knockdown (Figure S4H). These findings suggest that TRIB3 upregulation is a general mechanism by which atherogenic lipids impair macrophage efferocytosis.

Next, we tested whether artificially increasing the expression of TRIB3 in the absence of ER stress would result in defective efferocytosis. Indeed, increasing TRIB3 levels in macrophages via transfection of a plasmid encoding murine TRIB3 (Figure S5A) led to a decrease in the expression of *Rab27a* (Figure S5B), which was associated with decreased focal exocytosis (Figure 4G), decreased phagosome sealing (Figure 4H), and decreased efferocytosis (Figure 4I). Together, these data implicate a key role for upregulation of TRIB3 in mediating defective macrophage efferocytosis during ER stress.

To test whether the decrease in Rab27a in ER-stressed macrophages is the downstream mediator of defective efferocytosis, we blocked the 7-KC–induced decrease in Rab27a levels by transiently transfecting macrophages with a plasmid encoding murine Rab27a (Figure S5C). The increased expression of Rab27a in this system did not affect the 7-KC–mediated upregulation of TRIB3 (Figure S5D). Consistent with our hypothesis, Rab27a-expressing macrophages were resistant to the 7-KC–induced defect in efferocytosis and displayed efficient phagosome sealing and focal exocytosis (Figure 4J through 4L). Conversely, siRNA-mediated knockdown of *Rab27a* in control macrophages, which mimics the 7-KC–induced decrease in Rab27a expression (Figure S5E), led to defective efferocytosis (Figure S5F) and was associated with decreased phagosome sealing (Figure S5G) and impaired focal exocytosis (Figure S5H). We next examined whether the previously described crosstalk between the IRE1-XBP1s and ATF4 pathways^53^ contributes to the regulation of the TRIB3-Rab27a axis. To test this, 7-KC–exposed macrophages were incubated with the IRE1 inhibitor 4µ8C, and levels of *Trib3* and *Rab27a* were assessed. Unlike the ATF4 inhibitor ISRIB, which suppressed the 7-KC–induced changes in TRIB3 and *Rab27a* (Figure 4B and 4C), 4µ8C had no effect (Figure S5I), suggesting that PERK-ATF4 is a key regulator of the TRIB3-Rab27a axis.

Finally, we leveraged the mouse model of obesity described above to test whether the TRIB3-Rab27a pathway is causative in mediating defective efferocytosis in vivo under conditions of macrophage ER stress. Toward this end, we conducted macrophage-specific knockdown of *Trib3* in peritoneal cavity macrophages and tested their efferocytosis efficiency by quantifying the uptake of fluorescently labeled ACs. First, we noted that *Trib3* knockdown in obese mouse macrophages (Figure 4M) was associated with an increase in *Rab27a* (Figure S5J). More importantly, the suppression of TRIB3 and the increase in Rab27a led to an increase in macrophage efferocytosis efficiency (Figure 4N). These data taken together establish a signaling cascade involving lipid accumulation-induced activation of ATF4, leading to increased expression of TRIB3, which downregulates Rab27a, thereby impairing focal exocytosis of endomembranes towards AC-containing phagosomes with consequent effects on phagosome sealing and AC engulfment.

### Macrophage-TRIB3 Expression Is Higher in Vulnerable Atherosclerotic Plaques of Humans and Is Associated With Impaired Efferocytosis

**Figure 5.**
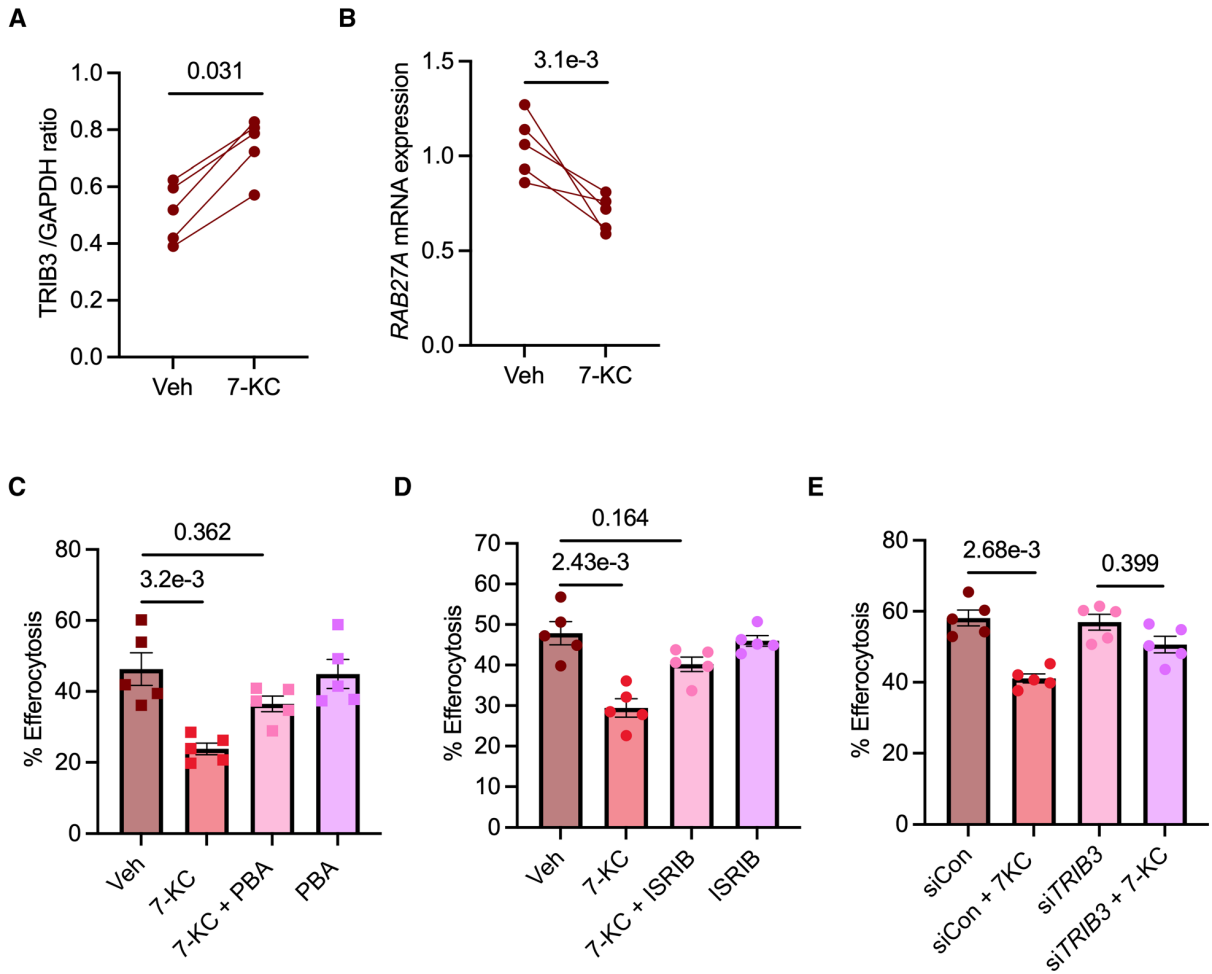
**Lipid-loaded human macrophages (Mϕs) demonstrate ATF4 (activating transcription factor 4)/TRIB3 (Tribbles pseudokinase-3)–mediated impairment in efferocytosis. A**, Human peripheral blood monocyte–derived Mϕs (hMDMs) were treated with vehicle (Veh) or 15 µmol/L 7-ketocholesterol (7-KC) for 18 hours, followed by immunoblotting for quantification of TRIB3 levels. n=5. **B**, RT-qPCR (real-time quantitative polymerase chain reaction) analysis of the *RAB27A* gene in hMDMs treated with Veh or 15 µmol/L 7-KC for 18 hours. 18s was used as the housekeeping gene. n=5. **C** and **D**, hMDMs were exposed to Veh or 7-KC in the absence or presence of 4-phenylbutyric acid (4-PBA; 1 mmol/L) for 18 hours, followed by incubation with pHrodo-labeled apoptotic cells (ACs) at a Mϕ:AC ratio of 1:3 for 1 hour. Efferocytosis efficiency was quantified by fluorescence microscopic analysis. **E**, hMDMs were transfected with either negative control siRNA (siCon) or TRIB3 siRNA (*siTRIB3*). Forty-eight hours posttransfection, Mϕs were incubated with pHrodo-labeled ACs for 1 hour. n=3 to 5 biological replicates as indicated, with averages from technical duplicates. The data were analyzed for statistical significance using a Wilcoxon matched-pairs signed rank test (**A** and **B**) or a Kruskal-Wallis with Dunn multiple comparisons test (**C** through **E**). ISRIB indicates integrated stress response inhibitor; and SiRNA, silencing RNA.

Based on the above data from murine macrophages, we questioned whether human macrophages experiencing lipid-induced ER stress showed similar activation of signaling pathways and defects in efferocytosis. Indeed, human monocyte-derived macrophages exposed to 7-KC showed upregulation of TRIB3 (Figure 5A), which was associated with decreased expression of *RAB27A* (Figure 5B). Furthermore, 7-KC decreased the efferocytosis efficiency of human monocyte-derived macrophages, which could be reversed by blocking ER stress with 4-PBA (Figure 5C). As with murine macrophages, chemical inhibition of ATF4 activation using ISRIB (Figure 5D) or siRNA-mediated knockdown of TRIB3 (Figure 5E) blocked the ER stress–induced defect in efferocytosis. Taken together, these data show that lipid-induced ER stress triggers the ATF4-TRIB3 pathway to impair efferocytosis in human macrophages.

**Figure 6.**
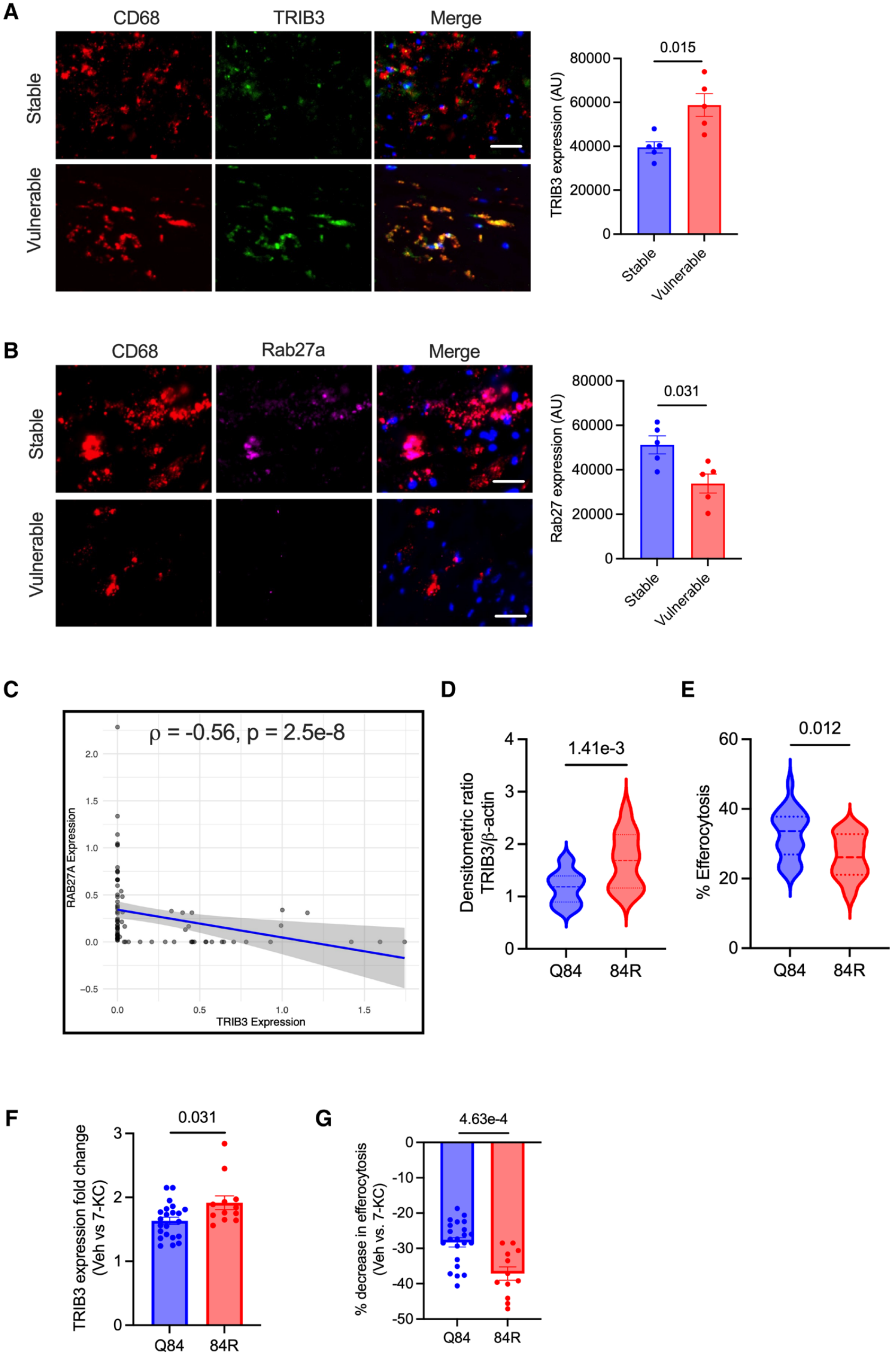
**TRIB3 (Tribbles pseudokinase-3) Q84R single-nucleotide polymorphism (SNP) is associated with impaired macrophage (Mϕ) efferocytosis. A**, Human carotid endarterectomy specimens classified as stable or vulnerable regions were stained with anti-TRIB3 antibody for immunofluorescence microscopy. The sections were costained for CD68 (Mϕ marker) and DAPI (4′,6-diamidino-2-phenylindole). The integrated fluorescence intensity of TRIB3 in CD68+ regions was quantified. n=5. **B**, Similar to **A**, except that Rab27a levels in the CD68+ regions were measured. **C**, Correlation plot between expression of TRIB3 and RAB27A in atherosclerotic plaque Mϕs from scRNA sequencing data set (GSE260657). **D**, Human PBMCs (n=34) were genotyped for rs2295490 by polymerase chain reaction-RFLP and were categorized as carriers of the wild-type (WT) allele (AA, Q84) or variant allele (AG or GG, 84R). Immunoblotting was conducted on whole cell lysates of human monocyte-derived macrophages (hMDMs) for quantification of TRIB3 levels in Q84 and 84R Mϕs. β-actin was used as a loading control. **E**, hMDMs were incubated with pHrodo-labeled apoptotic cells (ACs) for 1 hour, and efferocytosis was quantified by fluorescence microscopic analysis. The efferocytosis efficiency was compared between Q84 and 84R Mϕs. **F**, Q84 and 84R Mϕs were exposed to vehicle (Veh) or 15 µmol/L 7-ketocholesterol (7-KC) for 18 hours before analysis of TRIB3 expression by Western blotting, or (**G**) incubated with pHrodo-labeled ACs for quantification of efferocytosis efficiency. n=22 for WT allele and 12 for the Q84R variant allele. All data are represented as mean±SEM. The data were analyzed for statistical significance using a Mann-Whitney *U* test (**A** and **B**), Spearman correlation coefficient (**C**), or an unpaired *t* test (**D** through **G**). AU indicates arbitrary units; CD, cluster of differentiation; PBMC, peripheral blood mononuclear cells; RFLP, restriction fragment length polymorphism; and ScRNA, single cell RNA.

Because ER stress and ATF4 activation increase with atherosclerotic lesion progression, we questioned whether this is associated with increased expression of TRIB3 in lesional macrophages. Indeed, vulnerable regions of human carotid atherosclerotic plaques showed higher levels of TRIB3 expression (Figure 6A) and lower levels of RAB27 in lesional macrophages as compared with stable regions (Figure 6B). Consistent with these immunohistochemistry results, analysis of a single-cell RNA sequencing data set of human carotid atherosclerotic plaques (GSE260657) revealed that plaque macrophages with higher TRIB3 expression exhibited lower RAB27A levels (Figure 6C), supporting the regulation of RAB27A by TRIB3 observed in our in vitro and murine in vivo studies. Similar to human plaque data, aortic root atherosclerotic plaques from *Apoe*^*−/−*^ mice fed a western diet for 10 weeks exhibited higher TRIB3 expression in lesional macrophages compared with plaques from mice fed the diet for 6 weeks, indicating that TRIB3 expression increases with plaque progression (Figure S6).

Interestingly, previous studies have shown that people with a Q84R gain-of-function variant of TRIB3 (rs2295490-G) have increased carotid intima media thickness and that this variant is associated with early onset myocardial infarction,^54–57^ which, in view of the data reported here, suggests that increased TRIB3 expression could accelerate atherosclerosis progression and exacerbate vulnerable plaque formation. Thus, we hypothesized that individuals with the Q84R single-nucleotide polymorphism (SNP) could have higher levels of cellular TRIB3 expression and, in turn, impaired macrophage efferocytosis. We genotyped PBMCs from 34 individuals (Table S1) for the rs2295490 SNP and simultaneously assayed the expression level of TRIB3 and efferocytosis efficiency in monocyte-derived macrophages. Consistent with previous literature,^49^ we observed that macrophages isolated from individuals displaying the 84R SNP had higher levels of TRIB3 as compared with Q84 macrophages (Figure 6D). Most importantly, macrophages with the 84R SNP had lower efferocytosis efficiency as compared with Q84 macrophages (Figure 6E). Interestingly, when these cells were exposed to 7-KC, the 84R macrophages had a greater increase in TRIB3 expression as compared with Q84 macrophages (Figure 6F). Notably, the increased TRIB3 expression in the 7-KC–treated 84R macrophages was associated with further worsening of efferocytosis as compared with vehicle-treated 84R macrophages (Figure 6G). Collectively, these data suggest that an increase in expression of TRIB3 expression, mediated either by activation of the ER stress signaling pathway or via genetic factors, such as the Q84R SNP, impairs the efferocytosis efficiency of macrophages.

### Hematopoietic Cell–Specific Knockout of *Trib3* Increases Lesional Macrophage Efferocytosis Efficiency and Decreases Atherosclerotic Plaque Necrosis

**Figure 7.**
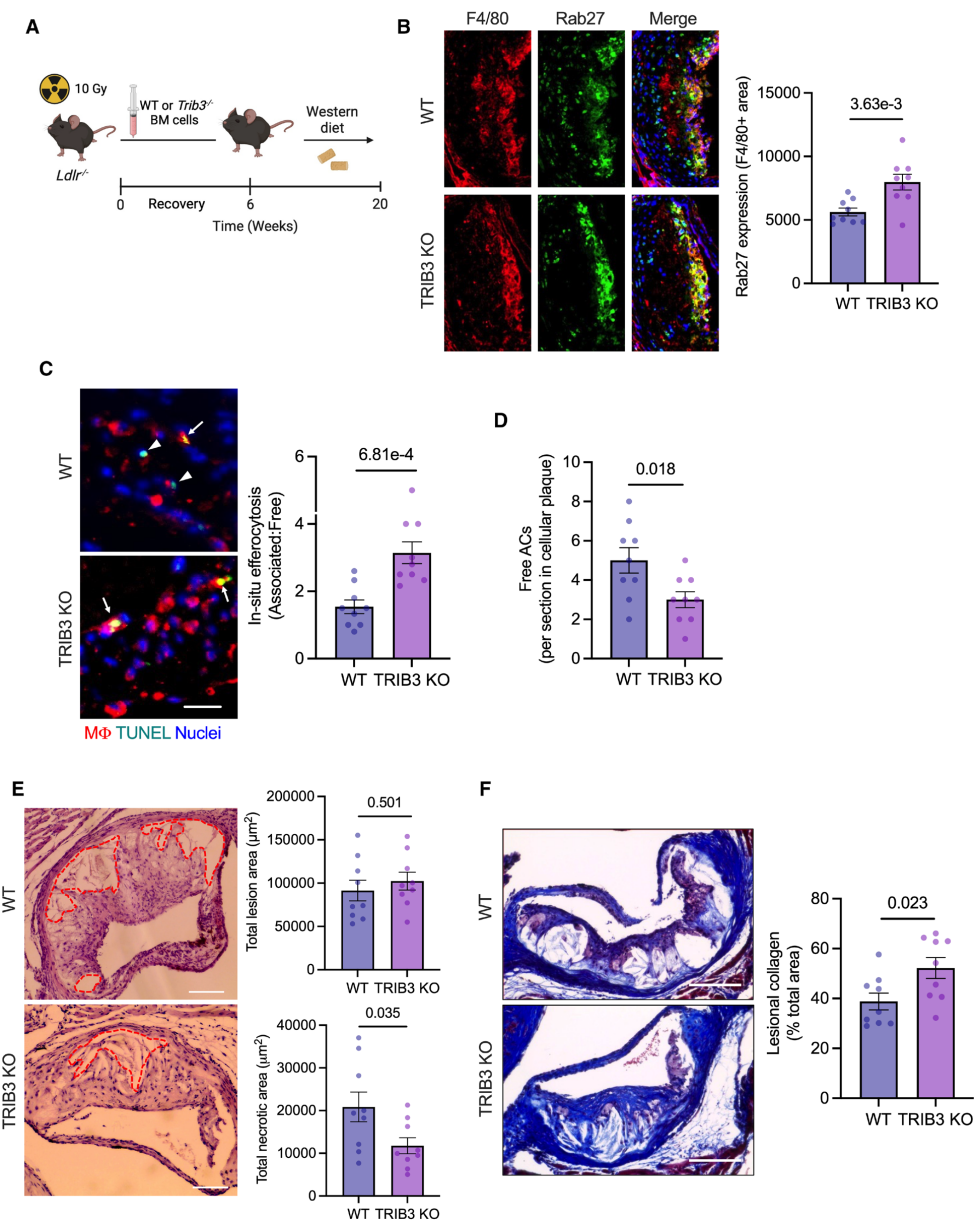
**Hematopoietic deficiency of TRIB3 (Tribbles pseudokinase-3) leads to improved lesional macrophage (Mϕ) efferocytosis and decreased plaque necrosis. A**, *Ldlr*^*−/−*^ mice were X-ray irradiated, followed by reconstitution with either wild-type (WT) or TRIB3 knockout (KO) bone marrow (BM) cells to generate chimeric mice that are deficient in TRIB3 in hematopoietic cells. Six weeks post–BM transplantation, the mice were fed a Western-type diet for 14 weeks, followed by euthanasia and analysis of aortic atherosclerosis. **B**, Aortic root sections of WT and TRIB3 KO mice were stained with anti-F/80 (red) and anti-Rab27 (green) Ab for immunofluorescence microscopy. Representative images and quantification of Rab27 intensity in F4/80+ regions of the plaque are shown. Scale bar, 100 µm. **C**, Aortic root sections of WT and TRIB3 KO mice were labeled with TUNEL (green) and anti-F4/80 Ab (red) for fluorescence microscopy. White arrows show TUNEL+ apoptotic cells (ACs) contained within an F4/80+ Mϕ, whereas the arrow heads show ACs that are lying free. The bar graph shows the ratio of TUNEL+ cells that are associated with an F4/80+ Mϕ *vs* free-living TUNEL+ cells. Scale bar, 50 µm. **D**, Quantification of non-Mϕ–associated free TUNEL+ ACs in the cellular regions of the plaque in WT and TRIB3 KO mice. n=9 mice per group. **E**, Representative images and quantification of total lesion area and plaque necrosis in hematoxylin and eosin–stained aortic root sections of WT and TRIB3 KO mice. The broken red line marks acellular regions characteristic of plaque necrosis. **F**, Representative images from WT and TRIB3 KO aortic root sections stained with Masson trichrome staining for detection of collagen. The bar graph presents quantification of intimal collagen+ area as a percent of total lesion area. n=9 mice per group. All data are represented as mean±SEM. The data were analyzed for statistical significance using an unpaired Student *t* test. Ab indicates antibody; and TUNEL, terminal deoxynucleotidyl transferase dUTP nick end labeling.

The progression of atherosclerotic plaque towards a clinically dangerous rupture-prone vulnerable plaque phenotype is driven partly by impairment in lesional macrophage efferocytosis. Because both human and murine plaque macrophages experience chronic ER stress with disease progression and demonstrate increased TRIB3 expression, particularly in unstable regions of the plaque (Figure 6A), we hypothesized based on the data presented above that ER stress–mediated increased expression of TRIB3 in macrophages could represent a mechanistic link between atherosclerotic plaque progression and the associated impairments in lesional macrophage efferocytosis efficiency. To test this hypothesis, we transplanted bone marrows derived from either wild-type or *Trib3*^*−/−*^ mice into lethally irradiated *Ldlr*^*−/−*^ mice to generate bone marrow chimeric mice that are deficient in TRIB3 in all hematopoietic-derived cells, including atherosclerotic plaque macrophages. The chimeric mice were fed a high-fat, high-cholesterol Western-type diet for 14 weeks to induce advanced atherosclerotic plaques (Figure 7A). First, there were no statistically significant differences in body weight, blood glucose, total plasma cholesterol, plasma triglycerides, and peripheral blood cell counts between the 2 groups of mice (Figure S7A through S7E), indicating that loss of TRIB3 in hematopoietic cells does not result in major metabolic or cellular perturbations under conditions of atherogenic dyslipidemia. Moreover, there was no difference in the efficiency of oxLDL uptake between wild-type and TRIB3 knockdown macrophages (Figure S7F). We confirmed that TRIB3 expression was indeed lost in the lesional macrophages of TRIB3 knockout chimeric mice (TRIB3 knockout; Figure S7G). Consistent with our in vitro data, the loss of TRIB3 expression was associated with an increase in expression of Rab27 in lesional macrophages (Figure 7B), demonstrating that the ER stress–induced TRIB3-Rab27a signaling axis was operational in this pathological setting. Next, we tested whether the increased expression of Rab27a was associated with improvement in lesional macrophage efferocytosis efficiency by conducting an in situ efferocytosis assay in which ACs within the plaque were identified by fluorescent labeling with TUNEL (terminal deoxynucleotidyl transferase dUTP nick end labeling) reagent and queried whether these ACs were associated with a F4/80+ macrophage (efferocytosed) or were lying free. The ratio of macrophage-associated: free ACs is an indicator of lesional efferocytosis efficiency.^58^ Consistent with our hypothesis, TRIB3 knockout mice showed an increase in the ratio of macrophage-associated: free ACs as compared with control mice (Figure 7C). Moreover, the number of free ACs in the plaque was decreased in the TRIB3 knockout mice (Figure 7D), indicating improved lesional macrophage efferocytosis efficiency. Analysis of aortic root sections demonstrated that the total lesion area was similar between the 2 groups of mice (Figure 7E), which is consistent with the lack of systemic changes in metabolic profile. Although the lesion area was unaltered, plaque necrosis, which is a critical determinant of plaque vulnerability in humans, was significantly decreased in the TRIB3 knockout mice (Figure 7E). Because plaque necrosis is primarily driven by defective efferocytosis, the reduction in free ACs and lesional necrosis, together with the comparable numbers of plaque macrophages in TRIB3 knockout and control mice (Figure S7H), is consistent with enhanced efferocytic efficiency of lesional macrophages in the TRIB3 knockout mice. Notably, TRIB3 knockdown macrophages in vitro were equally susceptible to cell death induced by ER stress (Figure S7I), suggesting that the decrease in atherosclerotic plaque necrosis in TRIB3 knockout mice is driven primarily by increased lesional macrophage efferocytosis. Finally, TRIB3 knockout mice showed increased lesional collagen deposition as compared with control mice (Figure 7F). These data, coupled with improved efferocytosis and decreased plaque necrosis, demonstrate that suppression of TRIB3 results in the development of a stable plaque phenotype.

## Discussion

Cholesterol accumulation in lesional cells induces chronic ER stress, leading to inflammation and cell death with consequent exacerbation of plaque necrosis and thinning of the fibrous cap, processes that result in the formation of a rupture-prone vulnerable plaque.^21^ Besides the role of ER stress in inducing apoptosis and inflammation, we present new evidence demonstrating that chronic adaptive ER stress directly impairs the efferocytosis efficiency of macrophages during obesity and atherosclerosis. Mechanistically, we show that lipid accumulation in macrophages activates an ATF4-TRIB3 signaling axis, which decreases Rab27a levels, a key protein involved in promoting exocytosis. This decrease in Rab27a blocks the exocytic trafficking of endomembranes towards the AC-containing nascent phagosome, leading to defective phagosome closure with consequent impairment in engulfment of ACs. In addition, we provide evidence that a common SNP in TRIB3 (Q84R) in humans is associated with impairment in macrophage efferocytosis efficiency at baseline, which is further exacerbated under conditions of atherogenic dyslipidemia. Finally, using a preclinical mouse model of advanced atherosclerosis, we show that targeting TRIB3 in atheroma could be an attractive therapeutic strategy to improve lesional macrophage efferocytosis efficiency, decrease plaque necrosis, and promote atherosclerotic plaque stabilization.

The generation of foamy macrophages is a key initiator and driver of atherosclerosis. Interestingly, the accumulated lipid triggers ER stress and is implicated in exacerbating inflammatory responses and in the induction of lesional cell death,^59^ processes that are intricately linked to atherosclerotic plaque progression. Recent studies in dyslipidemic and nondyslipidemic contexts have suggested that macrophage ER stress could lead to impairment in efferocytosis efficiency.^18,22,23^ These reports used a model of acute severe ER stress, which is often associated with perturbation of macrophage function and initiation of apoptosis signaling pathways, which could directly impair the ability of macrophages to perform efferocytosis due to compromised cellular health. To circumvent these limitations, our study used a model of adaptive ER stress wherein UPR signaling pathways are activated but do not lead to macrophage death, thereby more closely mimicking the pathophysiological state of lesional macrophages in advanced atherosclerotic plaques. Moreover, our study demonstrates that molecular pathways identified using this in vitro system, such as the upregulation of TRIB3 and downregulation of Rab27a, are observed in both mouse and human atherosclerotic plaques, establishing the translational relevance of this model for conducting future studies exploring molecular mechanisms and novel drug development and testing.

During phagocytosis, the ER directly contributes membrane to the developing phagosome in a process called ER-mediated phagocytosis.^44^ Under conditions of ER stress, wherein ER homeostasis is perturbed, it is conceivable that ER membrane trafficking to the AC-phagosome could be impaired, accounting for the delayed phagosome closure. Although, our data shows a general defect in exocytic trafficking of endomembranes towards the developing phagosome in ER-stressed macrophages, whether this is mediated in part due to impairment in ER-mediated phagocytosis remains to be tested. Recent data demonstrated that acute ER stress in macrophages activates IRE1-mediated phosphorylation of FMRP (fragile X mental retardation protein) which downregulates the expression of efferocytosis receptors Mertk and LRP-1 in vitro in macrophages and is associated with defects in continuous efferocytosis.^18^ In addition, inhibition of IRE1 activity or the deletion of FMRP in macrophages was associated with reversal of defective efferocytosis in vitro and in vivo in mouse models of advanced atherosclerosis.^18^ In contrast, we did not observe alterations in cell surface levels of Mertk and LRP-1 in our model of chronic ER stress. It is conceivable that depending on the duration and signal intensity of specific stressors, ER stress could activate multiple signaling cascades with significant crosstalk^53^ that affect macrophage efferocytosis either directly or indirectly.

SNPs in TRIB3, particularly the common Q84R variant (rs2295490-G), wherein the glutamine (Q) is substituted with Arginine (R) at position 84, is associated with increased risk of developing type 2 diabetes, increased carotid intima media thickness, and adverse vascular complications including early onset myocardial infarction and stroke.^49,54–57^ Interestingly, the 84R variant is a gain-of-function variant with a prevalence of ≈ 25% to 30% in White^57^ and Asian people^60^ and is associated with increased stability and levels of TRIB3 and increased protein activity.^49^ In vitro studies suggest that the increased activity of TRIB3 in endothelial cells expressing the 84R variant leads to impaired NO production and enhanced expression of VCAM-1 (vascular cell adhesion molecular 1) and ICAM-1 (intercellular adhesion molecular 1), resulting in increased monocyte adhesion^57^ which could be linked to accelerated atherosclerosis. In addition, the 84R variant is associated with insulin resistance, diabetes, and lipid alterations,^49,54–57^ all of which are risk factors for accelerated atherosclerosis progression. However, nondiabetic people with the Q84R variant also demonstrate enhanced adverse CAD outcomes suggesting that TRIB3 could accelerate atherosclerosis progression independent of its metabolic effects.^57^ Interestingly, our data reveal that relative to macrophages from individuals with the TRIB3 Q84, those with the 84R variant contain more TRIB3 and, these have a lower efferocytosis efficiency. Importantly, this difference in efferocytosis is further exacerbated in foamy macrophages suggesting that individuals with the 84R variant could be particularly susceptible to defective macrophage efferocytosis associated with dyslipidemia with consequent exacerbation of plaque necrosis thereby increasing vulnerability of the plaque to rupture and the associated adverse cardiovascular outcomes.

Our previous study^61^ and current data in human atherosclerosis shows that TRIB3 expression in macrophages is higher in vulnerable regions of the plaque as compared with stable regions. Moreover, murine plaques show an increase in TRIB3 levels along with a concomitant decrease in Rab27a as plaques progress, suggesting an association between increased expression of TRIB3 and plaque instability. In this context, we previously reported that TRIB3 holo-knockout mice have increased fibrous cap thickness which was associated with decreased expression of matrix metalloproteinases *Mmp8* and *Mmp12* in TRIB3 knockout macrophages, suggesting that increased expression of TRIB3 could promote plaque instability via triggering the degradation of extracellular matrix.^61^ Similarly, silencing the expression of TRIB3 by siRNA was associated with increased atherosclerotic plaque stability, but only in the diabetic *Apoe*^*−/−*^*Ldlr*^*−/−*^ mice.^62^ Altogether, these findings suggest a critical role for elevated lesional TRIB3 levels with plaque progression and destabilization.

Notably, TRIB3 is expressed by several cells and therefore holo-TRIB3 deficiency could have pleiotropic effects on multiple cell types, including vascular endothelial cells, adipocytes, and pancreatic β cells, all of which could influence the progression of atherosclerosis. To avoid these confounding issues and to specifically examine the role of hematopoietic cell TRIB3 expression on atherosclerotic plaque progression, we developed the TRIB3 knockout bone marrow chimera model in *Ldlr*^*−/−*^ mice. In contrast to the holo-TRIB3 knockout mice, which showed higher adiposity,^61^ hematopoietic TRIB3 knockout mice displayed no differences in body weight and metabolic parameters, thereby eliminating the confounding roles of TRIB3 expression on nonimmune cells to atherosclerotic plaque progression. Also, in vitro studies demonstrated that overexpression of TRIB3 increases foam cell formation on exposure to oxLDL in the human THP1 macrophage cell line.^63^ In contrast to these findings obtained with supraphysiological levels of TRIB3, knockdown of TRIB3 in macrophages did not affect their efficiency of uptake of lipids. Because the total lesion area in the aortic root was similar between wild-type and macrophage-TRIB3–deficient *Ldlr*^*−/−*^ mice, these data suggest that macrophage TRIB3 plays a minimal role in foam cell formation in atherosclerosis.

Furthermore, our data reveal that the loss of TRIB3 expression in atherosclerotic plaque macrophages is associated with increased lesional efferocytosis and decreased plaque necrosis. Importantly, the increased efferocytosis efficiency in the macrophage-TRIB3 deficient mice was associated with an increase in expression of Rab27 in lesional macrophages, suggesting that the ATF4/TRIB3/Rab27 axis that we discovered using in vitro studies is relevant and operative in vivo in the context of advanced atherosclerosis. Taken together, our studies demonstrate a causal role for ER stress–triggered increase in expression of TRIB3 in mediating defective macrophage efferocytosis and increased plaque necrosis, thereby promoting plaque instability.

Our study has certain limitations. First, although we modeled adaptive ER stress to better mimic the state of lesional macrophages, ER stress is heterogeneous, and other pathways (eg, IRE1-FMRP signaling^18^) may also contribute to impaired efferocytosis depending on the severity of stress experienced by macrophages. Second, our murine atherosclerosis data suggest a key role for the suppression of macrophage-TRIB3 in enhancing efferocytosis and promoting plaque stability; however, it is possible that some of the observed effects may also arise from loss of TRIB3 signaling in nonmacrophage leukocytes, such as T and B cells, which, although expressing only low levels of TRIB3, could still contribute. Finally, the TRIB3 Q84R variant is associated with enhanced insulin resistance,^56^ and the relative contributions of altered glucose homeostasis versus impaired efferocytosis to the accelerated atherosclerosis phenotype in patients remain to be tested.

In summary, our study demonstrates that activation of ER stress signaling directly impairs macrophage efferocytosis efficiency in obesity and atherosclerosis. Given that similar pathways are activated in several chronic metabolic and inflammatory diseases, including chronic obstructive pulmonary disease and inflammatory bowel disease,^64,65^ it is tempting to speculate that ER stress targeting drugs could be an attractive therapeutic strategy to promote efferocytosis and quell inflammation. Indeed, several preclinical studies have shown the therapeutic efficacy of ER stress–relieving agents such as 4-PBA^16,66^ and tauroursodeoxycholate.^67^ However, due to the critical physiological roles of UPR signaling cascades in several tissues and cells, such as the secretory pancreatic β-cells, plasma cells, and dendritic cells, targeting ER stress for the amelioration of disease has been hampered by on-target effects of UPR pathway inhibiton.^68^ In this context, our identification of the TRIB3-Rab27a axis as a downstream effector of ER stress signaling in mediating defective efferocytosis opens new avenues for therapeutic targeting while sparing the adverse consequences elicited by global ER stress relievers. For example, we propose that targeted nanomedicine approaches to silence macrophage TRIB3 expression could be an attractive precision medicine strategy to enhance lesional macrophage efferocytosis and promote atherosclerotic plaque stabilization.

## ARTICLE INFORMATION

### Acknowledgments

Figure schematics were created using Biorender.com.

### Sources of Funding

This work was supported by a Barts Charity grant (MGU0459 and G-002421), British Heart Foundation (BHF) project grant (PG/22/11226), and a UKRI-BBSRC grant (BB/Y513143/1) to M. Subramanian. A. Singhal and U.K. Dhawan were funded by Queen Mary University of London (QMUL) Principal Studentship. K. Bhutia was a recipient of an Marie Sklodowska-Curie Actions Early Stage Researcher (MSC ESR) contract associated with the Horizon 2020 European Training Networks program, grant number H2020-MSCA-ITN-308 2016 721532. T.D. Nightingale was supported by the BHF project grant (PG/22/11208). M. de Gaetano was supported by an Irish Research Council Postdoctoral Fellowship. C. Godson is supported by the Juvenile Diabetes Research Foundation (JDRF) and Science Foundation Ireland. Work in G. Velasco group is supported by the Instituto de Salud Carlos III (ISCIII) and cofounded by the European Regional Development Fund (ERDF), “A way to make Europe,” grant number PI18/00442 integrated into the State Plan for R&D+I 2017-2020 and grant number PI21/00343 integrated into the State Plan for R&D+I 2021-2023, by the European Commission through the Horizon 2020 European Training Networks program, grant number H2020-MSCA-ITN-308 2016 721532 and by the Madrid Region Government Network Program in Biosciences, grant number S2022/BMD-7434 (Advanced Strategies and new Approaches for Protontherapy [ASAP-CM]). H.L. Wilson and E. Kiss-Toth were funded by the European Commission Horizon 2020 Marie Skłodowska-Curie Innovative Training Network, Tribbles Research and Innovation Network (TRAIN) (grant number H2020-MSCA-ITN-308 2016 721532). Spinning disk confocal microscopy at QMUL was supported by a Cancer Research, UK (CRUK) Microscopy Core Service grant at Barts Cancer Institute (Core Award C16420/A18066).

### Disclosures

None.

### Supplemental Material

Supplemental Methods

Table S1

Figures S1–S7

Major Resources Table

ARRIVE Guidelines

Peer Review Report

References 56, 69–72

## Supplementary Material
