# Supplementary material for: TRIB3 Links Endoplasmic Reticulum Stress to Impaired Efferocytosis in Atherosclerosis
Source: Circ Res. 2025 Nov 4;137(12):1422–42. doi: 10.1161/CIRCRESAHA.125.326839 (PMC12680282; doi:10.1161/CIRCRESAHA.125.326839)
Supplement: Supplementary file 1 [file res-137-1422-s001.pdf]

Supplementary Material and Major Resources Table

TRIB3 Links Endoplasmic Reticulum Stress to Impaired Efferocytosis in Atherosclerosis

Aarushi Singhal[1], Stefan Russo[1], Umesh Kumar Dhawan[1], Kunzangla Bhutia[2], Christopher G. Bell[1], Hedayatullah Hayat[1], Thomas D. Nightingale[1], Monica de Gaetano[3], Orina Belton[3], Eoin Brennan[4], Patricia B. Munroe[1], Catherine Godson[4], Mary Barry[5], Carol C Shoulders[1], Heather L. Wilson[6], Guillermo Velasco[2], Endre Kiss-Toth[6], Manikandan Subramanian[1]

Expanded Materials and Methods

Isolation and differentiation of bone marrow-derived macrophages

8-10 weeks old female C57BL/6J mice weeks were utilized to isolate bone marrow cells for Mϕ differentiation, as described previously[69]. The cells were cultured in DMEM High Glucose (Sigma #D5671) with 10% Fetal Bovine Serum, and 20% L-929 cell culture supernatant for 7 days in 10 cm petri dishes. On day 3, half the media was replaced with fresh media. Fully differentiated Mϕs were harvested on day 7 for further experiments.

Generation of human monocyte-derived Mϕs

For functional studies involving human Mϕs, 30 ml of peripheral blood was collected from healthy human volunteers after obtaining informed consent (QMERC2019/83). PBMCs were isolated by Ficoll-Hypaque density gradient centrifugation and monocytes were further enriched by plate adhesion. The adherent monocytes were cultured in the presence of 40 ng/ml recombinant human M-CSF for 7-10 days to allow Mϕ differentiation. For studies involving TRIB3 genotyping and associated functional studies, frozen PBMCs were obtained from a commercial source (BioIVT, London, UK). The cells were from both males and females with ages ranging from 22 – 74 years (**Table S1**). The cells were thawed, and the monocytes were enriched using monocyte isolation kit (Miltenyi Biotech), following manufacturer's protocol. The monocytes were

cultured in the presence of 40 ng/ml recombinant human M-CSF for 10 days to allow Mϕ differentiation.

Fluorescence labelling of cells and induction of apoptosis

Cells were labelled with either pHrodo red, pHrodo deep red, or PKH67 as per manufacturer's protocols. Briefly, for PKH67 labeling, cells were incubated with 2 µM PKH67 in diluent C for 3 min. The reaction was quenched with addition of FBS followed by 3X washes with DMEM-10% FBS.

For pHrodo red (20 ng/ml) and pHrodo deep red (100 ng/ml) labelling, cells were resuspended at a density of $1x10^6$ cells per mL of PBS at pH 8.0 and incubated at 37°C for 30 minutes in the dark. The cells were then washed twice with PBS and resuspended in DMEM-10% FBS. To induce apoptosis, the fluorescently labelled cells were exposed to UV-C (254 nm) for 7 minutes (5 kJ/m^2). After UV treatment, the cells were incubated at 37°C for 1 hour to generate ACs. This method yielded approximately 90% ACs as confirmed using Annexin-V staining.

Annexin-V staining

For annexin-V staining, BMDMs were seeded in 24 well plate at density of $5x10^5$ and stained using the Dead Cell Apoptosis Kit with Annexin-V (Thermo #V13242) as per the manufacturer's protocol. Briefly, 5X annexin binding buffer was diluted to 1X using dH_2O. Cells were incubated with 100 µL 1X buffer along with 10 µL of Annexin-V Alexa Fluor 488 for 20 min at RT. Post incubation, cells were washed with 1X annexin binding buffer and imaged using an EVOS fluorescence microscope. The images were then analyzed using Fiji ImageJ software wherein cells were counted for positive annexin (green) signal.

Oil red O staining of cells

4% paraformaldehyde fixed cells were incubated with 60% isopropanol for 5 min at RT. After removing isopropanol, the cells were air-dried and then incubated with Oil Red O solution (0.3% in isopropanol) at RT for 15 min in the dark on constant shaking. Cells were washed with ddH_2O, and brightfield images were acquired. For quantitative

analysis, the intracellular dye was eluted using 100% isopropanol at RT for 10 min on a shaker and absorbance of the eluate was measured at 500 nm as an indicator of intracellular neutral lipid accumulation.

TRIB3 and Rab27a overexpression

Murine *Trib3* plasmid was obtained from Addgene (Cat# 131157)[70]. 1x10^6 Mϕs were resuspended in 100 µL of nucleofector buffer and mixed with 0.5 µg of TRIB3 or Rab27a plasmid and nucleofected using Y-001 program in the Amaxa Nucleofector II system. The nucleofected cells were used for experiments 48 h post-transfection.

siRNA transfection

Mϕs were transfected with commercially available siRNAs complexed with Lipofectamine® RNAiMAX Reagent (Thermo # 13778100) as per manufacturer's protocol. Briefly, siRNA and lipofectamine were mixed in a 1:1 ratio to yield a final concentration of 10 nM. The siRNA-lipofectamine complex was added to cells placed in Opti-MEM and incubated for 6 h followed by addition of complete growth medium. Gene knockdown was validated 48 h post-transfection using either qRT-PCR or western blotting.

RNA isolation, cDNA synthesis, and qPCR

For total RNA extraction, RNeasy Mini Kit (Qiagen) was utilized as per the manufacturer's protocol. Further, 1 µg of total RNA was converted into cDNA using PrimeScript 1st strand cDNA Synthesis Kit (Takara). To study gene expression, qRT-PCR was conducted wherein the synthesized cDNA was combined with KAPA SYBR Green master mix, serving as a fluorescent reporter, along with gene-specific primers. Gene-specific primer pairs listed in Major Resource Table were used for amplification of target genes. For normalization, 18s RNA was employed as the housekeeping gene. Roche LightCycler 480 system was utilized for qRT-PCR. Relative gene expression was determined through the ddCt method.

Western Blotting

Whole-cell lysates were generated by lysis in 2X Laemmli buffer and 30 μl of protein/well was loaded onto 10% SDS-PAGE gel. After electrophoresis, the resolved proteins were transferred to PVDF membrane (Millipore, USA). The membranes were blocked with 5% BSA for 1 h followed by incubation with antibodies against ATF4, TRIB3, XBP1, or β-actin for 16 h at 4°C with constant gentle shaking. After 3 washes with TBST, the membranes were incubated with respective HRP-conjugated secondary antibodies (1:5000), and the chemiluminescent signal was captured on an Azure 400 Imager (Azure Biosystems). The images were exported as a TIFF file and densitometric analysis was conducted using ImageJ.

Zymosan-induced peritonitis

16-weeks-old lean or obese mice (fed a high-fat diet for 10 weeks, D12492 Research diets) were administered either vehicle (DMSO in PBS) or 4-PBA (40 mg/kg) intraperitoneally on two consecutive days prior to administration of zymosan (1 mg) intraperitoneally. Appropriate groups of mice were euthanized at 0 h, 6 h, 12 h, 18 h, 24 h, and 48 h post zymosan injection and peritoneal lavage was conducted. The cells in exudates were used for analysis of the numbers of neutrophils, macrophages, and in-situ efferocytosis by flow cytometry. Characterization of neutrophils and Mϕs was performed by immunostaining with anti-Ly6G and anti-F4/80 antibody respectively. Apoptotic neutrophils were analyzed by annexin-V FITC staining on Ly6G-gated cells. In situ efferocytosis efficiency was quantified in permeabilized cells stained for F4/80 and Ly6G. Efferocytosis event was defined as LyG+F4/80+ cells. Non-permeabilized cells stained with Ly6G and F4/80 were used to set up appropriate positive gates. The exudate supernatant was used for analysis of TNF (Invitrogen) and LXA4 (Cayman Chemical) by ELISA.

Metabolic profiling of mice

For glucose testing, the mice were fasted for 5 hours. The glucose levels were then evaluated using glucose strips and a glucometer manufactured by Accuchek. To assess plasma cholesterol, mice were fasted for 12 h. Cholesterol was measured using the cholesterol assay kit (RANDOX-CH 200) and triglyceride levels were measured using the RANDOX- TR 210 kit, as per manufacturer's protocol.

Aortic Root Atherosclerotic Lesion Analysis

The mice were euthanized, followed by intraventricular perfusion of the heart with 10 mL 1X PBS. The heart and aortic root were harvested and fixed in 10% neutral buffered formalin. Fixed heart tissues were embedded in paraffin and 8 μm thickness sections were cut using a microtome. Fifty serial sections were taken from the first appearance of the aortic valve. H&E staining was conducted on six plaque sections evenly distributed throughout the aortic root to interpret lesion size and necrotic core area. Sections were stained with Mason Trichrome dye (Sigma-Aldrich) according to the manufacturer's instructions to examine lesional collagen expression.

For immunofluorescence, antigen retrieval was performed following deparaffinization using either Proteinase-k (20 μg/mL), for TRIB3 and Mac2, at 37°C for 20 minutes or sodium citrate buffer (10 mmol/L Sodium citrate, 0.05% Tween-20, pH 6.0), for sm-actin and F4/80, at 95°C for 45 minutes. The sections were then rinsed for 5 minutes with 1X PBS. Following washing, blocking was performed for 30 minutes at room temperature with PBS containing 3% FBS. The sections were subsequently treated overnight at 4°C with anti-F4/80 (1:100), anti-TRIB3 (1:100), anti-Mac2 (1:100), and anti-SM-actin (1:100), anti-Rab27a (1:25) primary antibodies. Following incubation with the primary antibody of interest, the sections were washed three times with 1X PBS and treated with fluorescently labelled secondary antibodies (1:1000) and DAPI. Following 1 h, the sections were washed once again with 1X PBS and imaged under a fluorescence microscope (EVOS FL2). Fiji was used to perform the image analysis (ImageJ). The fluorescence intensity of the positive signal for the respective protein was calculated by marking the area and comparison was done between the groups.

In-situ efferocytosis assay

For In-situ efferocytosis quantification, deparaffinized lesional sections were stained for apoptotic cells using the Click-iT Plus TUNEL Assay Kit (Thermo #C10619) according to the manufacturer's instructions. Following TUNEL labelling of apoptotic cells, the sections were immunostained with anti-F4/80 antibody to mark lesional Mϕs. Images were captured using a fluorescence microscope (EVOS FL2). Fiji (ImageJ) was used to determine the ratio of TUNEL+ apoptotic cells that were associated with F4/80+

macrophages vs free lying TUNEL+ apoptotic cells within the cellular regions of the plaque.

TRIB3 genotyping

Genotyping was performed as described previously[56]. Briefly, PCR was performed using specific primers (Major Resource Table) followed by restriction digestion with MspI. The products were electrophoresed on a 2% agarose gel and visualized with SYBR Safe. The AA genotype yielded a single 593 bp band; AG genotype yielded a 593 bp and a 297 bp bands; while the GG genotype yielded a single 297 bp band.

Single cell RNA sequencing analysis

Publicly available single-cell RNA sequencing data from human carotid atherosclerotic plaques (GSE260657)[71] were analyzed using the Seurat pipeline. Quality control was performed as described earlier[72], by excluding cells with (i) fewer than 300 detected genes, (ii) total gene counts outside the range of 50,000–750,000, or (iii) >10% mitochondrial gene content. Data were then normalized, variable features identified, and scaling performed. Unsupervised clustering with UMAP dimensionality reduction identified 14 clusters (0–13). Macrophage-enriched clusters were defined by canonical markers (CD68, CD14, APOE). Cluster 11 was selected for further analysis, as it showed the strongest enrichment for plaque-associated gene signatures (TREM2, LGALS3, FABP4, SPP1) and an expanded plaque marker set (TREM2, LGALS3, FABP4, CHI3L1, SPP1, MMP9). After excluding cells with zero expression of RAB27A and TRIB3, expression levels of these genes were quantified within the cluster.

Figure S1

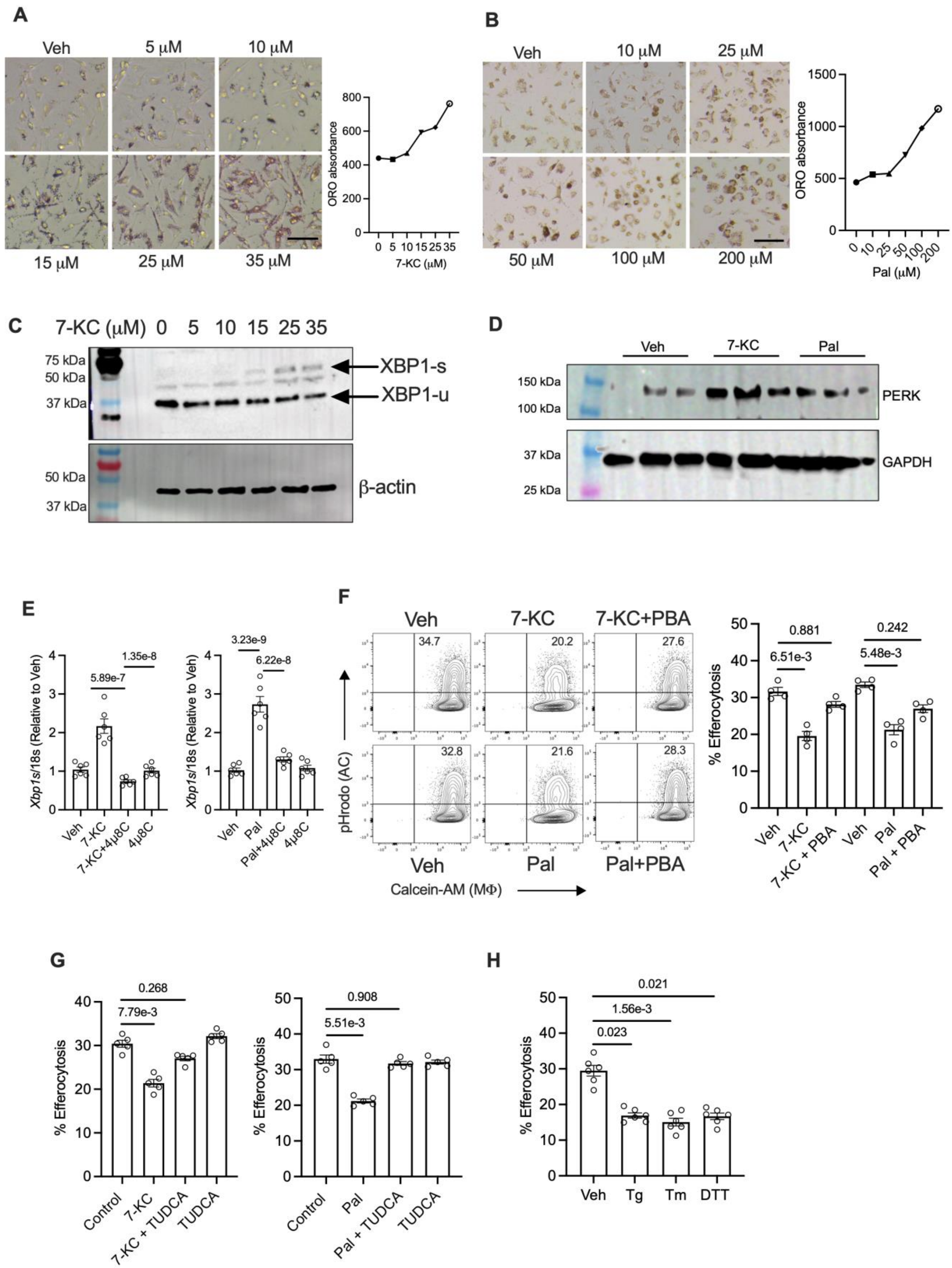

A
Veh
5 μM
10 μM
15 μM
25 μM
35 μM
ORO absorbance
7-KC (μM)
B
Veh
10 μM
25 μM
50 μM
100 μM
200 μM
ORO absorbance
Pal (μM)
C
7-KC (μM) 0 5 10 15 25 35
75 kDa
50 kDa
37 kDa
XBP1-s
XBP1-u
50 kDa
37 kDa
β-actin
D
Veh
7-KC
Pal
150 kDa
100 kDa
PERK
37 kDa
25 kDa
GAPDH
E
Xbp1s/18s (Relative to Veh)
5.89e-7
1.35e-8
Veh
7-KC
7-KC+4μ8C
4μ8C
3.23e-9
6.22e-8
Veh
Pal
Pal+4μ8C
4μ8C
F
Veh
7-KC
7-KC+PBA
34.7
20.2
27.6
32.8
21.6
28.3
Veh
Pal
Pal+PBA
pHrodo (AC)
Calcein-AM (MΦ)
% Efferocytosis
0.881
6.51e-3
0.242
5.48e-3
Veh
7-KC
7-KC + PBA
Veh
Pal
Pal + PBA
G
% Efferocytosis
0.268
7.79e-3
Control
7-KC
7-KC + TUDCA
TUDCA
0.908
5.51e-3
Control
Pal
Pal + TUDCA
TUDCA
H
% Efferocytosis
0.021
1.56e-3
0.023
Veh
Tg
Tm
DTT

Figure S1. Establishment of a model of chronic adaptive ER stress in Mϕs. (A and B) Representative images of oil red O (ORO) staining in BMDMs treated with indicated concentrations of 7-KC (A) or Pal (B) for 18 h. The line graph shows quantification of ORO by measurement of absorbance at 500 nm. (C) Western blotting for detection of spliced form of XBP1 (XPB1-s) in whole cell lysates of Mϕs treated with indicated concentrations of 7-KC for 18 h. β-actin was used as loading control. (D) Western blotting for detection of PERK in whole cell lysates of Mϕs treated with either vehicle (Veh), 15 µM 7-KC, or 50 µM palmitate for 18 h. GAPDH was used as loading control. (E) Mϕs were exposed for 18 h to either vehicle, 15 µM 7-KC, or 50 µM palmitate as indicated in the absence or presence of 1 µM 4µ8C. RT-qPCR was performed to quantify levels of spliced XPB-1. (F) Vehicle or 7-KC exposed calcein-AM labeled Mϕs were incubated with pHrodo-labeled ACs followed by quantification of efferocytosis efficiency by flow cytometry. n = 4 biological replicates. (G) Efferocytosis assay with pHrodo-labeled ACs in BMDMs treated with either 7-KC or palmitate for 18 h in the absence or presence of TUDCA (200 µM). n = 5 biological replicates. (H) Efferocytosis assay with pHrodo-labeled ACs was conducted in BMDMs treated with either tunicamycin (Tn, 2 µg/ml), thapsigargin (Tg, 62.5 nM), or DTT (1 mM). n = 3 biological replicates. The data were analyzed for statistical significance usingKruskal-Wallis with Dunn's multiple comparisons test (E-H).

Figure S2

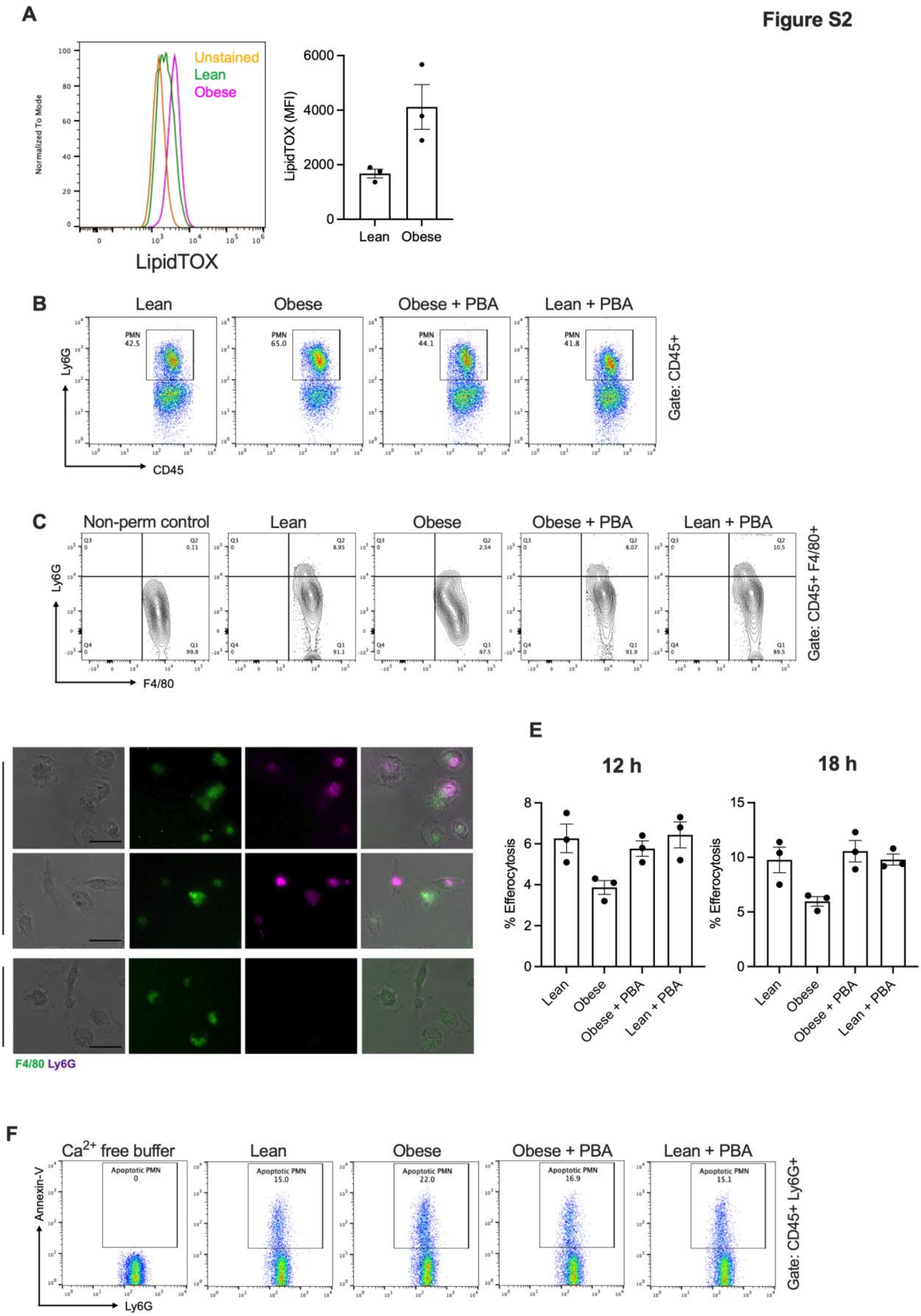
A
Unstained
Lean
Obese
Normalized To Mode
LipidTOX
LipidTOX (MFI)
Lean
Obese
B
Lean
Obese
Obese + PBA
Lean + PBA
PMN
42.5
65.0
44.1
41.8
Ly6G
CD45
Gate: CD45+
C
Non-perm control
Lean
Obese
Obese + PBA
Lean + PBA
Ly6G
F4/80
Gate: CD45+ F4/80+
D
F4/80+ Ly6G+
F4/80+ Ly6Gneg
F4/80 Ly6G
E
12 h
18 h
% Efferocytosis
Lean
Obese
Obese + PBA
Lean + PBA
F
Ca2+ free buffer
Lean
Obese
Obese + PBA
Lean + PBA
Apoptotic PMN
0
15.0
22.0
16.9
15.1
Annexin-V
Ly6G
Gate: CD45+ Ly6G+

Figure S2. ER stress in obese mice impairs Mϕ efferocytosis and delays of resolution of zymosan-induced peritonitis. (A) Peritoneal Mϕs from lean and obese mice were stained with LipidTOX and fluorescence intensity was analyzed by flow cytometry. n = 3 mice per group. (B) Flow cytometric dot plots representing quantification of neutrophil (CD45+ Ly6G+) numbers in peritoneal lavage of indicated groups of mice at 18 h post injection of zymosan. (C) Representative flow cytometric contour plots of quantification of efferocytosis efficiency in peritoneal lavage cells stained with anti-F4/80 antibody followed by membrane permeabilization and staining with anti-Ly6G antibody. The "Non-perm control" represents anti-F4/80 and anti-Ly6G antibody staining in cells that were not subjected to membrane permeabilization. The plots display events within the CD45+ F4/80+ gate. (D) Representative fluorescence microscopy images of FACS sorted F4/80+Ly6G+ cells and F4/80+Ly6GNeg cells obtained from peritoneal lavage of lean mice after 24 h of intraperitoneal administration of 1 mg zymosan. F4/80, green; Ly6G, magenta; Bar, 10 μm. (E) Flow cytometric analysis of macrophage efferocytosis efficiency in appropriate groups of mice at 12 h and 18 h post zymosan injection. (F) Representative flow cytometric dot plots of Annexin-V staining in peritoneal lavage cells from appropriate groups of mice 18 h post injection of zymosan. Cells stained with Annexin-V FITC in the absence of Ca^{2+} (Ca^{2+} free buffer) were used as negative control.

Figure S3

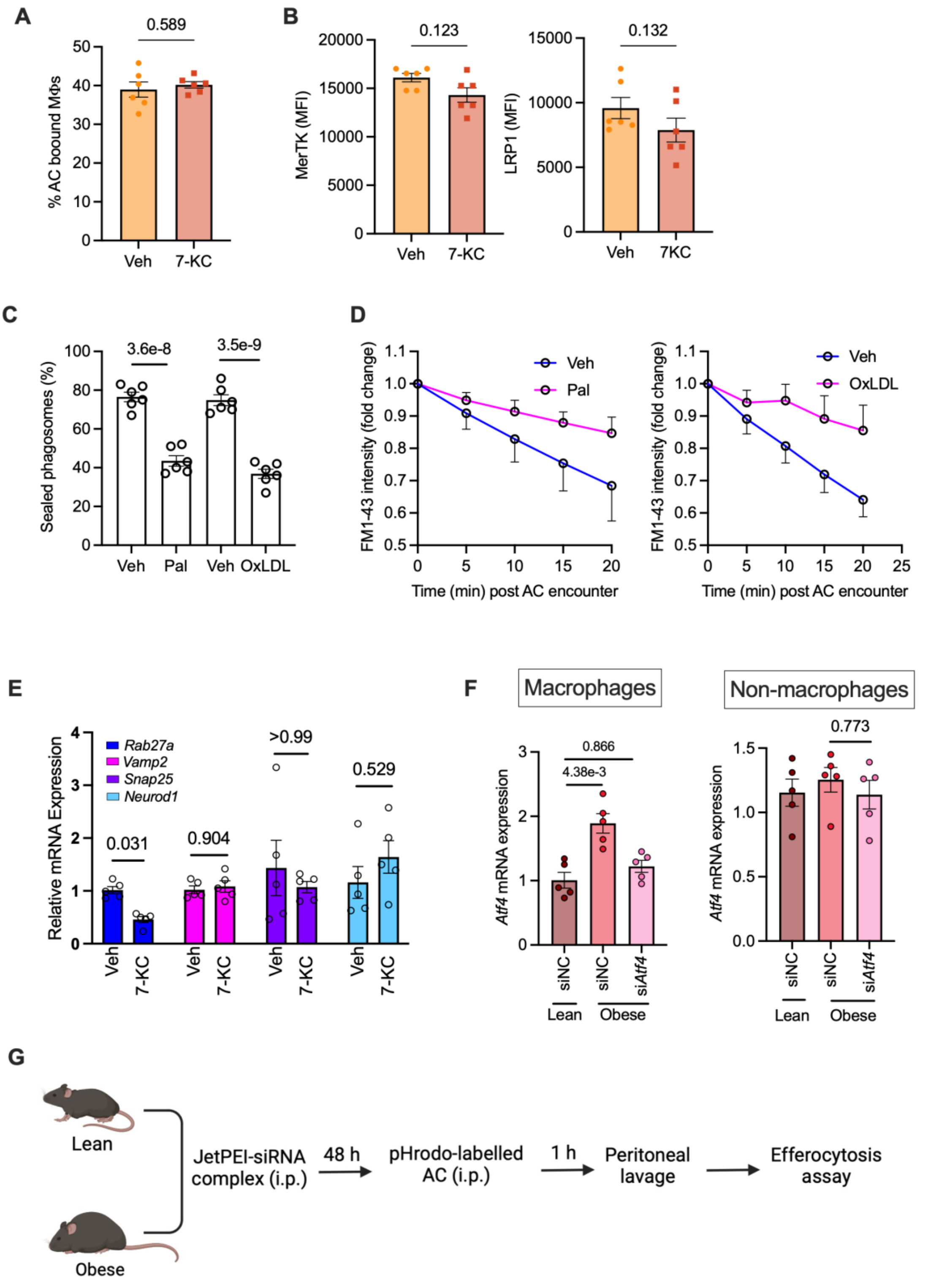
A
0.589
% AC boound MΦs
Veh
7-KC
B
0.123
MerTK (MFI)
Veh
7-KC
0.132
LRP1 (MFI)
Veh
7KC
C
3.6e-8
3.5e-9
Sealed phagosomes (%)
Veh
Pal
Veh
OxLDL
D
Veh
Pal
FM1-43 intensity (fold change)
Time (min) post AC encounter
Veh
OxLDL
FM1-43 intensity (fold change)
Time (min) post AC encounter
E
Rab27a
Vamp2
Snap25
Neurod1
Relative mRNA Expression
0.031
0.904
>0.99
0.529
Veh
7-KC
F
Macrophages
Non-macrophages
0.866
4.38e-3
Atf4 mRNA expression
siNC
siNC
siAtf4
Lean
Obese
0.773
Atf4 mRNA expression
siNC
siNC
siAtf4
Lean
Obese
G
Lean
Obese
JetPEI-siRNA complex (i.p.)
48 h
pHrodo-labelled AC (i.p.)
1 h
Peritoneal lavage
Efferocytosis assay

Figure S3. (A) Vehicle and 7-KC exposed Mφs were incubated with CytochalasinD (1 μM) for 30 min followed by addition of fluorescently labeled ACs. The percent macrophages bound to an AC were quantified by fluorescence microscopy. n = 6 biological replicates. (B) Flow cytometry based quantification of cell surface levels of Mertk and LRP-1 in Mφs exposed to vehicle or 7-KC. (C) Mφs exposed to either vehicle, palmitate, or OxLDL was assessed for (C) phagosome sealing efficiency and (D) focal exocytosis. (E) RT-qPCR for analysis of gene expression changes of *Rab27a*, *Vamp2*, *Snap25*, and *Neurod1* in BMDMs exposed to vehicle or 15 μM 7-KC for 18 h. 18s was used as housekeeping gene. The values are expressed as fold change relative to vehicle-treated cells. n = 3 biological replicates. (F) qPCR-based quantification of *Atf4* levels in plate-adherent peritoneal Mφs and non-Mφs from indicated groups of mice. n = 5 mice per group. (G) Schematic representation of *in vivo* siRNA transfection of peritoneal cavity macrophages in lean and obese mice followed by efferocytosis analysis. The data are represented as mean ± SEM.The data were analyzed for statistical significance using Mann Whitney (A, B) or Kruskal-Wallis with Dunn's multiple comparisons test (C, E, F).

Figure S4

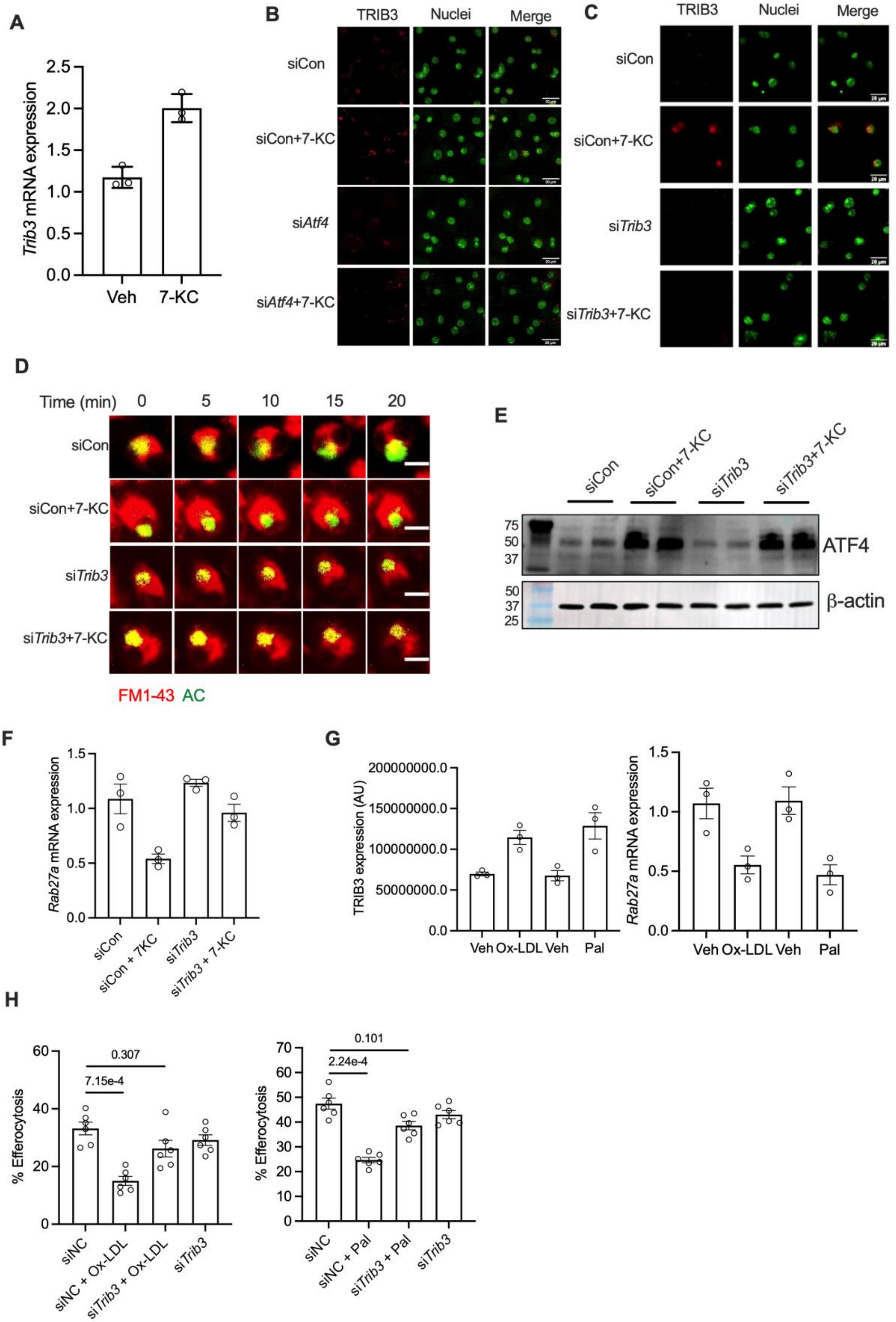
A
Trib3 mRNA expression
Veh
7-KC
B
TRIB3
Nuclei
Merge
siCon
siCon+7-KC
siAtf4
siAtf4+7-KC
C
TRIB3
Nuclei
Merge
siCon
siCon+7-KC
siTrib3
siTrib3+7-KC
D
Time (min)
0
5
10
15
20
siCon
siCon+7-KC
siTrib3
siTrib3+7-KC
FM1-43 AC
E
siCon
siCon+7-KC
siTrib3
siTrib3+7-KC
ATF4
β-actin
F
Rab27a mRNA expression
siCon
siCon + 7KC
siTrib3
siTrib3 + 7-KC
G
TRIB3 expression (AU)
Veh
Ox-LDL
Veh
Pal
Rab27a mRNA expression
H
% Efferocytosis
0.307
7.15e-4
siNC
siNC + Ox-LDL
siTrib3 + Ox-LDL
siTrib3
0.101
2.24e-4
siNC + Pal
siTrib3 + Pal

Figure S4. Activation of ATF4-TRIB3 axis leads to defective focal exocytosis and efferocytosis in Mϕs. (A) RT-qPCR-based analysis of *Trib3* gene expression in BMDMs treated with either vehicle or 15 µM 7-KC for 18 h. n = 3 biological replicates. (B) Representative images of TRIB3 immunostaining (red) in BMDMs transfected with negative control siRNA (siCon) or *Atf4* siRNA (si*Atf4*) and exposed to either vehicle or 15 µM 7-KC for 18 h. Nuclei were stained with DAPI (pseudo colored green). Scale bar, 20 µm. (C) Similar to B, except that Mϕs were transfected with negative control siRNA or *Trib3* siRNA followed by incubation with vehicle or 7-KC for 18 h. (D) Representative images of live cell confocal microscopy from appropriate groups of Mϕs showing FM1-43 staining (red) in Mϕs captured every 5 minutes for 20 minutes from the time of contact with an AC (green). (E) Immunoblotting for quantification of ATF4 expression in BMDMs transfected with negative control siRNA or *Trib3* siRNA followed by incubation with either vehicle or 15 µM 7-KC for 18 h. β-actin was used as loading control. (F) RT-qPCR for quantification of *Rab27a* gene expression in BMDMs transfected with negative control siRNA or *Trib3* siRNA and incubated with vehicle or 15 µM 7-KC for 18 h. 18s was used as housekeeping gene and the data are plotted as fold change relative to control Mϕs. n = 3 biological replicates. (G) Analysis of TRIB3 levels (left panel) and Rab27a levels (right panel) in BMDMs exposed to Ox-LDL or Pal for 18 h. n = 3 biological replicates. (H) BMDMs transfected with either negative control siRNA or Trib3 siRNA were exposed to vehicle or Ox-LDL as indicated followed by incubation with pHrodo-labeled ACs for quantification of efferocytosis. n = 6 biological replicates. All data are represented as mean ± SEM. The data were analyzed for statistical significance using Kruskal-Wallis with Dunn's multiple comparisons test (H).

Figure S5

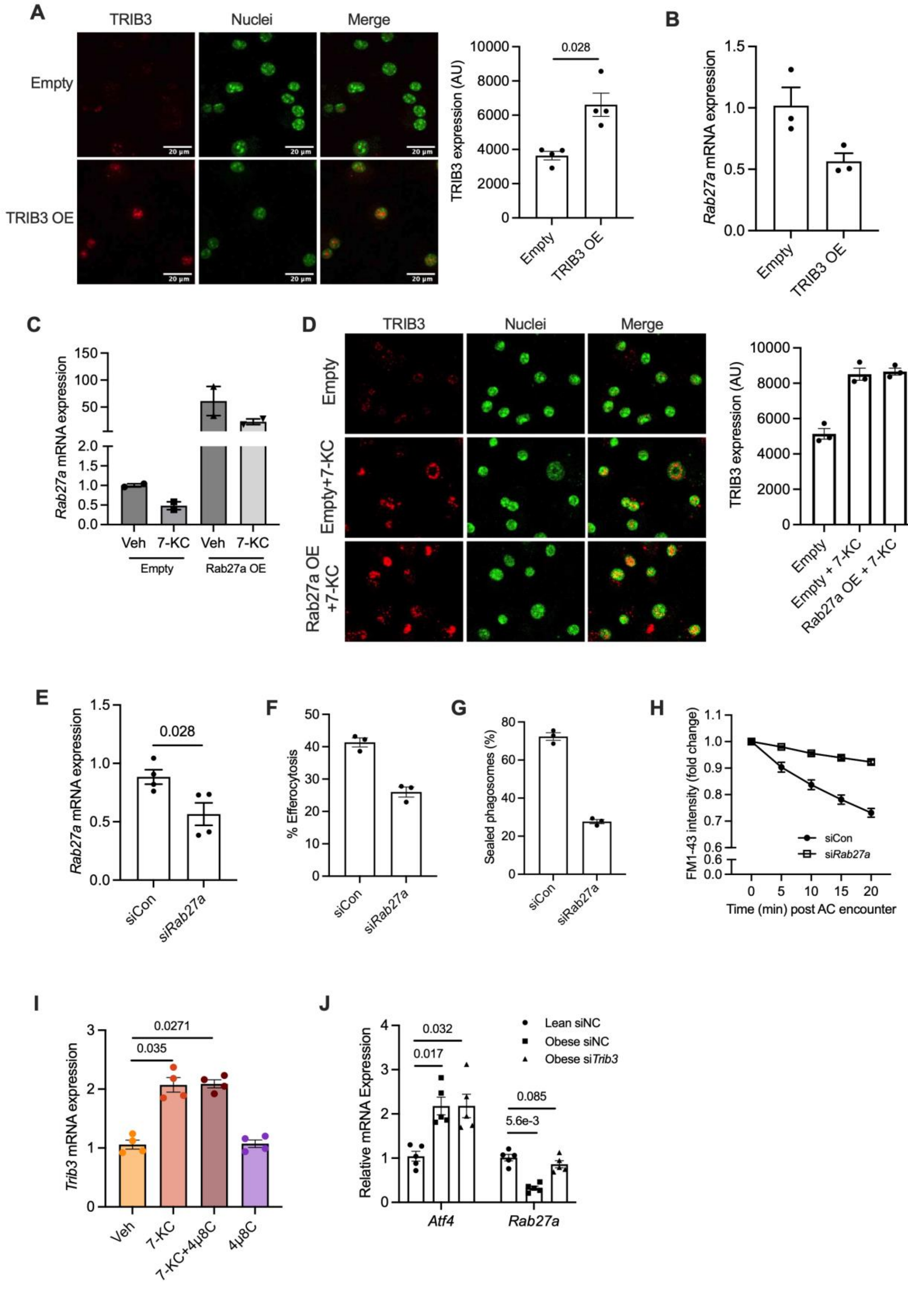

A
TRIB3
Nuclei
Merge
Empty
TRIB3 OE
20 µm
TRIB3 expression (AU)
0.028
B
Rab27a mRNA expression
C
Rab27a mRNA expression
Veh
7-KC
Empty
Rab27a OE
D
Empty+7-KC
Rab27a OE +7-KC
Empty + 7-KC
Rab27a OE + 7-KC
E
siCon
siRab27a
F
% Efferocytosis
G
Sealed phagosomes (%)
H
FM1-43 intensity (fold change)
Time (min) post AC encounter
I
Trib3 mRNA expression
0.0271
0.035
7-KC+4µ8C
4µ8C
J
Relative mRNA Expression
0.032
0.017
0.085
5.6e-3
Lean siNC
Obese siNC
Obese siTrib3
Atf4
Rab27a

Figure S5. TRIB3-mediated downregulation of Rab27a impairs efferocytosis in ER-stressed Mϕs. (A) Representative images from a single z-plane showing expression of TRIB3 in BMDMs nucleofected with either TRIB3-expression plasmid or an empty vector. Bar, 20 µm. The right panel shows quantification of TRIB3 intensity in nucleus of the appropriate groups of cells. (B) RT-qPCR analysis of *Rab27a* mRNA expression in BMDMs nucleofected with TRIB3-plasmid or empty vector. 18s was used as housekeeping gene and the data are plotted as fold change relative to control Mϕs. (C) Similar to B, except that BMDMs were nucleofected with Rab27a-plasmid or empty plasmid and 48 h later were incubated with vehicle or 15 µM 7-KC for 18 h. (D) Quantification of nuclear-localized TRIB3 intensity in BMDMs nucleofected with empty or Rab27a-expressing plasmid. (E) RT-qPCR based analysis of *Rab27a* mRNA expression in BMDMs transfected with negative control siRNA or *Rab27a* siRNA. 18s was used as housekeeping gene and the data are plotted as fold change relative to negative control siRNA transfected Mϕs. (F) BMDMs were transfected with negative control siRNA or *Rab27a* siRNA. 48 h post-transfection, Mϕs were incubated with pHrodo-labeled ACs for 1 h followed by fluorescence microscopy for analysis of efferocytosis efficiency (F); efficiency of phagosome sealing (G); and efficiency of focal exocytosis upon encounter with an AC (H). (I) Analysis of Trib3 mRNA levels in Mϕs exposed to 7-KC in the absence or presence of 4µ8C. (J) RT-qPCR based analysis of *Atf4* and *Rab27a* mRNA levels in peritoneal cavity macrophages of lean and obese mice transfected with either negative control siRNA or *Trib3* siRNA as indicated. The data were analyzed for statistical significance using Kruskal-Wallis test with Dunn's multiple comparison correction (I, J).

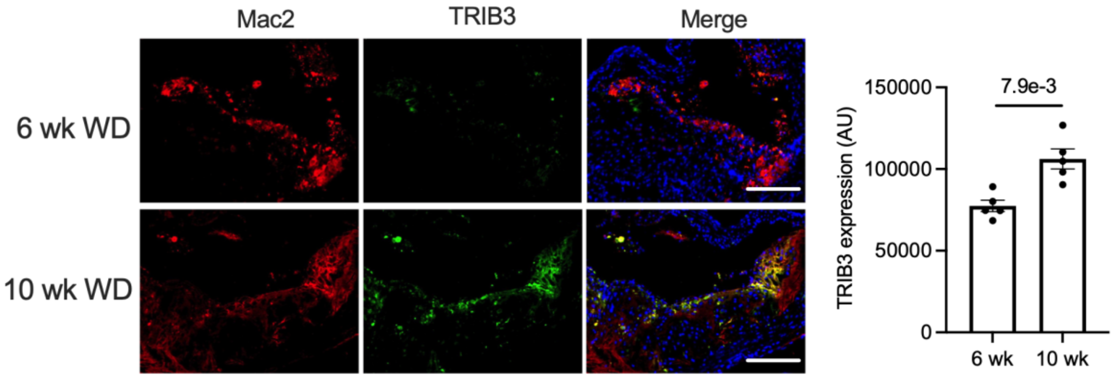

Figure S6. Aortic root sections of *Apoe*$^{-/-}$ mice fed a western type diet for 6 weeks or 12 weeks were stained for TRIB3 (green) and Mϕ marker Mac2 (red) for immunofluorescence microscopy. TRIB3 intensity was quantified in Mac2+ regions of the plaque.The data were analyzed for statistical significance using Mann-Whitney test.

Figure S7

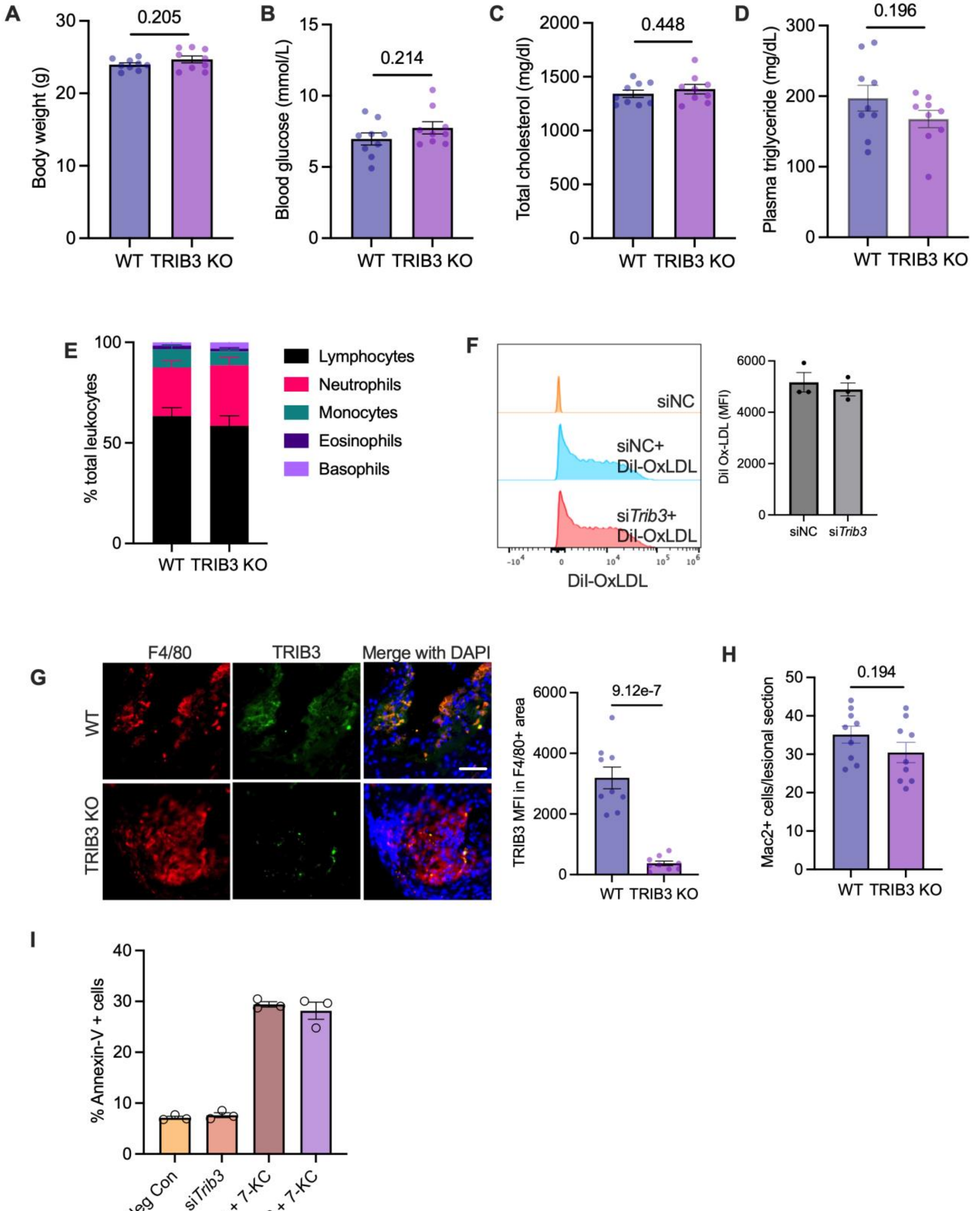

A
Body weight (g)
0.205
WT
TRIB3 KO
B
Blood glucose (mmol/L)
0.214
C
Total cholesterol (mg/dl)
0.448
D
Plasma triglyceride (mg/dL)
0.196
E
% total leukocytes
Lymphocytes
Neutrophils
Monocytes
Eosinophils
Basophils
F
siNC
siNC+
DiI-OxLDL
siTrib3+
DiI-OxLDL
DiI-OxLDL
DiI Ox-LDL (MFI)
siNC
siTrib3
G
F4/80
TRIB3
Merge with DAPI
WT
TRIB3 KO
TRIB3 MFI in F4/80+ area
9.12e-7
H
Mac2+ cells/lesional section
0.194
I
% Annexin-V + cells
siNeg Con
siTrib3
siNeg Con + 7-KC
siTrib3 + 7-KC

Figure S7. Metabolic parameters are unaffected in hematopoietic TRIB3 deficient *Ldlr$^{-/-}$* mice fed a western diet for 14 weeks. (A) Body weight measurement in 14 weeks western type diet-fed WT or TRIB3 KO *Ldlr$^{-/-}$* mice. (B) Blood glucose was measured in the two groups of mice at 13 weeks of WD feeding following a 5 h fasting protocol. (C) Plasma from WT or TRIB3 KO bone marrow chimeric *Ldlr$^{-/-}$* mice fed a WD for 14 weeks were analyzed for total cholesterol levels (C) and triglyceride content (D). (E) Differential blood count analysis between the two groups of mice. (F) WT and *Trib3* knockdown BMDMs were incubated with DiI-labelled Ox-LDL and the uptake efficiency was quantified by measuring the DiI fluorescence intensity by flow cytometry. (G) Immunostaining for TRIB3 in aortic root sections for validation of TRIB3 deficiency in atherosclerotic lesional Mϕs (F4/80+) of WD-fed *Ldlr$^{-/-}$* mouse transplanted with TRIB3 KO bone marrow. (H) Quantification of the number of Mac2+ cells by immunostaining in the aortic root of WT and TRIB3 KO mice. (I) Analysis of percent annexin-V + cells in BMDMs transfected with negative control siRNA or *Trib3* siRNA followed by exposure to vehicle or 15 μM 7-KC for 18 h. All data are represented as mean ± SEM and were analyzed for statistical significance using an unpaired Student's t-test.

Major Resources Table

Animals (in vivo studies)

Mouse strain	Vendor or Source	Background Strain	Sex
C57BL/6J	Charles River, UK	C57BL/6J	F/M
Ldlr$^{-/-}$	The Jackson Laboratory	C57BL/6J	F
Trib3$^{-/-}$	Complutense University and Instituto de Investigación Sanitaria, San Carlos (IDISSC) Madrid, Spain	C57BL/6J	F

Antibodies

Target antigen	Vendor or Source	Catalog #	Working concentration	Persistent ID / URL
Mac-2 (Galectin-3)	Biolegend	125402	10 µg/ml	RRID: AB_1134238
Anti-Actin, α-Smooth Muscle	Sigma	A2547-.2ML	4 µg/ml	RRID: AB_476701
TRIB3	Invitrogen	PA5-114501	20 µg/ml	RRID: AB_2890514
Rab27a	Abcam	ab55667	20 µg/ml	RRID: AB_945112
ATF4	Cell Signaling Technology	11815	1 µg/ml	RRID: AB_2616025
Xbp1s	Abcam	ab37152	1 µg/ml	RRID: AB_778939
Ly6G	Biolegend	126715	10 µg/ml	RRID:AB_1877271
F4/80	Invitrogen	12-4801-82	10 µg/ml	RRID: AB_465923

Cultured Cells

Name	Vendor or Source	Sex (F, M, or unknown)	Persistent ID / URL
L929	ATCC	M	https://www.atcc.org/products/ccl-1

Primer sequences

Mouse: β-actin - Forward	5’ TGTTACCAACTGGGACGACA 3’
Mouse: β-actin - Reverse	5’ GGGGTGTTGAAGGTCTCAAA 3’
Mouse: 18 S - Forward	5’ GTAACCCGTTGAACCCCATT 3’
Mouse: 18 S - Reverse	5’ CCATCCAATCGGTAGTAGCG 3’
Mouse: Trib3 - Forward	5’ TCAAGCTGCGTCGCTTTGTC 3’
Mouse: Trib3 - Reverse	5’ AGCTGAGTATCTCTGGTCCCACGTA 3’
Mouse: Rab27a - Forward	5’ GGCACGTTGGGAATCTAGC 3’
Mouse: Rab27a – Reverse	5’ AGTGTAGCGTCCTTAGCTGG 3’
TRIB3 Exon2 - Forward	5’ GGCCACCAAGCAGTCTCAC 3’
TRIB3 Exon2 - Reverse	5’ CGCCCATGATCCCTAAGTTC 3’
Neurod1 - Forward	5’ CCCTACTCCTACCAGTCCCC 3’
Neurod1 - Reverse	5’ GAGGGGTCCGTCAAAGGAAG 3’
Snap25 - Forward	5’ ATGTTGGATGAGCAAGGCGA 3’
Snap25 - Reverse	5’ TGGCCACTACTCCATCCTGA 3’
Vamp2 - Forward	5’ GCTGGATGACCGTGCAGAT 3’
Vamp2 - Reverse	5’ GATGGCGCAGATCACTCCC 3’
Human Rab27a - Forward	5' GGCACGTTGGGAATCTAGC 3'
Human Rab27a - Reverse	5' AAGCTACGAAACCTCTCCTGC 3'

ARRIVE GUIDELINES

The ARRIVE guidelines (https://arriveguidelines.org/) are a checklist of recommendations to improve the reporting of research involving animals. Key elements of the study design should be included below to better enable readers to scrutinize the research adequately, evaluate its methodological rigor, and reproduce the methods or findings.

Study Design

Groups	Sex	Age	Number (prior to experiment)	Number (after termination)	Littermates (Yes/No)	Other description
2-4 groups per experiment	F/M as indicated in the methods and figure legends	6-12 weeks	N/A	Indicated in the figure legend	No	N/A

Sample Size:

Sample sizes were estimated through power calculations using variability data from prior or pilot studies, with the aim of detecting a 30% difference at 80% power and a significance threshold of $\alpha = 0.05$.

Inclusion Criteria

All mice were included in the study

Exclusion Criteria

None

Randomization

Mice were randomly assigned to different groups.

Blinding

Data analysis were conducted in a blinded manner.

Table S1. Characteristics of human donors used for PBMC-derived macrophage generation and TRIB3 Q84R genotyping.

Sex	Number of individuals	Age (Mean ± S.D.)	Ethnicity
**Male**	20	38.2 ± 11.2	Caucasian (100%)
**Female**	14	43.4 ± 4.9	Caucasian (100%)
