# Supplementary material for: TRIB3 Links Endoplasmic Reticulum Stress to Impaired Efferocytosis in Atherosclerosis
Source: Circ Res. 2025 Nov 4;137(12):1422–42. doi: 10.1161/CIRCRESAHA.125.326839 (PMC12680282; doi:10.1161/CIRCRESAHA.125.326839)

Figure 1A

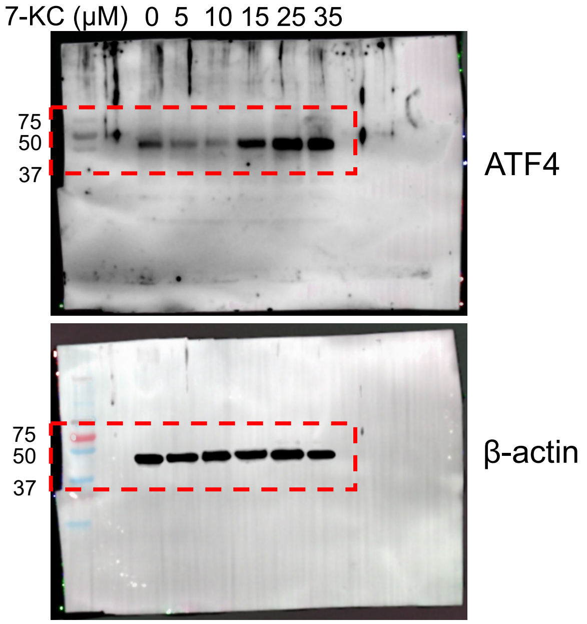

Figure 1B

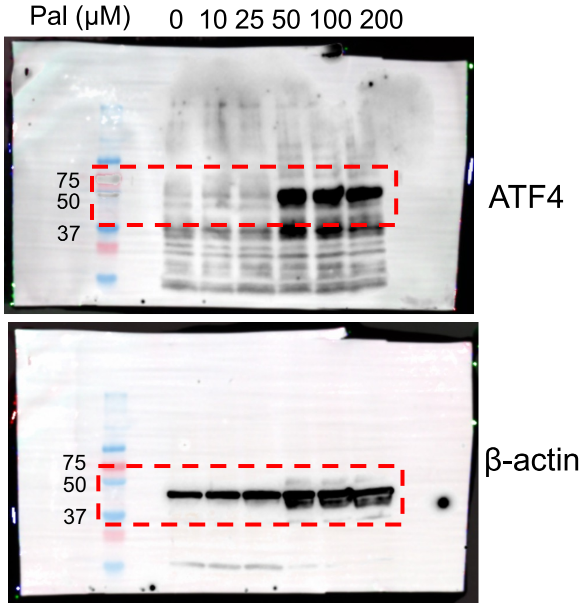

Figure 1F (top panel)

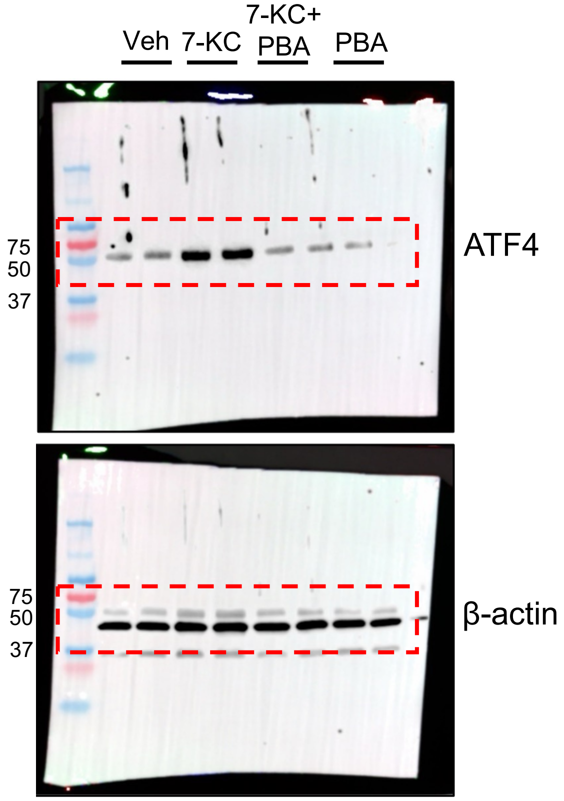

Figure 1F (bottom panel)

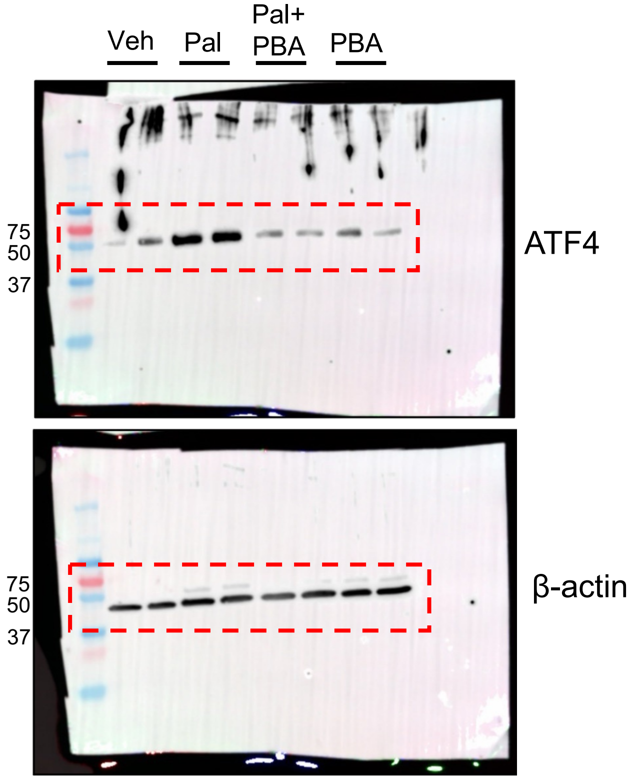

Figure S1C

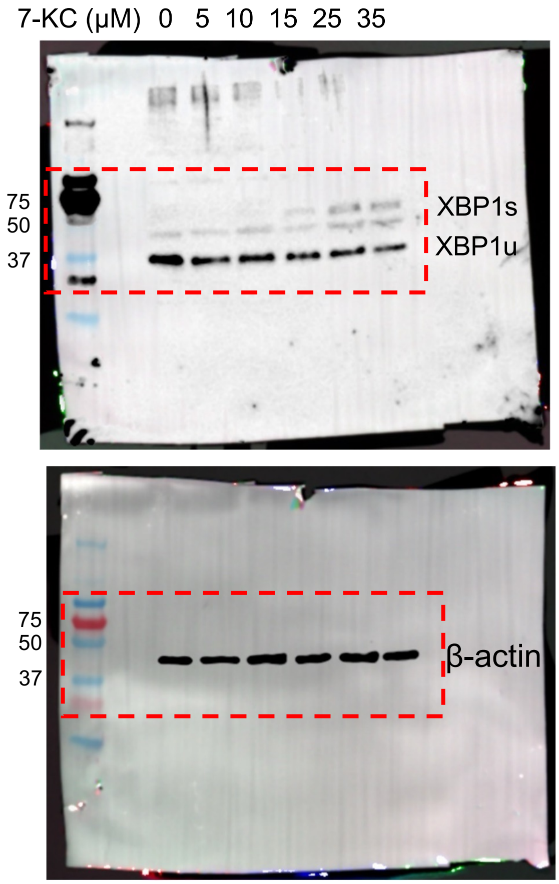

Figure S1D

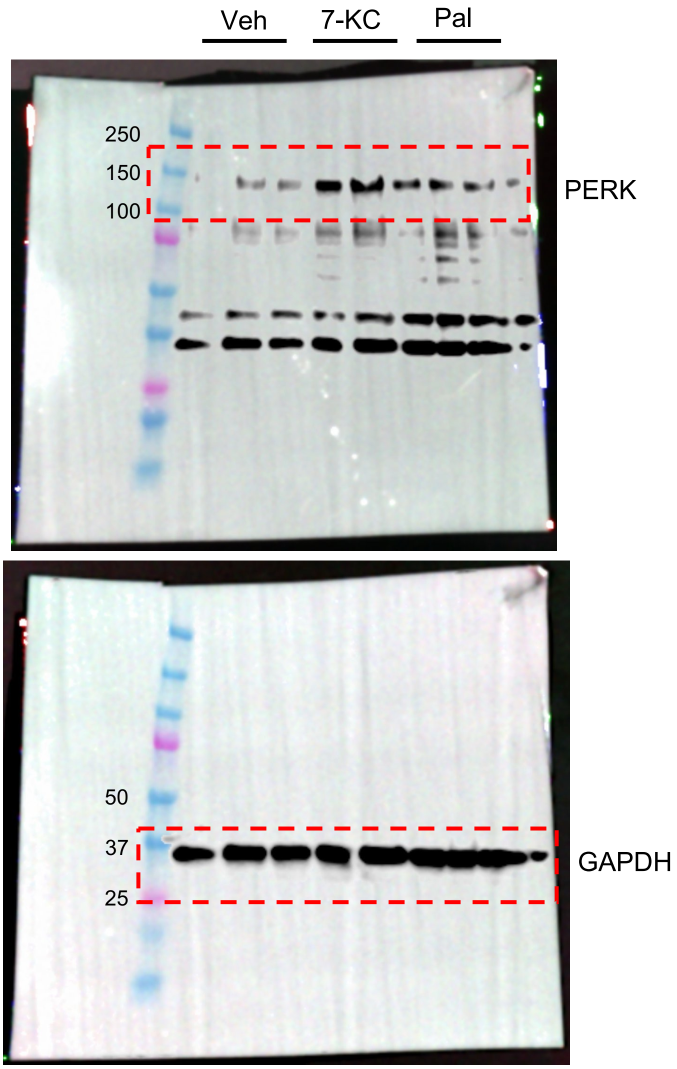

Figure S4E

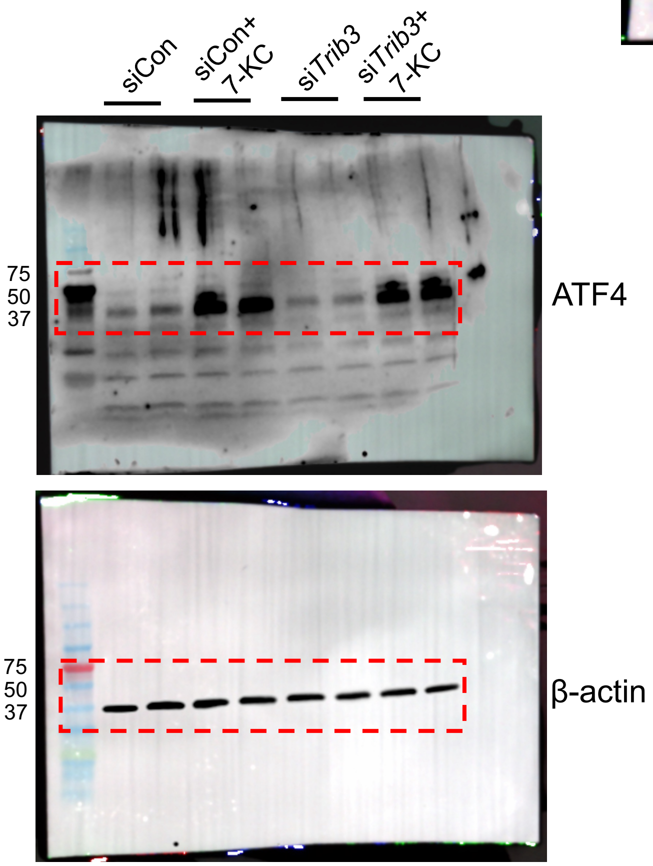

Supplement: Supplementary file 3 [file res-137-1422-s003.pdf]
