# Supplementary material for: TRIB3 Links Endoplasmic Reticulum Stress to Impaired Efferocytosis in Atherosclerosis
Source: Circ Res. 2025 Nov 4;137(12):1422–42. doi: 10.1161/CIRCRESAHA.125.326839 (PMC12680282; doi:10.1161/CIRCRESAHA.125.326839)
Supplement: Supplementary file 2 [file res-137-1422-s002.pdf]

Title: TRIB3 Links Endoplasmic Reticulum Stress to Impaired Efferocytosis in Atherosclerosis

In an effort to promote greater transparency in peer review, the authors and reviewers of this *Circulation Research* article have opted to post the original decision letter with reviewer comments to the authors and the authors' response to reviewers for each significant revision.

June 20, 2025

Dr. Manikandan Subramanian
William Harvey Research Institute
Barts and The London School of Medicine and Dentistry
Queen Mary University of London
Centre for Biochemical Pharmacology
Charterhouse Square
London EC1M6BQ
United Kingdom

RE: CIRCRES/2025/326839: TRIB3 Signaling Impairs Macrophage Efferocytosis in Obesity and Atherosclerosis

Dear Dr. Subramanian:

Your manuscript has been carefully evaluated by three external reviewers and the editors as an Original Research manuscript. We regret to inform you that the paper is not acceptable for publication in its present form.

As you will gather from the reviews, the referees identified a number of conceptual and methodological problems. The editors concur. Major issues include the need for clarification of some findings (Reviewer #1 and Reviewer #3) and further evidence of IRE1 endoribonuclease activity upstream of XBP1 activation and the potential roles of MERTK and LRP1 (Reviewer #2). Among the concerns cited by the reviewers, the editors feel that the request for further in vivo experiments (Reviewer #2, figure 2 comments) is not necessary unless these data are already in hand or can readily be completed in the 90 day timeframe allotted for revision of the manuscript.

Despite these concerns, the editors see this paper as potentially important and wish to encourage revision. If you would like to revise the manuscript in accordance with the suggestions of the reviewers and editors, we would be willing to evaluate a new version. The manuscript would be reviewed again, with no assurance of acceptance.

The Editors strongly encourage you to adhere to the journal's Statistical Reporting Recommendations in your revision, which can be found here: https://www.ahajournals.org/statistical-recommendations.

Upon revision, authors of manuscripts that contain cropped gels/blots will be required to submit a separate PDF file that contains the entire unedited gel for all representative cropped gels in the manuscript. Authors should label each gel as "Full unedited gel for Figure _" and highlight which lanes of the unedited gel correspond to those shown in the cropped images within the manuscript. For more information, please go to https://www.ahajournals.org/res/manuscript-preparation.

All research materials listed in the Methods should be included in the Major Resources Table file, which will be posted online as PDF with the article Supplemental Materials if the manuscript is accepted. A template Major Resources Table file (.docx) is available for download here: AHAJournals_MajorResourcesTable_2019.docx. Authors are required to upload the Table at the revision stage. Authors should reference the PDF in their Methods as follows: "Please see the Major Resources Table in the Supplemental Materials."

To read the comments to authors from the reviewers, please see below.

Please note that revised and resubmitted manuscripts are not assured of publication, and that fewer than 15% of all papers submitted to Circulation Research are eventually published.

Our current guidelines allow authors 90 days to complete the revision. If the manuscript is resubmitted within 90 days, one or more of the original reviewers will be re-consulted; the editors may also choose to obtain additional opinions from new reviewers. If you need more than 90 days to submit a revised paper, please notify the editorial office. In general, extensions over the revision time limit will not be granted except under special circumstances at the editors' discretion.

You are encouraged to log in and start the revision submission process as soon as possible, because all authors must complete disclosure and publication agreement forms BEFORE the revision can be submitted. As corresponding author, you may start the revision process at any time and go to the Copyright & Licensing tab to contact coauthor(s) and send reminders. In addition, please review all revision submission tabs to ensure that you are prepared to provide the appropriate files, checklists, and other items when it is time to submit your revision.

If you choose to revise, please include a detailed response to each of the referees' and editors' comments, providing each comment verbatim in bold followed by your response and giving the exact page number(s), paragraph(s), and line number(s) where each revision was made. If you make substantive changes to the manuscript, please provide a clear description of what you did and where. If you insert important sentences, paragraphs, or sections in response to the comments, please also include them in your response. Please indicate clearly any deletions. Additionally, a marked up version of the revision with the changes highlighted or tracked should be uploaded as a supplemental file. Number each page in the top right corner, using your manuscript number followed by /R1 to denote a first revision.

We are piloting an integration with SciScore (https://www.sciscore.com) to provide authors automatically generated reports during revision submission containing a reproducibility score and tables on rigor adherence and key resources such as antibodies, experimental models, recombinant DNA, and software. You are also welcome to start the revision submission process at any time to receive your report. We strongly encourage you to use the provided report while revising your manuscript to improve the study's reproducibility and reporting quality.

Please ascertain that your revised manuscript adheres to the Instructions to Authors as they appear online at https://www.ahajournals.org/res/author-instructions. Revisions that do not conform to the current limits on numbers of words (8000 total) may be returned to the authors for abbreviation. If you cannot reduce the overall word count, the editors may deem an extended print version appropriate; the authors should provide written assurance that they will cover the costs of the pages that are in excess of these limits. Note that paying for excess display items is not an option. Please refer to the Instructions to Authors for further details regarding our policy on page limits, articles with extended print versions, and related costs. No such limits apply to the online supplementary information, which can include supporting data and/or expanded text to offset the limits on the print version. Such online supplementary information can be cited in the print version as appropriate.

All corresponding authors of articles accepted to AHA Journals are required to link an ORCID iD to their profile in the AHA Journal submission system. To avoid potential processing delays in future, we recommend that you link an ORCID iD to your profile when you submit your revision. To register with ORCID or link your profile, please go to "Modify Profile/Password" on the submission site homepage, and click the link in the "ORCID" section.

We wish to thank you for having submitted this manuscript to Circulation Research.

Check out the latest episode of Discover CircRes podcast for highlights from the latest issue and expert insights into new research.
New episode every month! https://www.ahajournals.org/res/podcast

Sincerely,

Jane E. Freedman, MD
Editor-in-Chief
Circulation Research
An American Heart Association Journal
****
REVIEWER COMMENTS FOR THE AUTHORS:

Reviewer #1:
Singhal et al, demonstrate a that TRIB3 signaling impairs efferocytosis in obesity and atherosclerosis. The demonstrate that activation of the ATF4 branch of the endoplasmic reticulum stress pathway in macrophages lead to the upregulation of Tribbles pseudokinase-3 (TRIB3), which then triggered the downregulation of Rab27a resulting in impaired focal exocytosis of intracellular membrane pools towards the apoptotic cell-containing nascent phagosome. The resultant delay in phagosome closure stalled the efferocytic process and may thus contribute to delayed resolution in obesity and increased necrosis in atherosclerotic plaques. This manuscript is well written, logical and clear and build upon work form this lab in a meaningful and significant manner. The combination of innovative in vitro efferocytosis approaches in murine macrophages, human macrophage and human plaque data as well as in vivo obesity and atherosclerosis studies make this very compelling and convincing. The authors used the correct controls in their studies and interpreted their data in an impactful manner. Overall this way a nice paper and I only have minor comments.

Fig 1. Title says in vivo but it is unclear which panel is in vivo. This figure appears to be in vitro work. Also, there are 4 stars in the figs and the p value should be noted in the fig legend.
Fig 2. results and figure: You stated in the results section for Fig 2, "..we first developed a mouse model of high-fat diet-induced obesity…" What did you develop new here? Or did you apply an established model of high-fat diet indued obesity? I would specify that this is "immunoreactive LXA4" in the text since this was detected by ELISA and not LC-MS-MS. ELISA will pick up LXA4 and associated isomers so it's not as specific as LC-MS-MS. Fig 2. legend should specify p value for 4 stars.
The design in Fig 3e is clever. Also, Fig 3. legend should specify p value for 4 stars.
Fig 6 is very compelling and supports the proposed mechanism
Is it known that impaired phagosome closure leads to increased inflammation? If so, that may also be a discussion point.

Reviewer #2:
The manuscript by Singhal et al. investigates the role of the ATF4 branch of the unfolded protein response (UPR) in promoting TRIB3-dependent efferocytosis. The study is clear, well-executed, and addresses a timely question of significant relevance to the field. The authors are to be commended for their rigorous and insightful work, incorporating in vitro, in vivo, and human validation. Nevertheless, this reviewer offers a few suggestions and comments that, if addressed, could further enhance the rigor and depth of the study

Figure 1

IRE1 is a direct activator of XBP1, mediating its splicing via its endoribonuclease activity. To strengthen the mechanistic link, the authors could provide evidence of IRE1 endoribonuclease activity upstream of XBP1 activation. Inclusion of PERK expression levels as an additional control would also be helpful to contextualize the specificity of the UPR branch involved.

The efferocytosis data following treatment with 4-PBA are intriguing; however, given the broad activity of chemical chaperones, the use of more specific inhibitors of IRE1's endoribonuclease activity—such as STF-083010, MKC8866, or 4μ8C—would offer more direct insight into the role of IRE1-XBP1 signaling in this process.

Figure 2

Depending on the outcomes of the experiments using specific inhibitors of IRE1 endoribonuclease activity in Figure 1, it would be valuable to extend these findings to the in vivo context presented in Figure 2.

In Figure 2e, the authors interpret the observed phenomenon as efferocytosis. However, it remains unclear how they distinguish true efferocytosis from other forms of cell-cell interactions or microvesicle-mediated communication. Additional validation or clarification is warranted to ensure accurate interpretation of these findings.

The use of palmitate to induce foamy macrophages is relevant, as it models the phenotype commonly observed in obese adipose tissue. However, the authors primarily use peritoneal macrophages throughout the study. To strengthen the physiological relevance of their findings—particularly with regard to efferocytosis—key observations should be corroborated in adipose tissue macrophages, either through ex vivo analysis or in vivo models. This could be especially informative if the authors can reliably identify macrophages that have internalized Ly6G+ neutrophils.

Finally, could the authors assess levels of 7-ketocholesterol (7-KC) in peritoneal macrophages isolated from obese mice? This would provide additional mechanistic insight and reinforce the metabolic relevance of their model.

Figure 3

It is unclear why palmitate loading is no longer included in this figure. While both palmitate and 7-ketocholesterol (7-KC) are used to model lipid-induced stress, they likely activate distinct cellular pathways. Notably, 7-KC is known to disrupt membrane order, which may serve as a more direct trigger of the ER stress response—unlike palmitate. A brief rationale for this shift in experimental design would be helpful.

To further explore the mechanistic role of 7-KC, the authors could assess whether removal of excess membrane cholesterol using methyl-β-cyclodextrin (MβCD) following 7-KC loading alters the observed phenotype. This would help determine whether membrane cholesterol content is a key mediator of the stress response and downstream efferocytic activity

Additionally, if the authors observe an increase in IRE1 endoribonuclease activity upon 7-KC treatment, it would be important to examine the expression of MERTK and LRP1. Quantifying these receptors would help establish a mechanistic link to reference (18) and provide a more complete picture of how ER stress might regulate efferocytosis through specific phagocytic receptors

Figure 4

Have the authors investigated whether palmitate also regulates the expression of Trib3 and Rab27a? This comparison would help clarify whether the observed effects are specific to 7-KC–induced ER stress or are part of a broader lipid stress response

In addition, since ATF4 has been reported to induce IRE1, it is important to determine whether the expression of Trib3 and Rab27a is independent of IRE1 signaling. The authors should assess whether these genes are still upregulated in the absence or inhibition of IRE1 activity to confirm that their expression is specifically mediated by the ATF4 branch and not downstream of IRE1-XBP1 signaling

Figure 6 ans S6

The authors are encouraged to leverage recently available murine and human scRNAseq data to explore and confirm the regulation of Trib3 a d Rab27a expression in plaque-associated macrophages

Reviewer #3:
Reported herein is a new mechanism by which macrophage clearance of dying cells is altered in atherosclerosis and obesity. The novel findings are interesting and integrated with human relevance and a new molecular mechanisms. The following comments may be considered to increase experimental rigor.

Elevated fat is utilized to model obesity and ER-stress, in macrophages and this is found to impair a signature of apoptotic cell feeding. The data are convincing. Are there data to indicate if this phenotype is at the stage of recognition (binding) versus engulfment?

In an interesting peritoneal model of how obesity affects inflammation resolution, it is found that the decrease in neutrophil numbers over time was significantly slower in the obese mice as compared with the lean mice (which was reversed upon administration of 4-PBA). This is correlated with efferocytosis efficiency in the peritoneum in Figure2. In vivo phagocytosis assays can be complicated to ascertain engulfment. Could more representative images be shown to ascertain experimental rigor?

Interestingly the engulfment effects of obesity were selective (bacterial uptake not effected). The data are convincing in Figure 3. Utilizing an innovative method, the findings also suggest that apoptotic cells were contained within partially sealed phagosomes.
Impairments in efferocytosis are uniquely ascribed to activation of an ATF4-TRIB3-Rab27a pathway in Figure 4. Panel 4m and 4n appear to be missing siTrib3 controls.

Importantly the phenotype is reproduced in human macrophages in Figure 5 and the link to the TRIB3 SNP is relevant and important in Figure 6.

As predicted from the aforementioned data, Trib3 deficiency in bone marrow cells led to reduced plaque necrosis. Is this a function of changes in macrophage number?

Limitations of the study may be expanded in the discussion.

Response to Editors and Reviewers

Editor comments:
As you will gather from the reviews, the referees identified a number of conceptual and methodological problems. The editors concur. Major issues include the need for clarification of some findings (Rev 1 and Rev 3) and further evidence of IRE1 endoribonuclease activity upstream of XBP1 activation and the potential roles of MERTK and LRP1 (Rev 2). Among the concerns cited by the reviewers, the editors feel that the request for further in vivo experiments (Rev 2, figure 2 comments) is not necessary unless these data are already in hand or can readily be completed in the 90 day timeframe allotted for revision of the manuscript.

Thank you for the opportunity to revise our manuscript. As outlined below, we have experimentally addressed the major concerns raised by the reviewers. Specifically, we provide: (i) evidence for IRE1-mediated XBP1 activation, (ii) clarification of the specificity of the PERK–ATF4 pathway in regulating TRIB3, (iii) data demonstrating that MERTK and LRP1 are unaltered in our experimental setting, and (iv) validation of the *in vivo* peritoneal cavity efferocytosis assay by showing Ly6G$^+$ neutrophils within F4/80$^+$ macrophages using fluorescence microscopy of FACS-sorted cells. Importantly, our new data indicate that the IRE1 pathway does not regulate TRIB3 or efferocytosis in our model. Therefore, the additional *in vivo* experiment requested by Reviewer 2 to test the role of IRE1 was not pursued.

Reviewer comments:

Reviewer #1:
Singhal et al, demonstrate a that TRIB3 signaling impairs efferocytosis in obesity and atherosclerosis. The demonstrate that activation of the ATF4 branch of the endoplasmic reticulum stress pathway in macrophages lead to the upregulation of Tribbles pseudokinase-3 (TRIB3), which then triggered the downregulation of Rab27a resulting in impaired focal exocytosis of intracellular membrane pools towards the apoptotic cell-containing nascent phagosome. The resultant delay in phagosome closure stalled the efferocytic process and may thus contribute to delayed resolution in obesity and increased necrosis in atherosclerotic plaques. This manuscript is well written, logical and clear and build upon work form this lab in a meaningful and significant manner. The combination of innovative in vitro efferocytosis approaches in murine macrophages, human macrophage and human plaque data as well as in vivo obesity and atherosclerosis studies make this very compelling and convincing. The authors used the correct controls in their studies and interpreted their data in an impactful manner. Overall this way a nice paper and I only have minor comments.

Thanks for the appreciation of our study.

Fig 1. Title says in vivo but it is unclear which panel is in vivo. This figure appears to be in vitro work. Also, there are 4 stars in the figs and the p value should be noted in the fig legend.

Thanks for the comment. The figure legend title has been corrected. Absolute p-values are now indicated in each figure including the relevant comparisons and the statistical test used.

Fig 2. results and figure: You stated in the results section for Fig 2, "..we first developed a mouse model of high-fat diet-induced obesity…" What did you develop new here? Or did you apply an established model of high-fat diet indued obesity?

Thanks for the comment. The sentence has now been corrected and reads "*To examine whether lipid accumulation-induced ER stress impairs Mϕ efferocytosis in vivo, we used a mouse model of high-fat diet-induced obesity (**Figure 2A, schematic**) characterized by accumulation of neutral lipids in peritoneal cavity Mϕs (**Figure S2A**)*.".

I would specify that this is "immunoreactive LXA4" in the text since this was detected by ELISA and not LC-MS-MS. ELISA will pick up LXA4 and associated isomers so it's not as specific as LC-MS-MS.

Thanks for raising this important point. The figure axis label and legend have been corrected.

Fig 2. legend should specify p value for 4 stars.
The design in Fig 3e is clever. Also, Fig 3. legend should specify p value for 4 stars.

P values are now indicated for each comparison in the figure including details of the relevant statistical test they were derived from.

Fig 6 is very compelling and supports the proposed mechanism
Is it known that impaired phagosome closure leads to increased inflammation? If so, that may also be a discussion point.

Thanks for the question. Impaired closure of phagosomes as well as delayed lysosomal fusion during efferocytosis results in secondary necrosis of apoptotic cells and the release of pro-inflammatory mediators, thereby promoting chronic inflammation (PMIDs: 28847720, 28942921). Moreover, impaired phagosome closure could result in delayed phagosome resolution (PMID: 34180943) with consequent impact on continuous efferocytosis and resolution of inflammation (PMID: 28942921).

Reviewer #2:

The manuscript by Singhal et al. investigates the role of the ATF4 branch of the unfolded protein response (UPR) in promoting TRIB3-dependent efferocytosis. The study is clear, well-executed, and addresses a timely question of significant relevance to the field. The authors are to be commended for their rigorous and insightful work, incorporating in vitro, in vivo, and human validation. Nevertheless, this reviewer offers a few suggestions and comments that, if addressed, could further enhance the rigor and depth of the study

We thank the Reviewer for their appreciation of our work and for providing constructive suggestions.

Figure 1

IRE1 is a direct activator of XBP1, mediating its splicing via its endoribonuclease activity. To strengthen the mechanistic link, the authors could provide evidence of IRE1 endoribonuclease activity upstream of XBP1 activation. Inclusion of PERK expression levels as an additional control would also be helpful to contextualize the specificity of the UPR branch involved.

We thank the Reviewer for their comment. Our new data show that treating macrophages with 4μ8C, an inhibitor of IRE1 endoribonuclease activity, prevents the 7-KC- and palmitate-induced generation of spliced XBP1 (**Reviewer Figure 1A**). This demonstrates that spliced XBP1 is generated specifically via IRE1 RNase activity.

We also present new data demonstrating that both 7-KC and palmitate increase PERK levels, which lie upstream of ATF4 (**Reviewer Figure 1B**). These new data are presented as supplementary figures, **Figure S1D and S1E**, in the revised manuscript.

Reviewer Figure 1

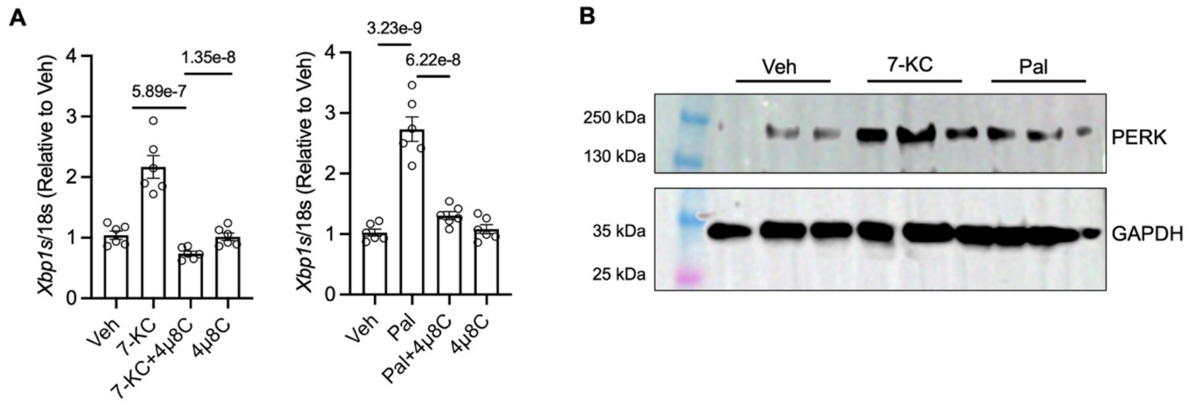

Taken together, these findings indicate that 7-KC and palmitate activate multiple UPR pathways. However, as highlighted in response to the next question, it is important to note that although 7-KC and palmitate activate all branches of the UPR, the impairment in efferocytosis can be reversed only via inhibition of the PERK/ATF4 pathway (**Reviewer Figure 2A**), but not the IRE1-XBP1 pathway or the ATF6 pathway (**Reviewer Figure 2A**).

The efferocytosis data following treatment with 4-PBA are intriguing; however, given the broad activity of chemical chaperones, the use of more specific inhibitors of IRE1's endoribonuclease activity—such as STF-083010, MKC8866, or 4μ8C—would offer more direct insight into the role of IRE1-XBP1 signaling in this process.

Thank you for the suggestion. To address this point more broadly, we incubated 7-KC–treated macrophages with specific inhibitors of IRE1 endoribonuclease (4μ8C), PERK (ISRIB), and ATF6 (CeapinA7) to test whether the efferocytosis defect could be rescued. Strikingly, only ISRIB reversed the 7-KC-induced impairment in efferocytosis, while the other inhibitors had no effect (**Reviewer Figure 2A**). These results indicate that the PERK-ATF4 pathway is the critical regulator of TRIB3-mediated impairment in macrophage efferocytosis, consistent with our complementary *in vitro* and *in vivo* findings (**Figure 3H and 3I**). These new data are presented as **Figure 3G** in the revised manuscript.

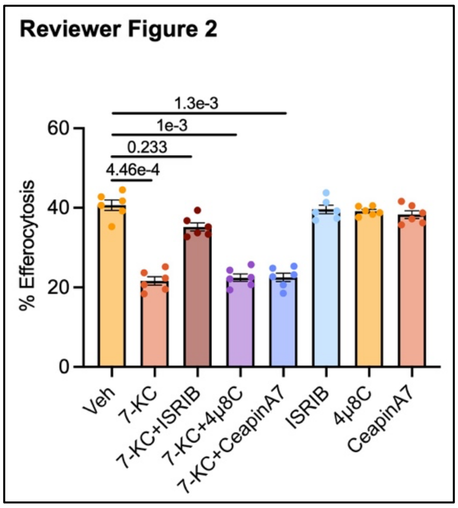

Figure 2

Depending on the outcomes of the experiments using specific inhibitors of IRE1 endoribonuclease activity in Figure 1, it would be valuable to extend these findings to the in vivo context presented in Figure 2.

Thank you for the suggestion. As shown above, inhibition of IRE1 endoribonuclease activity with 4μ8C (**Supplementary Figure S1E**) did not reverse the 7-KC-induced impairment in efferocytosis *in vitro* (**Reviewer Figure 2A)**. Therefore, we did not extend the analysis of the IRE1 pathway to the *in vivo* setting.

Instead, we draw your attention to **Figure 3I** and **Figure 4N**, where we show that knockdown of ATF4 or TRIB3 reverses the obesity-induced impairment of efferocytosis *in vivo*. These results provide strong evidence that the PERK–ATF4–TRIB3 pathway, plays a central role in driving ER stress-mediated impairment of efferocytosis in lipid-loaded macrophages.

In Figure 2e, the authors interpret the observed phenomenon as efferocytosis. However, it remains unclear how they distinguish true efferocytosis from other forms of cell-cell interactions or microvesicle-mediated communication. Additional validation or clarification is warranted to ensure accurate interpretation of these findings.

Thank you for this insightful comment. To validate that the F4/80^{+}Ly6G^{+} cells we detected indeed represent efferocytosis events, we FACS-sorted these cells from the peritoneal lavage, plated them on coverslips, and examined them by fluorescence microscopy. As shown in **Reviewer Figure 3**, F4/80^{+} macrophages (green) contained Ly6G^{+} cells, both intact and partially degraded, consistent with ongoing efferocytosis. The size and intensity of the Ly6G^{+} signal argue against the possibility that these events reflect transfer of neutrophil-derived microvesicles. Taken together, these observations provide strong evidence that the F4/80^{+}Ly6G^{+} events detected by flow cytometry represent bona fide efferocytic events. These data are now included as **Supplementary Figure S2D** in the revised manuscript.

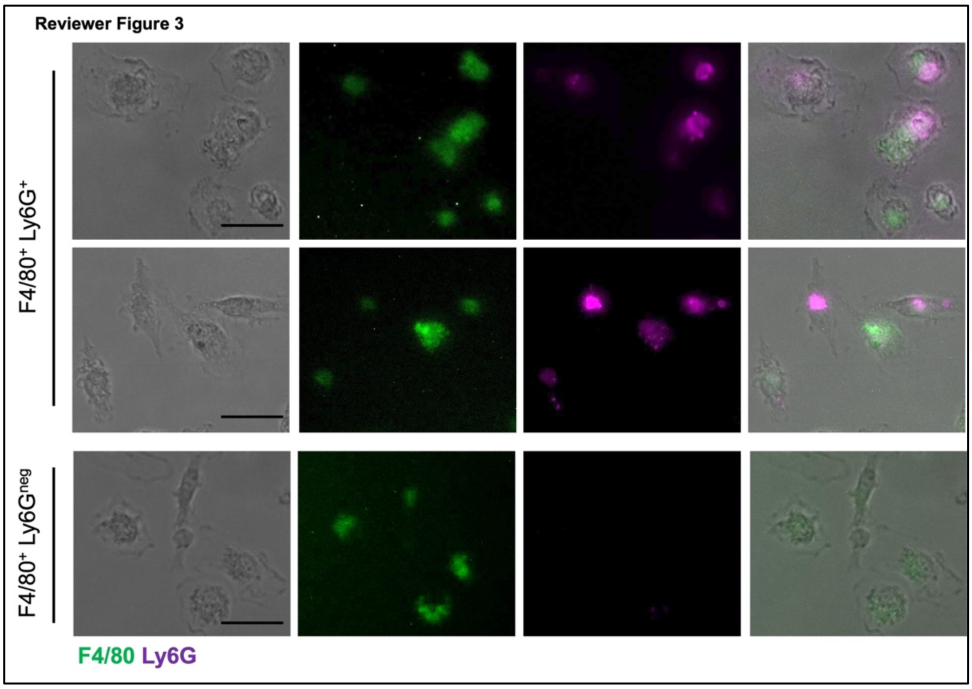

The use of palmitate to induce foamy macrophages is relevant, as it models the phenotype commonly observed in obese adipose tissue. However, the authors primarily use peritoneal macrophages throughout the study. To strengthen the physiological relevance of their findings—particularly with regard to efferocytosis—key observations should be corroborated in adipose tissue macrophages, either through ex vivo analysis or in vivo models. This could be especially informative if the authors can reliably identify macrophages that have internalized Ly6G+ neutrophils.

We thank the reviewer for this insightful comment. In our study, we employed multiple lipid stimuli including 7KC, palmitate, and oxidized LDL (oxLDL), to generate lipid-loaded macrophages (**Supplementary Figure S1A and S1B**) and mimic different aspects of the

lipid-rich microenvironment encountered in obesity and atherosclerosis. Importantly, we also demonstrate that macrophages in the peritoneal cavity of obese mice are themselves lipid-laden (**Supplementary Figure S2A**) and exhibit ER stress (**Figure 2B**), making them a physiologically relevant model to investigate how lipid-induced ER stress impacts efferocytosis.

We selected the peritoneal cavity as our primary *in vivo* system because it is a tractable model in which apoptotic cells, whether endogenous or exogenously introduced, can be readily tracked. This enables robust and quantitative analysis of efferocytosis, as demonstrated in this manuscript. In contrast, studying efferocytosis within adipose tissue presents significant technical challenges. First, there are currently no experimental models that allow apoptosis to be selectively induced in specific adipose depots in a controlled manner, apart from genetic approaches such as adipocyte-targeted caspase-8 activation, which causes widespread systemic adipocyte apoptosis. Second, adipocytes are exceptionally large cells, and their clearance differs mechanistically from canonical efferocytosis. Dead adipocytes are typically surrounded by multiple macrophages that form "crown-like" structures and are cleared piecemeal in part through exophagy (PMIDs: 18390487, 27044658, 16150820), rather than by a single macrophage engulfing a single apoptotic cell. Thus, while adipose tissue macrophages play key roles in obesity biology, we hope the Reviewer will agree with us that they are not optimal for mechanistic *in vivo* studies of efferocytosis.

Moreover, previous studies have shown that obesity impairs efferocytosis not only in adipose tissue but also in other tissues, such as the lung and liver (PMIDs: 23154082, 36521495), underscoring the broader systemic impact of obesity on this process. Taken together, our peritoneal cavity model, which recapitulates the lipid-loaded and ER-stressed state of macrophages in obesity, provides a physiologically relevant and experimentally tractable system to delineate how lipid-induced stress alters efferocytosis *in vivo*.

Finally, could the authors assess levels of 7-ketocholesterol (7-KC) in peritoneal macrophages isolated from obese mice? This would provide additional mechanistic insight and reinforce the metabolic relevance of their model.

Thank you for this suggestion. We previously showed that peritoneal macrophages from obese mice display increased LipidTOX staining, consistent with intracellular lipid accumulation under obese conditions (**Supplementary Figure S2A**). Our new data further demonstrate that peritoneal macrophages from obese mice contain ~1.5-fold higher levels of 7-KC compared with lean controls (**Reviewer Figure 4**).

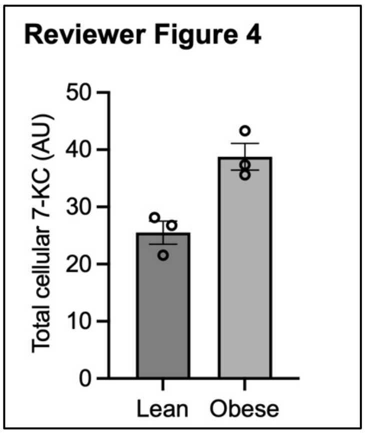

Although these data provide additional rationale for its use as a relevant *in vitro* model, we would like to emphasize that impaired efferocytosis is a general consequence of ER stress and ATF4 pathway activation, rather than being dependent on a single lipid mediator. We observed a consistent phenotype using diverse ER stress inducers, including 7-KC, palmitate, oxLDL, and chemical stressors such as tunicamycin, thapsigargin, and DTT (**Figure 1E, and Supplementary Figures S1H and S4H**). In all cases, the efferocytosis defect was rescued by inhibition of the ATF4-TRIB3 signaling cascade (**Figures 3H, and 4F, and Supplementary Figure S4H**). These findings highlight that the metabolic relevance of our model is not restricted to 7-KC but instead reflects a broader pathogenic mechanism by which lipid-induced ER stress impairs macrophage efferocytosis in obesity and atherosclerosis.

Figure 3

It is unclear why palmitate loading is no longer included in this figure. While both palmitate and 7-ketocholesterol (7-KC) are used to model lipid-induced stress, they likely activate distinct cellular pathways. Notably, 7-KC is known to disrupt membrane order, which may serve as a more direct trigger of the ER stress response—unlike palmitate. A brief rationale for this shift in experimental design would be helpful.

Thank you for the comment. As detailed above, our data show that distinct lipid species including 7-KC, palmitate, and oxLDL, serve as inducers of ER stress and converge on activation of the ATF4-TRIB3 pathway, which underlies impaired efferocytosis. In this figure, we chose to highlight 7-KC to maintain clarity and focus, but all key findings have been validated across lipid stressors. Specifically, siRNA-mediated knockdown of TRIB3 in macrophages exposed to 7-KC, palmitate, or oxLDL consistently restored efferocytosis (**Figure 4F, and Supplementary Figure S4H**), underscoring the central and shared role of TRIB3 in this process.

Furthermore, we now present new data showing that both phagosome sealing (**Reviewer Figure 5A**) and focal exocytosis (**Reviewer Figure 5B**) are similarly impaired in macrophages exposed to palmitate and oxLDL, demonstrating that not only the signaling pathway but also the cell biological mechanisms disrupting efferocytosis are conserved

across distinct lipid-induced ER stressors. These results are now included as **Supplementary Figure S3C and S3D**.

Reviewer Figure 5

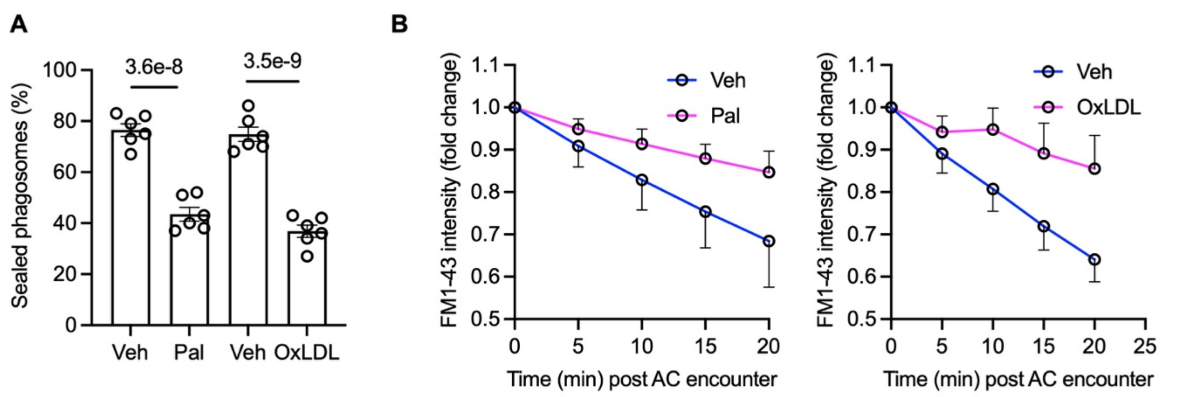

To further explore the mechanistic role of 7-KC, the authors could assess whether removal of excess membrane cholesterol using methyl-β-cyclodextrin (MβCD) following 7-KC loading alters the observed phenotype. This would help determine whether membrane cholesterol content is a key mediator of the stress response and downstream efferocytic activity.

Thank you for this suggestion. Membrane cholesterol is a critical component of lipid rafts, which regulate receptor organization and signaling and are therefore essential for efferocytosis (PMID: 36130624). In line with this, we found that depletion of membrane cholesterol (**Reviewer Figure 6A**) using MβCD reduced efferocytosis (**Reviewer Figure 6B**) in both control and 7-KC–treated macrophages, making it challenging to attribute the observed effects specifically to 7-KC–induced changes. We acknowledge, however, that changes to plasma membrane fluidity and lipid raft organization my additionally contribute to efferocytosis impairment in lipid-loaded ER stressed macrophages.

Reviewer Figure 6

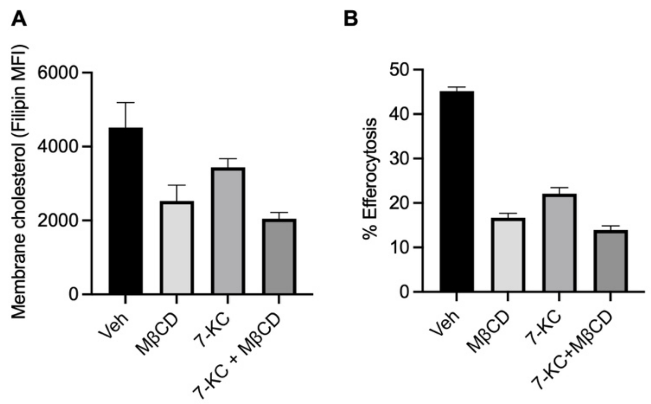

Additionally, if the authors observe an increase in IRE1 endoribonuclease activity upon 7-KC treatment, it would be important to examine the expression of MERTK and LRP1. Quantifying

these receptors would help establish a mechanistic link to reference (18) and provide a more complete picture of how ER stress might regulate efferocytosis through specific phagocytic receptors.

Thank you for the comment. We present new data demonstrating that macrophages exposed to either 7-KC or palmitate do not display altered cell surface levels of key efferocytosis receptors such as Mertk and LRP-1 as determined by flow cytometry (**Reviewer Figure 7A**). Consistent with these data, we also report that apoptotic cell recognition and binding are unaffected under these conditions (**Reviewer Figure 7B**). These data are presented as **Supplementary Figures S3A and S3B** in the revised manuscript.

Reviewer Figure 7

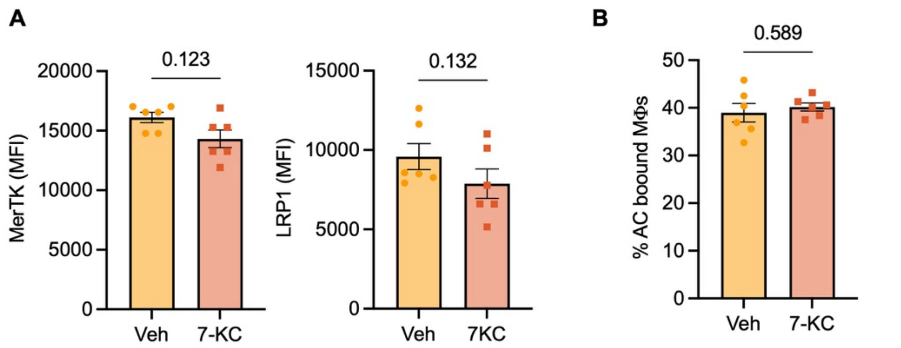

Together, these findings indicate that the impairment in efferocytosis in ER-stressed macrophages is primarily due to defective apoptotic cell engulfment, arising from impaired focal exocytosis and defective closure of apoptotic cell–containing phagosomes.

This outcome differs from the report by Yildrim et al., who observed reduced Mertk and LRP-1 levels following palmitate exposure. We believe this discrepancy may arise from differences in experimental design and in the measurement of total cellular protein versus cell surface expression as explained below. Specifically, Yildrim et al. employed acute stress conditions (500 µM palmitate for 6 h), whereas we used optimized conditions that induce chronic, compensated ER stress (50 µM palmitate for 18 h). Furthermore, Yildirim et al. assessed Mertk and LRP-1 levels by western blotting in whole-cell lysates, reflecting total cellular protein content. In contrast, we specifically measured cell surface levels of Mertk and LRP-1, which is most relevant for their function as efferocytic receptors.

Figure 4

Have the authors investigated whether palmitate also regulates the expression of Trib3 and Rab27a? This comparison would help clarify whether the observed effects are specific to 7-KC–induced ER stress or are part of a broader lipid stress response

Thank you for the question. We observed that macrophages exposed to palmitate or oxLDL, similar to those treated with 7-KC, show increased expression of TRIB3 and reduced levels

of *Rab27a* (**Supplementary Figure S4G**). Moreover, siRNA-mediated knockdown of Trib3 in palmitate- or oxLDL-treated macrophages restored efferocytosis (**Supplementary Figure S4H**). Together, these findings demonstrate that activation of the ATF4-TRIB3-Rab27a axis is a common mechanism underlying efferocytosis impairment across distinct lipid-induced stressors.

In addition, since ATF4 has been reported to induce IRE1, it is important to determine whether the expression of Trib3 and Rab27a is independent of IRE1 signaling. The authors should assess whether these genes are still upregulated in the absence or inhibition of IRE1 activity *to confirm that their expression is specifically mediated by the ATF4 branch and not downstream of IRE1-XBP1 signaling*.

Thank you for raising this important point. To directly test whether *Trib3* and *Rab27a* expression is regulated via the IRE1-XBP1 pathway, we incubated 7-KC-exposed macrophages with 4μ8C, a selective inhibitor of IRE1 endoribonuclease activity. As shown in **Reviewer Figure 8**, inhibition of IRE1 had no effect on the 7-KC-induced upregulation of Trib3 or the downregulation of Rab27a. These results (**Supplementary Figure S5I**) indicate that the regulation of the TRIB3-Rab27a axis occurs independently of IRE1–XBP1 signaling.

Reviewer Figure 8

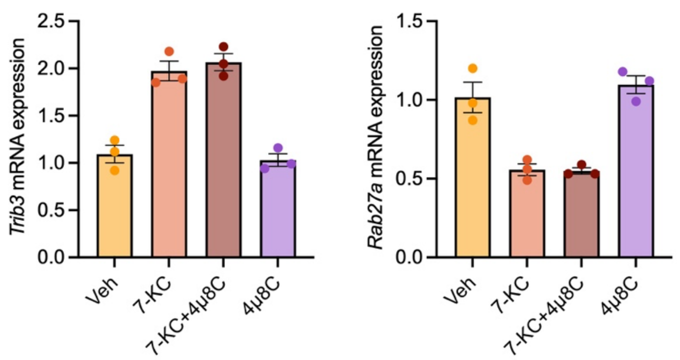

Together with our ATF4 siRNA experiments (**Figure 4B and 4C**), these findings reinforce that ATF4 is the critical upstream mediator linking lipid-induced ER stress to efferocytosis defects, via activation of the TRIB3-Rab27a pathway.

Figure 6 ans S6

The authors are encouraged to leverage recently available murine and human scRNAseq data to explore and confirm the regulation of Trib3 a d Rab27a expression in plaque-associated macrophages

We thank the Reviewer for this helpful suggestion. To address this question and evaluate the translational relevance of our key mechanistic findings in the context of human atherosclerosis, we analyzed publicly available scRNA-seq dataset from 15 human atherosclerotic plaques (GSE260657). Our analysis revealed that, consistent with our *in vitro*,

ex vivo, and *in vivo* findings, elevated TRIB3 expression in human plaque macrophages was associated with reduced RAB27A expression (**Reviewer Figure 9**). This provides independent validation of our results using an external dataset. These data are now included as **Figure 6C** in the revised manuscript.

Reviewer Figure 9

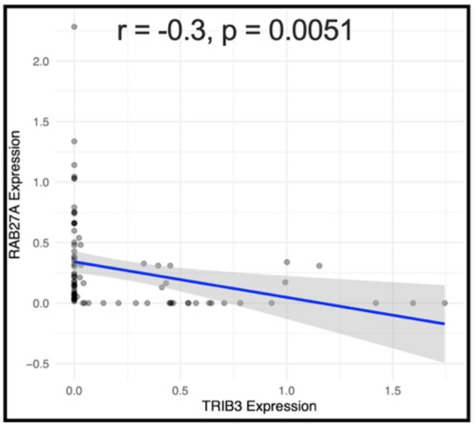

Reviewer #3:

Reported herein is a new mechanism by which macrophage clearance of dying cells is altered in atherosclerosis and obesity. The novel findings are interesting and integrated with human relevance and a new molecular mechanisms. The following comments may be considered to increase experimental rigor.

Elevated fat is utilized to model obesity and ER-stress, in macrophages and this is found to impair a signature of apoptotic cell feeding. The data are convincing. Are there data to indicate if this phenotype is at the stage of recognition (binding) versus engulfment?

Thank you for the question. To address this, we specifically assessed the ability of macrophages to recognize and bind apoptotic cells by treating them with cytochalasin D, an actin polymerization inhibitor that allows apoptotic cell binding but blocks subsequent engulfment. As shown in **Reviewer Figure 7B** (response to Reviewer #2), we found no significant difference in apoptotic cell binding between groups. This is further supported by our new data (**Reviewer Figure 7A**), demonstrating that macrophages exposed to 7KC or palmitate do not exhibit altered expression of key efferocytosis receptors, including Mertk and LRP1. Together, these results indicate that lipid-induced chronic compensated ER stress impairs efferocytosis primarily at the engulfment stage, by limiting focal exocytosis of endomembranes toward the apoptotic cell-containing phagosome and thereby preventing efficient phagosomal closure. These new data are presented in **Supplementary Figures S3A and S3B** in the revised manuscript

In an interesting peritoneal model of how obesity affects inflammation resolution, it is found that the decrease in neutrophil numbers over time was significantly slower in the obese mice as compared with the lean mice (which was reversed upon administration of 4-PBA). This is correlated with efferocytosis efficiency in the peritoneum in Figure2. In vivo phagocytosis assays can be complicated to ascertain engulfment. Could more representative images be shown to ascertain experimental rigor?

Thank you for this insightful comment. To validate that the F4/80$^+$Ly6G$^+$ cells we detected indeed represent efferocytosis events, we FACS-sorted these cells from the peritoneal lavage, plated them on coverslips, and examined them by fluorescence microscopy. As shown in **Reviewer Figure 3** (Response to Reviewer #2), F4/80$^+$ macrophages (green) contained Ly6G$^+$ cells, both intact and partially degraded, consistent with ongoing efferocytosis. These data provide strong evidence that the F4/80$^+$Ly6G$^+$ events detected by flow cytometry represent bona fide efferocytic events. These data are now included as **Supplementary Figure S2D** in the revised manuscript.

Interestingly the engulfment effects of obesity were selective (bacterial uptake not effected). The data are convincing in Figure 3. Utilizing an innovative method, the findings also suggest that apoptotic cells were contained within partially sealed phagosomes.

Thank you.

Impairments in efferocytosis are uniquely ascribed to activation of an ATF4-TRIB3-Rab27a pathway in Figure 4. Panel 4m and 4n appear to be missing siTrib3 controls.

In Figure 4M and 4N, we specifically addressed whether the obesity-induced increase in TRIB3 is causative in driving efferocytosis impairment. To test this, we used an siRNA-based approach to selectively knock down TRIB3 in peritoneal macrophages from obese mice, thereby reducing TRIB3 expression to levels comparable to those observed in lean mice (**Figure 4M**).

Including an siTRIB3 control in lean mice would, by design, lower TRIB3 expression below its physiological baseline. This would instead test whether *endogenous* TRIB3 levels are required for efferocytosis, which is a different biological question. Based on our *in vitro* experiments, reducing TRIB3 below baseline does not impair efferocytosis (**Figure 4F**). For this reason, we focused on the obesity setting, where elevated TRIB3 levels represent the relevant pathogenic condition.

Importantly the phenotype is reproduced in human macrophages in Figure 5 and the link to the TRIB3 SNP is relevant and important in Figure 6.

Thanks.

As predicted from the aforementioned data, Trib3 deficiency in bone marrow cells led to reduced plaque necrosis. Is this a function of changes in macrophage number?

Thanks for the question. There was no change in the number of lesional macrophages (Mac2+ cells) between WT and TRIB3 KO bone marrow chimeric mice (**Reviewer Figure 10**). These data are now presented as **Supplementary Figure S7H**.

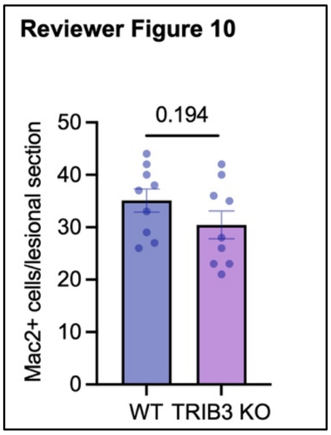

Limitations of the study may be expanded in the discussion.

Thanks for the suggestion. Limitations of the study has now been included in the Discussion. "*Our study has certain limitations. First, while we modeled adaptive ER stress to better mimic the state of lesional Mϕs, ER stress is heterogeneous, and other pathways (e.g., IRE1-FMRP signaling (18)) may also contribute to impaired efferocytosis depending on the severity of stress experienced by Mϕs. Second, our murine atherosclerosis data suggest a key role for the suppression of Mϕ-TRIB3 in enhancing efferocytosis and promoting plaque stability; however, it is possible that some of the observed effects may also arise from loss of TRIB3 signaling in non-Mϕ leukocytes such as T- and B- cells, which, although expressing only low levels of TRIB3, could still contribute. Finally, the TRIB3 Q84R variant is associated with enhanced insulin resistance (36), and the relative contributions of altered glucose homeostasis versus impaired efferocytosis to the accelerated atherosclerosis phenotype in patients remain to be tested.*"

October 3, 2025

Dr. Manikandan Subramanian
William Harvey Research Institute
Barts and The London School of Medicine and Dentistry
Queen Mary University of London
Centre for Biochemical Pharmacology
Charterhouse Square
London EC1M6BQ
United Kingdom

RE: CIRCRES/2025/326839R1: Endoplasmic Reticulum Stress-Induced TRIB3 Signaling Impairs Macrophage Efferocytosis in Obesity and Atherosclerosis

Dear Dr. Subramanian:

Your manuscript has been carefully evaluated by 5 external reviewers and the editors as an Original Research manuscript. While we are interested in your paper, further minor revision is required before we can accept the manuscript for publication in Circulation Research.

Please read the entire content of this letter and carefully address all of the comments in the reviewers' critiques and all of the formatting concerns described below. Please pay particular attention to the comments made by our statistical and technical reviewers. In particular, our technical editors have detected some similarities between two images in Figure 6. Please carefully check the two images, correct if any errors, and respond to this comment. Please note that the paper cannot be accepted until you have addressed both the reviewers' critiques and all of the formatting issues. Please submit your revision by OCTOBER 13.

All corresponding authors of articles accepted to AHA Journals are required to link an ORCID iD to their profile in the AHA Journal submission system. To register with ORCID or link your profile to your ORCID iD, please go to “Modify Profile/Password” on the submission site homepage (insert journal homepage link), and click the link in the “ORCID” section. Please note that upon resubmission, the corresponding author will be required to have their ORCID iD linked to their profile; processing of the revision will be held until the ORCID link is complete.

The Editors strongly encourage you to adhere to the journal's Statistical Reporting Recommendations in your revision, which can be found here: https://www.ahajournals.org/statistical-recommendations.

In addition, the methods section in the main manuscript should provide sufficient detail for the experiments to be reproduced and should provide sufficient information for the reader to understand the basic methods of the study and to review the fundamental findings in a mechanistic way; as needed, additional methods and information may be included as Supplemental Material.

1. Reviewers' Critiques:

To read the comments to authors from the reviewers, please see below.

If you wish to respond to these suggestions, please include a detailed response to each of the referees' and editors' comments, providing each comment verbatim in bold followed by your response and giving the exact page number(s), paragraph(s), and line number(s) where each revision was made. If you make substantive changes to the

manuscript, please provide a clear description of what you did and where. Additionally, a marked-up version of the revision with the changes highlighted or tracked should be uploaded as a supplemental file.

2. Formatting Issues:

Please ascertain that your revised manuscript adheres to the Instructions to Authors as they appear online at https://www.ahajournals.org/res/author-instructions. Accepted manuscripts are published online ahead of print. Therefore, when submitting the final files of the manuscript and figures, please ensure you have made any essential changes or corrections to content, grammar, and formatting. Please also ensure that author information provided in the online submission system is correct, including author order, proper names, and institutions. Once published ahead of print, you will be unable to make any revisions to the manuscript until you receive your author proofs from the publisher and any changes made to proofs will be reflected in the final print and online journal version of your article.

As your article may be published online upon acceptance, neither the Editorial Office nor the AHA will be responsible for any consequences with regard to intellectual property rights. To safeguard their intellectual property, authors should ensure that appropriate reports of invention and patent applications have been filed before the manuscript is accepted. If you should need to delay publication of your article for any reason, please let the Editorial Office know as soon as possible.

Please provide/address the following areas:

Manuscript Text:
- Please be sure to provide your revised manuscript text in an editable Word Doc file containing all sections of the manuscript, including tables and figure legends. Tables should be embedded within the body of the text as they are mentioned to ensure proper ordering of references.
-Please include a Novelty and Significance section at the end of your Word file. Instructions for the Novelty and Significance section can be found at
https://www.ahajournals.org/res/revised-accepted-manuscripts.
- We request that all authors adhere to the 8,000 word limit. PLEASE NOTE: Word limit includes all sections of the manuscript (Title Page, Abstract, Text, Acknowledgment and COI Sections, References, Figure Legends, and Tables.) Online Supplements and the list of non-standard abbreviations and non-standard acronyms are excluded from the word limit.
Open Access Publication: We request that manuscripts published open access still adhere to the 8,000 word limit as much as possible. However, no excess charges will be rendered above the flat fee for open access publication.
Non Open Access Publication: Options for publishing a manuscript that is above 8,000 words may be found at:
https://www.ahajournals.org/res/revised-accepted-manuscripts under the ‘Costs to Authors’ subheading.
- Please ensure that the title is no more than 80 characters in length (*including spaces*).
- Provide a list of nonstandard abbreviations and non-standard acronyms used in the manuscript text. The list should be included in the manuscript after the abstract and should be entitled "Non-standard Abbreviations and Acronyms." Circulation Research follows AMA style guidelines for standard and non-standard abbreviations.
- Authors are encouraged to provide a detailed, expanded Methods section as an online data supplement, especially if word limit constraints do not allow you to provide a detailed Methods section in the main manuscript. Methods sections should be detailed enough to enable readers to replicate the experiments without consulting previous articles.
- Ensure that the References are formatted according to journal style. Specifically, remove the months, days, and issue numbers of publication.
- Please remove all supplemental figure legends from the main manuscript Word file.

Figures:
- Color figure charges are a flat per page rate of $653 per color page. There are no color figure charges for open access publication beyond the flat fee.

Online Supplement:
- In the manuscript text, following the Acknowledgments, Sources of Funding, & Disclosures section, please include a list of the supplemental materials with a callout to any references that are in the Supplemental Material only.
For example:
Supplemental Materials
Expanded Materials & Methods
Online Figures S1-S5
Online Video 1
Data Set
References 34-39

Other Items:
- Any persons acknowledged by name in the manuscript must send an email to CircRes@circresearch.org citing their permission to be acknowledged.
- Please upload a separate supplement containing a 1-2 line 'lay sentence' similar to those provided for NIH grants to promote your article on social media.
- Recent studies have shown that active engagement in social media is beneficial in advancing your science. Circulation Research encourages all authors to provide their social media handles (BlueSky, LinkedIn & X), if possible.

The Editors strongly encourage you to submit potential cover images. Appropriate figures should be both aesthetically beautiful and scientifically exciting. Potential cover images should be associated with the general topic of the paper, or may be altered/enhanced versions of an original figure within the manuscript. Potential cover figures should have a single panel, with no labels or text of any kind. The figure file should be supplied at exactly 8 1/8" width by 10 7/8" height. Please submit figure initially as a low-resolution PDF. Include a figure legend with the figure. If your figure is chosen, we will request a high-resolution version (minimum of 600 DPI, RGB color format, and TIF or EPS file format).

We look forward to receiving the final revised version of your manuscript as soon as possible. Thank you for contributing to Circulation Research.

Check out the latest episode of Discover CircRes for highlights from the latest issue and expert insights into new research.
New episode every month! https://www.ahajournals.org/res/podcast

Sincerely,

Jane E. Freedman, MD
Editor-in-Chief
Circulation Research
An American Heart Association Journal

****
REVIEWER COMMENTS FOR THE AUTHORS:

Reviewer #1:
The authors addressed my comments.

Reviewer #2:
The authors have addressed all my comments thoroughly. I have no further concerns.

Reviewer #3:
No further comments.

Statistical Reviewer:
1. Please include a supplementary table summarizing the basic demographics of human samples.

2. For Fig. 6C, since both expressions are not normally distributed, I recommend using a non-parametric correlation, e.g. Spearman's rho, instead of Pearson's correlation.

Technical Reviewer:
1. Please include "mice" in the MESH keywords selected through the Circulation Research submission site
2. Per Circulation Research format, please include "What is Known?" and "what New Information Does This Article Contribute?" to highlight the significant and novel findings in this manuscript.
3. Sex: Cell and animal studies were performed in females alone, males alone, or both males and females. Please provide a brief justification as to why a specific sex was studied in the related Method sections.
4. Western blotting: Please ensure that all presented immunoblots have visible molecular weight marker standards BELOW AND ABOVE the target protein band, denoting the location of molecular weight standards. Currently, no molecular weight markers are present on each blot in Figures and Supplemental Figures. All uncropped gels were presented appropriately.
5. Proofig detected some similarities between two images in Figure 6. Please carefully check the two images, correct if any errors, and respond to this comment. [Proofig Report Attached.]

Response to Editors and Reviewers

Please read the entire content of this letter and carefully address all of the comments in the reviewers' critiques and all of the formatting concerns described below. Please pay particular attention to the comments made by our statistical and technical reviewers. In particular, our technical editors have detected some similarities between two images in Figure 6. Please carefully check the two images, correct if any errors, and respond to this comment.

Thank you for the interest in our work and the opportunity to submit a revised version of the manuscript. We have addressed all the Reviewer comments as detailed below. The image similarity arose due to an error during image panel assembly. The correct image is now presented in the revised manuscript.

Statistical Reviewer:

1. Please include a supplementary table summarizing the basic demographics of human samples.

Thanks. This information has been included as Online Table S1.

2. For Fig. 6C, since both expressions are not normally distributed, I recommend using a non-parametric correlation, e.g. Spearman's rho, instead of Pearson’s correlation.

Thanks for the suggestion. The revised Figure 6C demonstrates results from the Spearman’s correlation test. The revised values are: ρ = -0.56 and p = 2.5e-8.

Technical Reviewer:

1. Please include “mice” in the MESH keywords selected through the Circulation Research submission site

Done.

2. Per Circulation Research format, please include “What is Known?” and “what New Information Does This Article Contribute?” to highlight the significant and novel findings in this manuscript.

Done.

3. Sex: Cell and animal studies were performed in females alone, males alone, or both males and females. Please provide a brief justification as to why a specific sex was studied in the related Method sections.

Thanks for the comment. The Methods section has been updated as follows:

Bone marrow chimeric mice generation and induction of atherosclerosis

8-week-old male $Ldlr^{-/-}$ mice were subjected to 10 Gy X-ray irradiation followed by bone marrow reconstitution via intravenous injection of $5x10^6$ bone marrow cells from either WT or TRIB3 KO male mice. The mice were allowed 6 weeks for recovery and the development of bone marrow chimerism followed by induction of atherosclerosis by feeding them a western type diet (SDS western diet, 829100) for 14 weeks. Only male $Ldlr^{-/-}$ mice were used in this study to avoid rejection of sex mis-matched donor bone marrow cells.

In vivo efferocytosis assay in siRNA-transfected lean and obese mice

Six-week-old male C57BL/6J mice were fed a high-fat diet (D12492, Research Diets) for 10 weeks to induce obesity, while control lean mice were maintained on a standard chow diet. Only male mice were used in this experiment, since female C57BL/6J mice show resistance to high-fat diet-induced obesity and associated metabolic phenotypes[34,35].

4. Western blotting: Please ensure that all presented immunoblots have visible molecular weight marker standards BELOW AND ABOVE the target protein band, denoting the location of molecular weight standards. Currently, no molecular weight markers are present on each blot in Figures and Supplemental Figures. All uncropped gels were presented appropriately.

Corrected. All western blots now show markers both above and below the target protein.

5. Proofig detected some similarities between two images in Figure 6. Please carefully check the two images, correct if any errors, and respond to this comment. [Proofig Report Attached.]

We thank the reviewer for this comment. We acknowledge that the highlighted images originated from the same sample and were inadvertently included in the figure panel during image assembly. This error has been corrected, and the appropriate images corresponding to each experimental group are now presented in the revised manuscript.

Manuscript Text:
- Please be sure to provide your revised manuscript text in an editable Word Doc file containing all sections of the manuscript, including tables and figure legends. Tables should be embedded within the body of the text as they are mentioned to ensure proper ordering of references.

Revised manuscript is provided as an editable Word document.

-Please include a Novelty and Significance section at the end of your Word file. Instructions for the Novelty and Significance section can be found at https://www.ahajournals.org/res/revised-accepted-manuscripts.

Novelty and Significance section has been included at the end of the Word document.

- We request that all authors adhere to the 8,000 word limit. PLEASE NOTE: Word limit includes all sections of the manuscript (Title Page, Abstract, Text, Acknowledgment and COI Sections, References, Figure Legends, and Tables.) Online Supplements and the list of non-standard abbreviations and non-standard acronyms are excluded from the word limit.
Open Access Publication: We request that manuscripts published open access still adhere to the 8,000 word limit as much as possible. However, no excess charges will be rendered above the flat fee for open access publication.
Non Open Access Publication: Options for publishing a manuscript that is above 8,000 words may be found at:
https://www.ahajournals.org/res/revised-accepted-manuscripts under the 'Costs to Authors' subheading.

We have moved part of the Methods to online supplemental material to reduce the word count.

- Please ensure that the title is no more than 80 characters in length (*including spaces*).

We have revised the title to "**TRIB3 Links Endoplasmic Reticulum Stress to Impaired Efferocytosis in Atherosclerosis**" The current character count is 85. Further reduction would require abbreviating *Endoplasmic Reticulum* to *ER* or removing key mechanistic detail from the title. We hope the revised title is acceptable to maintain clarity and accuracy.

- Provide a list of nonstandard abbreviations and non-standard acronyms used in the manuscript text. The list should be included in the manuscript after the abstract and should be entitled "Non-standard Abbreviations and Acronyms." Circulation Research follows AMA style guidelines for standard and non-standard abbreviations.

A list of non-standard abbreviations and acronyms are now included.

- Authors are encouraged to provide a detailed, expanded Methods section as an online data supplement, especially if word limit constraints do not allow you to provide a detailed Methods section in the main manuscript. Methods sections should be detailed enough to enable readers to replicate the experiments without consulting previous articles.

Part of the Methods section has been moved to online supplemental material.

- Ensure that the References are formatted according to journal style. Specifically, remove the months, days, and issue numbers of publication.

References have been formatted to Circulation Research output style.

- Please remove all supplemental figure legends from the main manuscript Word file.

Supplemental figure legends have been removed from the manuscript word file.

Figures:
- Color figure charges are a flat per page rate of $653 per color page. There are no color figure charges for open access publication beyond the flat fee.

Ok.

Online Supplement:
- In the manuscript text, following the Acknowledgments, Sources of Funding, & Disclosures section, please include a list of the supplemental materials with a callout to any references that are in the Supplemental Material only.
For example:
Supplemental Materials
Expanded Materials & Methods
Online Figures S1-S5
Online Video 1
Data Set
References 34-39
Done.

Other Items:
- Any persons acknowledged by name in the manuscript must send an email to CircRes@circresearch.org citing their permission to be acknowledged.

Not applicable.

- Please upload a separate supplement containing a 1-2 line 'lay sentence' similar to those provided for NIH grants to promote your article on social media.

Lay sentence document uploaded.

- Recent studies have shown that active engagement in social media is beneficial in advancing your science. Circulation Research encourages all authors to provide their social media handles (BlueSky, LinkedIn & X), if possible.

Document with details uploaded.

\
October 23, 2025

Dr. Manikandan Subramanian
William Harvey Research Institute, Barts and The London School of Medicine and Dentistry, Queen Mary University of London
Centre for Biochemical Pharmacology
Charterhouse Square
London EC1M6BQ
United Kingdom

RE: CIRCRES/2025/326839R2: TRIB3 Links Endoplasmic Reticulum Stress to Impaired Efferocytosis in Atherosclerosis

Dear Dr. Subramanian:

We are pleased to inform you that we have accepted your revised manuscript for publication in Circulation Research as an Original Research manuscript.

All original research articles are published in Circulation Research online ahead of print in PDF proof format within 7-9 days of acceptance, and are accessible through the Online First link at https://www.ahajournals.org/journal/res. Your paper will then appear in the DECEMBER 5, 2025 print and online issue.

The corresponding author will receive the author proof after Online First publication of the manuscript. During the copyediting phase, there may be some changes in phrasing, but there will be no alterations of scientific content. When you receive your author proof, please read, correct, and return it immediately to avoid delay in publication. Please note that it is your responsibility to make yourself available to review the author proof whenever it arrives. If you are not available, publication of your manuscript will be delayed.

IMPORTANT: The Editors strongly encourage you to submit potential cover images. Appropriate figures should be both aesthetically beautiful and scientifically exciting. Potential cover images should be associated with the general topic of the paper, or may be altered/enhanced versions of an original figure within the manuscript. Potential cover figures should have a single panel, with no labels or text of any kind. The figure file should be supplied at exactly 8 1/8" width by 10 7/8" height. The figure file should be provided as a PDF. Higher resolution files will be requested if your figure is chosen.

Meet the First Author Page: Circulation Research encourages up to two first authors of original research articles to contribute a personal profile for our Meet the First Author page. First authors are invited to provide a photo (professional headshot preferred) and a bio for publication. Within 100 words, written in the third person, we ask that they briefly introduce themselves, provide educational background (from undergraduate to post-doctoral fellowship), and identify their main area of research interest. They may also discuss their plans for the future. Please also provide reason(s) for pursuing science in general and how they became involved in the current project as well as any obstacles they encountered as the story developed (i.e. any deviations from the initial central hypothesis). Please provide twitter handles, if available.

Optional information that may be included in the profile can include their experience in building an academic research career as a young scientist, the obstacles they might have faced or factors that might have contributed to

their success, and any other interesting or unique information about themselves, either as scientists or in their lives outside of science. The Editors encourage first authors to use this opportunity to not only talk about their work but also give the readers insight into the everyday lives of young scientists today! Please note that overly long profiles will not be published.
Please submit the first author profile(s) to circres@circresearch.org by FRIDAY, OCTOBER 31.

You will be receiving an Open Access Publication Charge letter from the Journal's Publisher, Wolters Kluwer, when your manuscript is sent to production. The letter will include instructions on how to submit any open access charges. The email will be from no-reply@copyright.com with the subject line 'Please Submit Your Open Access Article Publication Charge(s)'. Please complete payment of the Open Access charges within 48 hours of receipt. Please note that your article cannot be published until payment is received.

Although the Editorial Office will endeavor to notify authors of the anticipated online publication date, neither the Editorial Office nor the AHA will be responsible for any consequences of early online posting with regard to intellectual property rights. To safeguard their intellectual property, authors should ensure that appropriate reports of invention and patent applications have been filed before the manuscript is accepted.

As a service to authors, the publisher (Wolters Kluwer Health/Lippincott Williams & Wilkins) of Circulation Research will identify to PubMed Central articles that require depositing. The Copyright Transfer Agreement provides the primary mechanism for identifying such articles. The secondary mechanism is the "Public Access Policy Funding Disclosure" section of the manuscript metadata completed in the online submission process to Circulation Research. WKH/LWW will transmit the post-print of an article to PubMed Central, which is based on research funded in whole or in part by the US National Institutes of Health and/or the American Heart Association. On NIH request, it remains the legal responsibility of the author(s) to confirm with the PubMed Central the provenance of their manuscript for purposes of deposit. For more information, see https://www.ahajournals.org/public-access-policy.

Circulation Research is the official journal of the Council on Basic Cardiovascular Sciences of the American Heart Association. If you are not already a member of the Council, the Editors strongly encourage you and your coauthors to consider joining. Benefits include substantial discounts on registration at AHA-sponsored meetings and AHA journal subscriptions, early access to hotel reservations for Scientific Sessions, a subscription to the Council newsletter, and eligibility to submit late-breaking basic science abstracts for presentation at Sessions. Find out more about the benefits of AHA Membership at https://professional.heart.org/membership.

Congratulations on this outstanding work, and thank you for contributing to Circulation Research.

Check out the latest episode of Discover CircRes for highlights from the latest issue and expert insights into new research.
New episode every month! https://www.ahajournals.org/res/podcast

Sincerely,

Kathryn Moore, PhD
Senior Associate Editor

Jane E. Freedman, MD
Editor-in-Chief
Circulation Research
An American Heart Association Journal
